# Polyfunctionalized α-Phenyl-tert-butyl(benzyl)nitrones: Multifunctional Antioxidants for Stroke Treatment

**DOI:** 10.3390/antiox11091735

**Published:** 2022-08-31

**Authors:** Daniel Diez-Iriepa, Damijan Knez, Stanislav Gobec, Isabel Iriepa, Cristóbal de los Ríos, Isaac Bravo, Francisco López-Muñoz, José Marco-Contelles, Dimitra Hadjipavlou-Litina

**Affiliations:** 1 Laboratory of Medicinal Chemistry, Institute of Organic Chemistry (CSIC), Juan de la Cierva 3, 28006 Madrid, Spain; 2 Departamento de Química Orgánica y Química Inorgánica, Universidad de Alcalá, Ctra. Madrid-Barcelona Km 33.6, 28871 Alcalá de Henares, Spain; 3 Faculty of Pharmacy, University of Ljubljana, 1000 Ljubljana, Slovenia; 4 Institute of Chemical Research Andrés M. del Río, Alcalá University, 28805 Alcalá de Henares, Spain; 5 Institute of Health Research, Hospital Universitario de la Princesa, 28006 Madrid, Spain; 6 Department of Pharmacology, Autonomous University of Madrid, 28034 Madrid, Spain; 7 Faculty of Health, Camilo José Cela University of Madrid (UCJC), Castillo Alarcon 49, 28692 Madrid, Spain; 8 Neuropsychopharmacology Unit, “Hospital 12 de Octubre” Research Institute, 28041 Madrid, Spain; 9 Center for Biomedical Network Research on Rare Diseases (CIBERER), CIBER, ISCIII, 28040 Madrid, Spain; 10 Department of Pharmaceutical Chemistry, Faculty of Health Sciences, School of Pharmacy, Aristotle University of Thessaloniki, 54124 Thessaloniki, Greece

**Keywords:** antioxidants, butyrylcholinesterase, free radical scavengers, neuroprotection, α-phenyl-tert-butyl(benzyl)nitrones

## Abstract

Nowadays, most stroke patients are treated exclusively with recombinant tissue plasminogen activator, a drug with serious side effects and limited therapeutic window. For this reason, and because of the known effects of oxidative stress on stroke, a more tolerable and efficient therapy for stroke is being sought that focuses on the control and scavenging of highly toxic reactive oxygen species by appropriate small molecules, such as nitrones with antioxidant properties. In this context, herein we report here the synthesis, antioxidant, and neuroprotective properties of twelve novel polyfunctionalized α-phenyl-*tert*-butyl(benzyl)nitrones. The antioxidant capacity of these nitrones was investigated by various assays, including the inhibition of lipid peroxidation induced by AAPH, hydroxyl radical scavenging assay, ABTS^+^-decoloration assay, DPPH scavenging assay, and inhibition of soybean lipoxygenase. The inhibitory effect on monoamine oxidases and cholinesterases and inhibition of β-amyloid aggregation were also investigated. As a result, (*Z*)-*N*-benzyl-1-(2-(3-(piperidin-1-yl)propoxy)phenyl)methanimine oxide (**5**) was found to be one of the most potent antioxidants, with high ABTS^+^ scavenging activity (19%), and potent lipoxygenase inhibitory capacity (IC_50_ = 10 µM), selectively inhibiting butyrylcholinesterase (IC_50_ = 3.46 ± 0.27 µM), and exhibited neuroprotective profile against the neurotoxicant okadaic acid in a neuronal damage model. Overall, these results pave the way for the further in-depth analysis of the neuroprotection of nitrone **5** in in vitro and in vivo models of stroke and possibly other neurodegenerative diseases in which oxidative stress is identified as a critical player.

## 1. Introduction

There is an overwhelming consensus among neuroscientists that oxidative stress is one of the most important biological events underlying a number of diseases of ageing, including stroke, Alzheimer’s disease (AD), and Parkinson’s disease (PD) [1].

Nowadays, stroke patients in clinics are treated exclusively with recombinant tissue plasminogen activator (rtPA) [2], a drug with serious side effects [3] and limited therapeutic window [4]. Therefore, a more tolerable and efficient therapy for stroke is being explored in different laboratories, focusing on the control and capture of highly toxic reactive oxygen species (ROS) [5]. AD is a progressive, irreversible disorder [6], characterized by massive and significant neuronal cell death [7]. PD is a chronic neurodegenerative disease affecting 1% of elderly people over 60 years of age [8], caused by a dopamine deficiency that produces the well-known symptoms of this pathology: tremor, rigidity, bradykinesia, and postural instability [9]. To date, most approved therapies for the treatment of PD aim to increase striatal dopamine levels [10].

**Figure 1:**
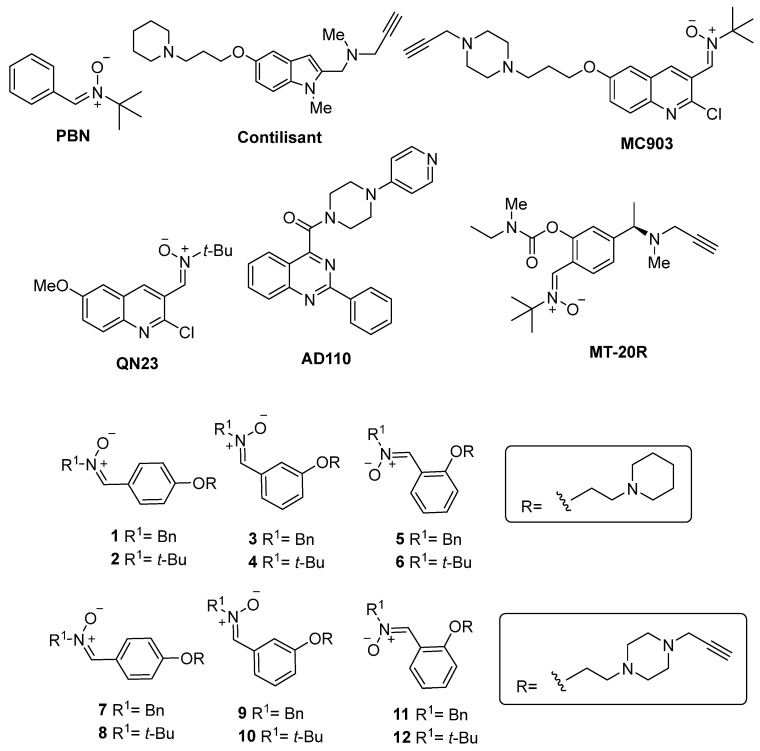
Structures of **PBN**, **Contilisant**, **QN23**, **MC903**, and the **PBN**-based nitrones **1**–**12**.

In this context, synthetic and antioxidant nitrones have been selected, analyzed, and used in the treatment of stroke in the past [11]. This is true for *α*-phenyl-*N*-*tert*-butylnitrone (PBN) (Figure 1), a simple and well-known drug for the prevention and reversal of cerebral ischemia in suitable animal models [12]. The ongoing interest in nitrones for the potential therapy of stroke is very well-documented in the current literature [13], and our research team is strongly interested and involved in several projects aimed at finding new and more efficient nitrones for the improved treatment of stroke [14].

The neuroprotective activity, combined with the potent antioxidant capacity of polyfunctionalized nitrones and their clinical application for unique and multivalent therapy of stroke and neurodegenerative diseases, is rarely reported in the literature. Recently, Sun et al. [15] described the pharmacological profile of the neuroimmunomodulator **AD110** (Figure 1) for the treatment of AD and stroke, and Liu et al. [16] reported **MT-20R** (Figure 1), a substituted **PBN** bearing pharmacophoric groups of monoamine oxidase (MAO) and cholinesterase (ChE) inhibitors. Our group has recently disclosed a series of hybrid polyfunctionalized nitrones, resulting from the juxtaposition of **Contilisant** (Figure 1) [17,18], an advanced lead compound for the potential therapy of AD, and the potent antioxidant and neuroprotective quinolylnitrone **QN23** [19]. Hybrid **MC903** (Figure 1) was developed as a small molecule quinolylnitrone for the potential dual therapy of stroke and AD and demonstrated potent neuroprotective effects in: (1) primary cortical neurons under oxygen–glucose deprivation/normoglycemic reoxygenation conditions, as an experimental ischemia model and (2) neuronal line cells treated with rotenone/oligomycin A, okadaic acid, or β-amyloid peptide Aβ_25–35_, as a model for toxic insults found among the effects of AD [20].

Based on the previously reported results [20], we have demonstrated the replacement of the quinolylnitrone motif with the (*Z*)-*N*-*tert*-butyl-1-phenylmethanimine oxide (**PBN**) scaffold and resulting changes in antioxidant and neuroprotective properties. We designed and synthesized *para*-, *meta*-, and *ortho*-substituted **PBN**-piperidinepropoxy- and *N*-propargyl-piperazinepropoxy-substituted nitrones **1**–**12** (Figure 1). As a result of this investigation, we identified PBN-nitrone **5** (Figure 1) as a potent anti-inflammatory ligand, due to its high lipoxygenase (LOX) inhibitory activity, and selective nanomolar butyrylcholinesterase (BChE) inhibitor and efficient neuroprotective agent.

## 2. Materials and Methods

### 2.1. General Methods

Compound purification was performed by column chromatography with Merck silica gel (40–63 µm) or flash chromatography (Biotage Isolera One, Uppsala, Sweden), as well as the adequate eluent for each compound. Reactions were monitored by thin layer chromatography (TLC), and compounds were detected under UV light (λ = 254 nm) and by using ethanolic solution of vanillin or ninhydrin. Melting points were determined using a Reichert Thermo Galen Kofler block and were uncorrected. ^1^H-NMR and ^13^C-NMR spectra were obtained in Bruker Avance 300 (300 MHz) and Bruker Avance 400 III HD (400 MHz) spectrometers. Samples were dissolved in CDCl_3_ or DMSO-*d_6_*, and TMS was used as internal standard for ^1^H NMR spectra. In ^13^C NMR spectra, central signals of solvent CDCl_3_ (77.0 ppm) and DMSO-*d* (39.5 ppm) were used as references. Chemical shifts (δ) are given in *ppm*, and coupling constants (*J*) are reported in Hz. Signal multiplicity is abbreviated as: singlet (s), doublet (d), triplet (t), pentuplet (p), and multiplet (m). IR spectra were recorded on a Perkin-Elmer Spectrum One B spectrometer. Low-resolution mass spectra were recorded on an Agilent HP 1100 LC/MS spectrometer, and high-resolution mass spectrometry (Exact Mass) was performed in an AGILENT 6520 Accurate-Mass QTOF LC/MS spectrometer. Elemental analysis was performed on elementary chemical analyzer (LECO CHNS-932).

#### 2.1.1. General Method for O-alkylation (Method A)

Commercially available aldehyde was dissolved in mixture of CHCl_3_/H_2_O (5/1, *v*/*v*) and K_2_CO_3_ (3 equiv) and 1-(3-chloropropyl)piperidine (**16**) (1.5 equiv) or 1-(3-chloropropyl)-4-(prop-2-yn-1-yl)piperazine (**23**) (1.5 equiv) were added. The mixture was stirred at 80 °C for 1 d. When the reaction was finished (TLC analysis), the mixture was extracted with CH_2_Cl_2_, dried over Na_2_SO_4_, filtered, the solvent was evaporated, and the crude mixture was purified by column chromatography using the indicated mixtures of solvents.

#### 2.1.2. General Method for Synthesis of Nitrones (Method B)

NaHCO_3_ (1.5 equiv), Na_2_SO_4_ (2 equiv), and *N*-benzylhydroxylamine hydrochloride (1.5 equiv) were added to a solution of the corresponding aldehyde in THF (0.1 M). The reaction mixture was stirred at room temperature (rt) for the shown time in each case. The solvent was removed under reduced pressure, and the crude mixture purified by column chromatography using the indicated mixture of solvents. (**Method C**). AcONa (1.5 equiv), Na_2_SO_4_ (2 equiv) and *N*-*tert*-butylhydroxylamine hydrochloride (1.5 equiv) were added to a solution of the corresponding aldehyde in EtOH (0.1 M). The reaction was irradiated in a microwave apparatus at 90 °C for the time indicated for each reaction. Then, the solvent was removed, and the crude mixture purified by column chromatography using the indicated mixture of solvents.

##### 4-(3-(Piperidin-1-yl)propoxy)benzaldehyde (**17**) [21]

Following general **method A**, the reaction of commercial 4-hydroxybenzaldehyde (**13**) (207.4 mg, 1.7 mmol) with K_2_CO_3_ (703.8 mg, 5.2 mmol) and 1-(3-chloropropyl)piperidine (**16**) (358 mg, 2.21 mmol) in CHCl_3_/H_2_O (5/1, 11 mL), after column chromatography (CH_2_Cl_2_/MeOH 3%), afforded carbaldehyde **17** (210.3 mg, 50%) as an oil: IR (film) ν 2931, 1690, 1600, 1509, 1258, 1159 cm^−1^; ^1^H NMR (400 MHz, CDCl_3_) δ 9.81 (s, 1H), 7.78–7.70 (m, 2H), 7.00–6.89 (m, 2H), 4.03 (t, *J* = 6.4 Hz, 2H), 2.42 (t, *J* = 7.6 Hz, 2H), 2.40–2.26 (m, 4H), 2.00–1.89 (m, 2H), 1.58–1.48 (m, 4H), 1.44–1.34 (m, 2H); ^13^C NMR (101 MHz, CDCl_3_) δ 190.8, 164.2, 132.0 (2C), 129.8, 114.8 (2C), 66.9, 55.7, 54.6 (2C), 26.6, 25.9 (2C), 24.4. HRMS (ESI_ACN). Calcd. for C_15_H_21_NO_2_: 247.1572; found: 247.1571.

##### (Z)-*N*-Benzyl-1-(4-(3-(piperidin-1-yl)propoxy)phenyl)methanimine oxide (**1**)

Following general **method B**, the reaction of compound **17** (98.9 mg, 0.4 mmol) with NaHCO_3_ (50.4 mg, 0.6 mmol), Na_2_SO_4_ (113.6 mg, 0,80 mmol), and *N*-benzylhydroxylamine hydrochloride (95.75 mg, 0.6 mmol), in THF (4 mL) for 5 min, after column chromatography (MeOH/CH_2_Cl_2_, 6%), gave nitrone **1** (140.6 mg, 99%): mp 105–7 °C; IR (KBr) ν 2926, 1599, 1246, 1162, 1026 cm^−1^; ^1^H NMR (400 MHz, CDCl_3_) δ 8.12 (d, *J* = 8.9 Hz, 2H), 7.51–7.16 (m, 6H), 6.83 (d, *J* = 8.9 Hz, 2H), 4.96 (s, 2H), 3.98 (t, *J* = 6.3 Hz, 2H), 2.69–2.25 (m, 6H), 2.11–1.92 (m, 2H), 1.61 (p, *J* = 5.9 Hz, 4H), 1.50–1.32 (m, 2H); ^13^C NMR (101 MHz, CDCl_3_) δ 160.4, 133.9, 133.4, 130.6 (2C), 129.2 (2C), 128.96 (2C), 128.90, 123.4, 114.6, 114.3 (2C), 70.7, 66.3, 55.8, 54.4 (2C), 26.1, 25.3, 24.0. HRMS (ESI_ACN). Calcd. for C_22_H_28_N_2_O_2_: 352.2151; found: 352.2168.

##### 3-(3-(Piperidin-1-yl)propoxy)benzaldehyde (**18**) [21]

Following general **method A**, the reaction of 3-hydroxybenzaldehyde (**14**) (207.4 mg, 1.7 mmol) with K_2_CO_3_ (703.8 mg, 5.2 mmol) and 1-(3-chloropropyl)piperidine (**16**) (358 mg, 2.21 mmol), in CHCl_3_/H_2_O (5/1, 11 mL), after column chromatography (CH_2_Cl_2_/MeOH 4%), afforded carbaldehyde **18** (393.1 mg, 93%) as an oil: IR (film) ν 2944, 1694, 1586, 1455, 1263 cm^−1^; ^1^H NMR (400 MHz, CDCl_3_) δ 9.90 (s, 1H), 7.48–7.33 (m, 2H), 7.29 (dd, *J* = 3.1, 1.1 Hz, 1H), 7.08 (ddd, *J* = 7.4, 2.8, 1.8 Hz, 1H), 4.08 (t, *J* = 5.6 Hz, 2H), 3.61–3.45 (m, 2H), 3.18–3.05 (m, 2H), 2.75–2.54 (m, 2H), 2.56–2.38 (m, 2H), 2.38–2.21 (m, 2H), 1.95–1.64 (m, 4H); ^13^C NMR (101 MHz, CDCl_3_) δ 191.9, 158.8, 137.8, 130.3, 123.9, 121.3, 113.0, 65.2, 55.2, 53.5 (2C), 23.8, 22.6 (2C), 22.1. HRMS (ESI_ACN). Calcd. for C_15_H_21_NO_2_: 247.1572; found: 247.1574.

##### (Z)-*N*-Benzyl-1-(3-(3-(piperidin-1-yl)propoxy)phenyl)methanimine oxide (**3**)

Following general **method B**, the reaction of compound **18** (175.6 mg, 0.71 mmol) with NaHCO_3_ (92.4 mg, 1.1 mmol), Na_2_SO_4_ (201.6 mg, 1.42 mmol) and *N*-benzylhydroxylamine hydrochloride (175.6 mg, 1.1 mmol), in THF (7 mL) for 5 min, after column chromatography (MeOH/CH_2_Cl_2_, 5%), gave nitrone **3** (172.8 mg, 69%): mp 60–2 °C; IR (KBr) ν 2933, 1584, 1440, 1183 cm^−1^; ^1^H NMR (400 MHz, CDCl_3_) δ 8.09 (dd, *J* = 2.6, 1.5 Hz, 1H), 7.45–7.31 (m, 6H), 7.25–7.16 (m, 2H), 6.88 (ddd, *J* = 8.3, 2.6, 1.1 Hz, 1H), 4.98 (s, 2H), 3.96 (t, *J* = 6.3 Hz, 2H), 2.58–2.28 (m, 6H), 2.05–1.85 (m, 2H), 1.57 (p, *J* = 5.6 Hz, 4H), 1.47–1.32 (m, 2H); ^13^C NMR (101 MHz, CDCl_3_) δ 158.9, 134.3, 133.2, 131.5, 129.3 (2C), 129.2, 129.03, 129.01 (2C), 121.6, 117.7, 113.2, 71.3, 66.4, 55.9, 54.5 (2C), 26.4, 25.5 (2C), 24.1. HRMS (ESI_ACN). Calcd. for C_22_H_28_N_2_O_2_: 352.2151; found: 352.2153.

##### 2-(3-(Piperidin-1-yl)propoxy)benzaldehyde (**19**) [21]

Following general **method A**, the reaction of 2-hydroxybenzaldehyde (**15**) (207.4 mg, 1.7 mmol) with K_2_CO_3_ (703.8 mg, 5.2 mmol) and 1-(3-chloropropyl)piperidine (**16**) (358 mg, 2.21 mmol) in CHCl_3_/H_2_O (5/1, 11 mL), after column chromatography (CH_2_Cl_2_/MeOH 4%), to afford carbaldehyde **19** (401.5 mg, 95%) as an oil: IR (film) ν 2931, 1686, 1597, 1455, 1284, 1239, 754 cm^−1^; ^1^H NMR (400 MHz, CDCl_3_) δ 10.43 (s, 1H), 7.79–7.66 (m, 1H), 7.45 (ddd, *J* = 8.3, 7.4, 1.9 Hz, 1H), 7.03–6.82 (m, 2H), 4.06 (t, *J* = 6.3 Hz, 2H), 2.49–2.40 (m, 2H), 2.33 (br s, 4H), 2.05–1.91 (m, 2H), 1.52 (p, *J* = 5.7 Hz, 4H), 1.43–1.31 (m, 2H); ^13^C NMR (101 MHz, CDCl_3_) δ 189.8, 161.5, 135.9, 128.2, 124.9, 120.5, 112.6, 67.0, 55.8, 54.7 (2C), 26.7, 25.9 (2C), 24.4. HRMS (ESI_ACN). Calcd. for C_15_H_21_NO_2_: 247.1572; found: 247.1574.

##### (Z)-*N*-Benzyl-1-(2-(3-(piperidin-1-yl)propoxy)phenyl)methanimine oxide (**5**)

Following general **method B**, the reaction of compound **19** (197.9 mg, 0.8 mmol) with NaHCO_3_ (100.8 mg, 1.2 mmol), Na_2_SO_4_ (227.2 mg, 1.6 mmol) and *N*-benzylhydroxylamine hydrochloride (191.5 mg, 1.2 mmol), in THF (7 mL), for 5 min, after column chromatography (MeOH/CH_2_Cl_2_, 4%), gave nitrone **5** (232.5 mg, 82%): mp 59–61 °C; IR (KBr) ν 2927, 1591, 1464, 1243, 1153 cm^−1^; ^1^H NMR (400 MHz, CDCl_3_) δ 9.18 (dd, *J* = 8.0, 1.8 Hz, 1H), 7.82 (s, 1H), 7.48–7.41 (m, 2H), 7.39–7.22 (m, 4H), 6.92 (td, *J* = 8.0, 1.3 Hz, 1H), 6.78 (dd, *J* = 8.4, 1.3 Hz, 1H), 5.00 (s, 2H), 3.95 (t, *J* = 6.3 Hz, 2H), 2.56–2.21 (m, 6H), 2.01–1.82 (m, 2H), 1.56 (p, *J* = 5.6 Hz, 4H), 1.47–1.29 (m, 2H); ^13^C NMR (101 MHz, CDCl_3_) δ 156.3, 133.7, 131.5, 129.2 (2C), 129.0, 128.9 (2C), 128.8, 128.7, 120.6, 119.6, 110.7, 71.5, 66.7, 55.9, 54.7 (2C), 26.7, 25.8 (2C), 24.3. HRMS (ESI_ACN). Calcd. for C_22_H_28_N_2_O_2_: 352.2151; found: 352.2149.

##### (Z)-*N*-*tert*-Butyl-1-(4-hydroxyphenyl)methanimine oxide (**20**) [22]

Following general **method C**, the reaction of 4-hydroxybenzaldehyde (**13**) (207.4 mg, 1.7 mmol) with AcONa (209.1 mg, 2.55 mmol), Na_2_SO_4_ (482.8 mg, 3.4 mmol) and *N*-*tert*-butylhydroxylamine hydrochloride (320.3 mg, 2.55 mmol), in EtOH (10 mL), for 5 h, after column chromatography (MeOH/CH_2_Cl_2_, 3%), afforded nitrone **20** (320 mg, 97%): mp 214–6 °C; IR (KBr) ν 1604, 1572, 1293, 1164, 1086 cm^−1^; ^1^H NMR (400 MHz, CDCl_3_) δ 8.09 (d, *J* = 8.9 Hz, 2H), 7.41 (s, 1H), 6.81 (d, *J* = 8.9 Hz, 2H), 1.53 (s, 9H), (the OH signal was not detected); ^13^C NMR (101 MHz, CDCl_3_) δ 177.9, 131.5 (2C), 115.7 (2C), 57.1, 24.8 (3C), 28.3, 21.8. HRMS (ESI_ACN). Calcd. for C_11_H_15_NO_2_: 193.1108; found: 193.1103.

##### (Z)-*N*-*tert*-Butyl-1-(4-(3-(piperidin-1-yl)propoxy)phenyl)methanimine oxide (**2**)

Following general **method A**, the reaction of nitrone **20** (174 mg, 0.9 mmol) with K_2_CO_3_ (372.6 mg, 2.7 mmol) and 1-(3-chloropropyl)piperidine (**16**) (189.5 mg, 1.17 mmol) in CHCl_3_/H_2_O (5/1, 11 mL), after column chromatography (CH_2_Cl_2_/MeOH, 6%), gave nitrone **2** (248.3 mg, 87%): mp 80–2 °C; IR (KBr) ν 2940, 1604, 1507, 1256, 1174, 1105 cm^−1^; ^1^H NMR (400 MHz, CDCl_3_) δ 8.20 (d, *J* = 9.1 Hz, 2H), 7.39 (s, 1H), 6.85 (d, *J* = 9.1 Hz, 2H), 3.98 (t, *J* = 6.4 Hz, 2H), 2.52–2.32 (m, 6H), 2.08–1.85 (m, 2H), 1.61–1.49 (m, 13H), 1.43–1.31 (m, 2H); ^13^C NMR (101 MHz, CDCl_3_) δ 160.3, 130.7 (2C), 129.5, 123.9, 114.3 (2C), 70.0, 66.5, 55.9, 54.6 (2C), 28.3 (3C), 26.6, 25.8 (2C), 24.3. HRMS (ESI_ACN). Calcd. for C_19_H_30_N_2_O_2_: 318.2307; found: 318.2306.

##### (Z)-*N*-*tert*-Butyl-1-(3-hydroxyphenyl)methanimine oxide (**21**) [22]

Following general **method C**, the reaction of 3-hydroxybenzaldehyde (**14**) (207.4 mg, 1.7 mmol) with AcONa (209.1 mg, 2.55 mmol), Na_2_SO_4_ (482.8 mg, 3.4 mmol) and *N*-*tert*-butylhydroxylamine hydrochloride (320.28 mg, 2.55 mmol), in EtOH (10 mL), for 5 h, after column chromatography (MeOH/CH_2_Cl_2_, 3%), afforded nitrone **21** (175.3 mg, 53%): mp 173–5 °C; IR (KBr) ν 1591, 1448, 1269, 1176, 1097 cm^−1^; ^1^H NMR (400 MHz, CDCl_3_) δ 8.99 (d, *J* = 1.0 Hz, 1H), 7.50 (s, 1H), 7.19–7.14 (m, 1H), 6.91–6.78 (m, 2H), 1.57 (s, 9H) (the OH signal was not detected); ^13^C NMR (101 MHz, CDCl_3_) δ 157.7, 133.2, 131.0, 129.2, 122.2, 118.9, 115.2, 70.9, 28.3 (3C). HRMS (ESI_ACN). Calcd. for C_11_H_15_NO_2_: 193.1101; found: 193.1101.

##### (Z)-*N*-*tert*-Butyl-1-(3-(3-(piperidin-1-yl)propoxy)phenyl)methanimine oxide (**4**)

Following general **method A**, the reaction of nitrone **21** (160 mg, 0.83 mmol) with K_2_CO_3_ (343.62 mg, 2.49 mmol) and 1-(3-chloropropyl)piperidine (**16**) (174.8 mg, 1.08 mmol) in CHCl_3_/H_2_O (5/1, x mL), after column chromatography (CH_2_Cl_2_/MeOH, 6%), gave nitrone **4** (125 mg, 43%) as an oil: IR (film) ν 2931, 1578, 1440, 1360, 1183, 1120 cm^−1^; ^1^H NMR (400 MHz, CDCl_3_) δ 8.23 (d, *J* = 2.1 Hz, 1H), 7.45 (s, 1H), 7.42 (d, *J* = 7.6 Hz, 1H), 7.26–7.19 (m, 1H), 6.88 (dd, *J* = 8.3, 3.0 Hz, 1H), 4.00 (t, *J* = 6.3 Hz, 2H), 2.48–2.28 (m, 6H), 1.92 (p, *J* = 6.6 Hz, 2H), 1.54 (br s, 13H), 1.43–1.32 (m, 2H); ^13^C NMR (101 MHz, CDCl_3_) δ 159.0, 132.2, 129.9, 129.1, 121.8, 117.5, 113.1, 70.9, 66.5, 56.0, 54.6 (2C), 28.3 (3C), 26.7, 25.8 (2C), 24.4. HRMS (ESI_ACN). Calcd. for C_19_H_30_N_2_O_2_: 318.2307; found: 318.2310.

##### (Z)-*N*-*tert*-Butyl-1-(2-hydroxyphenyl)methanimine oxide (**22**) [22]

Following general **method C**, the reaction of 2-hydroxybenzaldehyde (**15**) (207.4 mg, 1.7 mmol), AcONa (209.1 mg, 2.55 mmol), Na_2_SO_4_ (482.8 mg, 3.4 mmol) and *N*-*tert*-butylhydroxylamine hydrochloride (320.28 mg, 2.55 mmol), in EtOH (10 mL), for 5 h, after column chromatography (MeOH/CH_2_Cl_2_, 3%), afforded nitrone **22** (318 mg, 97%): mp 90–2 °C; IR (KBr) ν 1578, 1455, 1284, 1002 cm^−1^; ^1^H NMR (400 MHz, CDCl_3_) δ 7.65 (s, 1H), 7.30 (ddd, *J* = 8.5, 7.2, 1.7 Hz, 1H), 7.03 (dd, *J* = 7.2, 1.8 Hz, 1H), 6.90 (dd, *J* = 8.5, 1.4 Hz, 1H), 6.77 (td, *J* = 8.5, 7.1, 1.3 Hz, 1H), 1.56 (s, 9H); ^13^C NMR (101 MHz, CDCl_3_) δ 159.7, 137.2, 133.6, 132.2, 120.2, 118.8, 117.0, 70.1, 28.3 (3C). HRMS (ESI_ACN). Calcd. for C_11_H_15_NO_2_: 193.1103; found: 193.1107.

##### (Z)-*N*-*tert*-Butyl-1-(2-(3-(piperidin-1-yl)propoxy)phenyl)methanimine oxide (**6**)

Following general **method A**, the reaction of nitrone **22** (160 mg, 0.83 mmol) with K_2_CO_3_ (343.62 mg, 2.49 mmol) and 1-(3-chloropropyl)piperidine (174.8 mg, 1.08 mmol) in CHCl_3_/H_2_O (5/1, 5 mL), after column chromatography (CH_2_Cl_2_/MeOH, 6%), gave nitrone **6** (112.9 mg, 39%) as an oil: IR (film) ν 2931, 1593, 1451, 1239, 1127, 1107 cm^−1^; ^1^H NMR (400 MHz, CDCl_3_) δ 9.26 (d, *J* = 8.0 Hz, 1H), 8.00 (s, 1H), 7.26 (t, *J* = 8.0 Hz, 1H), 6.94 (t, *J* = 8.0 Hz, 1H), 6.81 (d, *J* = 8.0 Hz, 1H), 3.99 (t, *J* = 6.3 Hz, 2H), 2.55–2.31 (m, 6H), 2.06–1.90 (m, 2H), 1.64–1.49 (m, 13H), 1.47–1.30 (m, 2H); ^13^C NMR (101 MHz, CDCl_3_) δ 156.5, 131.1, 128.6, 124.3, 120.7, 120.2, 110.6, 70.9, 66.7, 56.2, 54.7 (2C), 28.4 (3C), 26.7, 25.8 (2C), 24.3. HRMS (ESI_ACN). Calcd. for C_19_H_30_N_2_O_2_: 318.2307; found: 318.2305.

##### 1-(3-Chloropropyl)-4-(prop-2-yn-1-yl)piperazine (**23**) [20]

To a solution of commercial *N*-*tert*-butoxycarbonylpiperazine (594 mg, 3.19 mmol) in dry CH_2_Cl_2_ (15 mL), cooled at 0 °C, TEA (0.6 mL, 4.15 mmol) was added. Then, commercial l-bromo-3-chloropropane (4.15 moles, 0.41 mL) was added, and the mixture was heated at 50 °C. After 3 h, the same amount of TEA and of 1-bromo-3-chloropropane was added, and the reaction was refluxed for 24 h. Then, the solvent was removed at reduced pressure, and the raw product was dissolved in CH_2_Cl_2_ (20 mL) and washed with water (10 mL). The organic phase is washed with brine (10 mL), dried with Na_2_SO_4_, filtered, and the solvent removed at reduced pressure to afford *tert*-butyl 4-(3-chloropropyl)piperazine-1-carboxylate [23] (665.5 mg, 80%; ^1^H NMR (300 MHz, CDCl_3_) δ 3.60 (t, *J* = 6.5 Hz, 2H), 3.41 (q, *J* = 5.0 Hz, 4H), 2.53–2.46 (m, 2H), 2.45–2.34 (m, 4H), 2.01–1.87 (m, 2H), 1.45 (s, 9H)). *N*-*tert*-Butoxycarbonyl-*N*-(3-chloropropyl) piperazine (665.5 mg, 2.54 mmol) was dissolved at 0 °C in a solution of AcOEt saturated with HCl(g). The mixture was left in an ice bath for 1 h and 1 d at rt. Then, the solvent was removed at reduced pressure, treated with ethyl ether, and filtered to yield 1-(3-chloropropyl) piperazine dihydrochloride [24] (456.8 mg, 77%; ^1^H NMR (300 MHz, DMSO-d_6_) δ 3.74 (t, *J* = 6.5 Hz, 2H), 3.70–3.37 (m, 8H), 3.27–3.16 (m, 2H), 2.29–2.08 (m, 2H) (the NH signals could not be detected), which was used without further purifications for the successive reaction). To a solution of 1-(3-chloropropyl) piperazine dihydrochloride (702 mg, 3 mmol, 1 equiv), triethylamine (TEA) (0.84 mL, 6 mmol, and 2 equiv) in dry CH_2_Cl_2_ (5 mL), cooled at 0 °C, propargyl bromide (0.81 mL, 9 mmol, and 3 equiv) was added over 30 min, under argon. The mixture was stirred at rt for 24 h and treated with an aq. saturated solution of NaHCO_3_ (10 mL); the organic layer was separated, washed with brine, dried with Na_2_SO_4_, filtered, and the solvent was removed. The crude was purified by column chromatography (CH_2_Cl_2_/MeOH 1–2%) to yield compound **23** (312 mg, 52%) as a white solid: mp > 230 °C; IR (KBr) ν 3330, 3219, 1612, 1503, 1223 cm^−1^; ^1^H NMR (400 MHz, CDCl_3_) δ 3.53 (t, *J*= 6.6 Hz, 2H, ClCH_2_), 3.23 (d, *J*= 2.5 Hz, 2H), 2.47–2.39 (m, 10H), 2.18 (t, *J*= 2.5 Hz, 1H), 1.88 (p, *J* = 6.7 Hz, 2H); ^13^C NMR (101 MHz, CDCl_3_) δ 78.8, 73.2, 56.4, 55.4, 53.1, 51.9 (4C), 46.8, 29.9. HRMS (ESI_ACN). Calcd. for C_10_H_17_ClN_2_: 200.1080; found: 200.1080.

##### 4-(3-(4-(Prop-2-yn-1-yl) piperazin-1-yl) propoxy)benzaldehyde enzaldehyde (**24**)

Following general **method A**, the reaction of 4-hydroxybenzaldehyde (13) (165.8 mg, 1.36 mmol) with K_2_CO_3_ (562.1 mg, 4 mmol) and 1-(3-chloropropyl)-4-(prop-2-yn-1-yl)piperazine (23) (353 mg, 1.77 mmol) in CHCl_3_/H_2_O (5/1, 11 mL), after column chromatography (CH_2_Cl_2_/MeOH, 2%), afforded carbaldehyde 24 (209.5 mg, 41%): mp 61–3 °C; IR (KBr) ν 2940, 2816, 1686, 1600, 1258, 1159 cm^−1^; 1H NMR (400 MHz, CDCl_3_) δ 9.81 (s, 1H), 7.76 (d, *J* = 8.9 Hz, 2H), 6.93 (d, *J* = 8.9 Hz, 2H), 4.04 (t, *J* = 6.3 Hz, 2H), 3.24 (d, *J* = 2.4 Hz, 2H), 2.71–2.33 (m, 10H), 2.19 (t, *J* = 2.4 Hz, 1H), 2.04–1.88 (m, 2H); ^13^C NMR (101 MHz, CDCl_3_) δ 190.8, 164.1, 132.0 (2C), 129.9, 114.8 (2C), 78.7, 73.3, 66.5, 54.8 (2C), 53.0 (2C), 51.7, 46.8, 26.4. HRMS (ESI_ACN). Calcd. for C_17_H_22_N_2_O_2_: 286.1681; found: 286.1678.

##### (Z)-*N*-Benzyl-1-(4-(3-(4-(prop-2-yn-1-yl) piperazin-1-yl) propoxy) phenyl) methanimine oxide xide (**7**)

Following general **Method B**, the reaction of compound **24** (108.9 mg, 0.38 mmol) with NaHCO_3_ (47.9 mg, 0.57 mmol), Na_2_SO_4_ (108 mg, 0.76 mmol), and *N*-benzylhydroxylamine hydrochloride (91 mg, 0.57 mmol), in THF (4 mL) for 30 min, after column chromatography (MeOH/CH_2_Cl_2_, 4%), afforded nitrone **7** (90,5 mg, 59%): mp 118–9 °C; IR (KBr) ν 2948, 2810, 1600, 1265, 1136 cm^−1^; ^1^H NMR (500 MHz, CDCl_3_) δ 8.26–8.13 (m, 2H), 7.54–7.35 (m, 5H), 7.31 (s, 1H), 6.97–6.85 (m, 2H), 5.03 (s, 2H), 4.06 (t, *J* = 6.4 Hz, 2H), 3.31 (d, *J* = 2.4 Hz, 2H), 2.76–2.43 (m, 10H), 2.25 (t, *J* = 2.4 Hz, 1H), 2.04–1.94 (m, 2H); ^13^C NMR (126 MHz, CDCl_3_) δ 160.6, 133.9, 133.4, 130.6 (2C), 129.2 (2C), 128.95 (2C), 128.88, 123.3, 114.3 (2C), 78.8, 73.2, 70.7, 66.3, 54.9 (2C), 53.0, 51.8 (2C), 46.8, 26.6. HRMS (ESI_ACN). Calcd. for C_24_H_29_N_3_O_2_: 391.2259; found: 391.2265.

##### 3-(3-(4-(Prop-2-in-1-yl) piperazin-1-yl) propoxy)benzaldehyde (**25**)

Following general **method A**, the reaction of 3-hydroxybenzaldehyde (**14**) (107.3 mg, 0.88 mmol) with K_2_CO_3_ (365.4 mg, 2.65 mmol) and 1-(3-chloropropyl)-4-(prop-2-yn-1-yl)piperazine (**23**) (229.5 mg, 1.15 mmol) in CHCl_3_/H_2_O (5/1, 11 mL), after column chromatography (CH_2_Cl_2_/MeOH, 3%), afforded carbaldehyde **25** as an oil (283.5 mg, 86%): IR (film) ν 2935, 2812, 1692, 1259, 1149 cm^−1^; ^1^H NMR (400 MHz, CDCl_3_) δ 9.90 (s, 1H), 7.40–7.35 (m, 2H), 7.32 (dt, *J* = 2.4, 1.3 Hz, 1H), 7.10 (dt, *J* = 6.3, 2.4 Hz, 1H), 4.02 (t, *J* = 6.3 Hz, 2H), 3.24 (d, *J* = 2.4 Hz, 2H), 2.70–2.38 (m, 10H), 2.19 (t, *J* = 2.4 Hz, 1H), 2.03–1.85 (m, 2H); ^13^C NMR (101 MHz, CDCl_3_) δ 192.2, 159.6, 137.8, 130.0, 123.5, 122.0, 112.7, 78.7, 73.3, 66.5, 54.9, 53.0 (2C), 51.7, 46.8, 28.4 26.5. HRMS (ESI_ACN). Calcd. for C_17_H_22_N_2_O_2_: 286.1681; found: 286.1684.

##### (E)-*N*-Benzyl-1-(3-(3-(4-(prop-2-yn-1-yl) piperazin-1-yl) propoxy)phenyl)methanimine oxide xide (**9**)

Following general **Method B**, the reaction of compound **25** (120 mg, 0.42 mmol) with NaHCO_3_ (52.9 mg, 0.62 mmol), Na_2_SO_4_ (0.1193 mg, 0.84 mmol), and *N*-benzylhydroxylamine hydrochloride (100.5 mg, 0.63 mmol), in THF (4 mL) for 30 min, after column chromatography (MeOH/CH_2_Cl_2_, 8%), afforded nitrone **9** (110.6 mg, 56%): mp 98–101 °C; IR (KBr) ν 2933, 2834, 1593, 1265, 1179 cm^−1^; ^1^H NMR (500 MHz, CDCl_3_) δ 8.19 (s, 1H, H2), 7.52–7.36 (m, 6H), 7.31–7.25 (m, 1H), 7.04–6.90 (m, 1H), 5.05 (s, 2H), 4.16–3.98 (m, 2H), 3.30 (d, *J* = 2.5 Hz, 2H), 2.92–2.44 (m, 11H), 2.25 (t, *J* = 2.5 Hz, 1H), 2.03–1.91 (m, 2H); ^13^C NMR (126 MHz, CDCl_3_) δ 158.9, 134.3, 133.1, 131.5, 129.25, 129.22, 129.0 (4C), 121.6, 117.8, 113.0, 78.9, 73.1, 71.3, 66.3, 55.0, 53.0 (2C), 51.8 (2C), 46.8, 26.7. HRMS (ESI_ACN). Calcd for C_24_H_29_N_3_O_2_: 391,22598; found: 391.2266.

##### 2-(3-(4-(Prop-2-in-1-yl) piperazin-1-yl) propoxy)benzaldehyde (**26**)

Following general **method A**, the reaction of 2-hydroxybenzaldehyde (**15**) (93.9 mg, 0.769 mmol) with K_2_CO_3_ (318.5 mg, 2.3 mmol) and 1-(3-chloropropyl)-4-(prop-2-yn-1-yl)piperazine **(23)** (200 mg, 1 mmol) in CHCl_3_/H_2_O (5/1, 9 mL), after column chromatography (CH_2_Cl_2_/MeOH, 5%), gave carbaldehyde **26** (198.7 mg, 69%): mp 54–6 °C; IR (KBr) ν 2937, 2812, 1692, 1448, 1259, 1149 cm^−1^; ^1^H NMR (400 MHz, CDCl_3_) δ 10.43 (s, 1H), 7.76 (dd, *J* = 7.7, 1.8 Hz, 1H), 7.46 (ddd, *J* = 8.4, 7.7, 1.9 Hz, 1H), 7.02–6.87 (m, 2H), 4.08 (t, *J* = 6.3 Hz, 2H), 3.24 (d, *J* = 2.5 Hz, 2H), 2.76–2.36 (m, 10H), 2.18 (t, *J* = 2.5 Hz, 1H), 2.04–1.92 (m, 2H); ^13^C NMR (101 MHz, CDCl_3_) δ 189.7, 161.3, 135.9, 128.3, 124.9, 120.6, 112.5, 78.7, 73.2, 66.7, 54.9, 53.1 (2C), 51.7, 46.8 (2C), 26.5. HRMS (ESI_ACN). Calcd. for C_17_H_22_N_2_O_2_: 286.1681; found: 286.1685.

##### (Z)-*N*-Benzyl-1-(2-(3-(4-(prop-2-yn-1-yl) piperazin-1-yl)propyl) phenyl)methanimine oxide xide (**11**)

Following general **Method B**, the reaction of compound **26** (100 mg, 0.35 mmol) with NaHCO_3_ (44.50 mg, 0.53 mmol), Na_2_SO_4_ (99,4 mg, 0,70 mmol) and *N*-benzylhydroxylamine hydrochloride (84.6 mg, 0.53 mmol), in THF (4 mL), for 30 min, after column chromatography (MeOH/CH_2_Cl_2_, 8%), gave nitrone **11** (141 mg, 85%): mp 107–110 °C; IR (KBr) ν 2950, 2825, 1476, 1455, 1241 cm^−1^; ^1^H NMR (400 MHz, CDCl_3_) δ 9.19 (d, *J* = 7.7 Hz, 1H), 7.79 (s, 1H), 7.45–7.17 (m, 6H), 6.92 (t, *J* = 7.7 Hz, 1H), 6.79 (d, *J* = 8.4 Hz, 1H), 5.00 (s, 2H), 3.96 (t, *J* = 6.3 Hz, 2H), 3.25 (d, *J* = 2.6 Hz, 2H), 2.74–2.31 (m, 10H), 2.19 (t, *J* = 2.6 Hz, 1H), 1.88 (p, *J* = 6.6 Hz, 2H); ^13^C NMR (101 MHz, CDCl_3_) δ 156.3, 133.6, 131.5, 129.2 (2C), 128.94 (2C), 128.91, 128.8, 128.7, 120.7, 119.6, 110.7, 78.7, 73.2, 71.6, 66.5, 55.0, 53.1 (2C), 51.8 (2C), 46.8, 26.7. HRMS (ESI_ACN). Calcd. for C_24_H_29_N_3_O_2_: 391.2259; found: 391.2266.

##### (Z)-*N*-*tert*-Butyl-1-(4-(3-(4-(prop-2-yn-1-yl) piperazin-1-yl) propoxy) phenyl) methanimine oxide xide (**8**)

Following general **Method C**, the reaction of compound **24** (108.9 mg, 0.38 mmol) with AcONa (46.7 mg, 0.57 mmol), Na_2_SO_4_ (108 mg, 0.76 mmol), and *N*-*tert*-butylhydroxylamine hydrochloride (71.6 mg, 0.57 mmol), in EtOH (3 mL), for 12 h, after column chromatography (MeOH/CH_2_Cl_2_, 6%), afforded nitrone **8** (45.3 mg, 35%) as an oil: IR (film) ν 2931, 2823, 1505, 1600, 1246, 1120 cm^−1^; ^1^H NMR (400 MHz, CDCl_3_) δ 8.20 (d, *J* = 8.4 Hz, 2H), 7.39 (s, 1H), 6.85 (d, *J* = 8.4 Hz, 2H), 3.99 (t, *J* = 6.5 Hz, 2H), 3.23 (d, *J* = 3.0 Hz, 2H), 2.77–2.35 (m, 10H), 2.18 (t, *J* = 3.0 Hz, 1H), 1.92 (p, *J* = 7.0 Hz, 2H), 1.53 (s, 9H); ^13^C NMR (101 MHz, CDCl_3_) δ 160.2, 130.7 (2C), 129.5, 124.0, 114.3 (2C), 78.8, 73.2, 70.0, 66.2, 55.0, 53.0 (2C), 51.8 (2C), 46.8, 28.3 (3C), 26.6. HRMS (ESI_ACN). Calcd. for C_21_H_31_N_3_O_2_: 357.2416; found: 357.2416.

##### (Z)-*N*-*tert*-Butyl-1-(3-(3-(4-(prop-2-yn-1-yl) piperazin-1-yl) propoxy) phenyl) methanimine oxide xide (**10**)

Following general **Method C**, the reaction of compound **25** (158 mg, 0.85 mmol) with AcONa (104.96 mg, 1.28 mmol), Na_2_SO_4_ (241.4 mg, 1.7 mmol) and *N*-*tert*-butylhydroxylamine hydrochloride (160.8 mg, 1.28 mmol), in EtOH (8 mL) for 12 h, after column chromatography (MeOH/CH_2_Cl_2_, 4%), gave nitrone **10** (168.4 mg, 55%) as an oil: IR (film) ν 2933, 2812, 1578, 1243, 1183, 1153, 1120 cm^−1^; ^1^H NMR (400 MHz, CDCl_3_) δ 8.27 (d, *J* = 2.8 Hz, 1H), 7.46 (s, 1H), 7.39 (d, *J* = 7.7 Hz, 1H), 7.26–7.20 (m, 1H), 6.89 (dd, *J* = 8.2, 2.8 Hz, 1H), 4.01 (t, *J* = 6.4 Hz, 2H), 3.23 (d, *J* = 2.7 Hz, 2H), 2.72–2.36 (m, 10H), 2.18 (t, *J* = 2.5 Hz, 1H), 1.92 (p, *J* = 6.8 Hz, 2H), 1.54 (s, 9H); ^13^C NMR (101 MHz, CDCl_3_) δ 159.0, 132.2, 129.9, 129.1, 121.9, 117.6, 113.0, 78.8, 73.2, 70.9, 66.2, 55.1, 53.0 (2C), 51.8 (2C), 46.8, 28.36 (3C), 26.7. HRMS (ESI_ACN). Calcd. for C_21_H_31_N_3_O_2_: 357.2416; found: 357.2416.

##### (Z)-*N*-*tert*-Butyl-1-(2-(3-(4-(prop-2-yn-1-yl) piperazin-1-yl) propoxy) phenyl) methanimine oxide xide (**12**)

Following general **Method C**, the reaction of compound **26** (88 mg, 0.31 mmol) with AcONa (25.17 mg, 0.31 mmol), Na_2_SO_4_ (43.59 mg, 0.31 mmol) and *N*-*tert*-butylhydroxylamine hydrochloride (57.8 mg, 0.46 mmol), in EtOH (3 mL), for 12 h, after column chromatography (MeOH/CH_2_Cl_2_, 8%), afforded nitrone **12** (49.9 mg, 45%) as an oil: IR (film) ν 2926, 2819, 1595, 1457, 1237, 1125 cm^−1^; ^1^H NMR (500 MHz, CDCl_3_) δ 9.33 (dd, *J* = 7.9, 1.8 Hz, 1H), 8.06 (s, 1H), 7.33 (ddd, *J* = 8.2, 7.9, 1.8 Hz, 1H), 7.01 (td, *J* = 7.9, 0.9 Hz, 1H), 6.88 (dd, *J* = 8.2, 1.1 Hz, 1H), 4.07 (t, *J* = 6.2 Hz, 2H), 3.31 (d, *J* = 2.4 Hz, 2H), 2.70–2.41 (m, 10H), 2.25 (t, *J* = 2.4 Hz, 1H), 2.08–1.99 (m, 2H), 1.61 (s, 9H); ^13^C NMR (126 MHz, CDCl_3_) δ 156.4, 131.1, 128.6, 124.3, 120.7, 120.1, 110.6, 78.7, 73.2, 70.9, 66.5, 55.3, 53.2 (2C), 51.8 (2C), 46.8, 28.3 (3C), 26.8. HRMS (ESI_ACN). Calcd. for C_21_H_31_N_3_O_2_: 357.2416; found: 357.2418.

### 2.2. Estimation of Lipophilicity as Clog P

Lipophilicity is an important physicochemical property related to biological efficacy and ADME properties. Therefore, we used Bioloom from Biobyte Corp for the theoretical calculation of lipophilicity as Clog *P* values (BioByte home page, available online: http://www.biobyte.com (accessed 11 July 2021).

### 2.3. In Vitro Antioxidant Activity and Anti-Inflammatory Assays of Nitrones ***1***–***12*** and PBN

Several assays were used to evaluate in vitro antioxidant activity of nitrones, such as the inhibition of lipid peroxidation (LP), induced by AAPH in the presence of atmospheric oxygen, competition of the tested compounds with DMSO, in terms of hydroxyl radical scavenging activity, and ABTS^+∙^ decolorization assay. In vitro inhibition of soybean lipoxygenase (LOX) was used to determine anti-inflammatory activity. Reagents and materials: nordihydroguaiaretic acid (NDGA), Trolox, 2,2′-azobis(2-amidinopropane) dihydrochloride (AAPH), soybean LOX, and linoleic acid sodium salt were from Aldrich Chemical Co. Milwaukee, WI, (USA). Phosphate buffer (0.1 M, pH 7.4) was prepared by mixing an aqueous KH_2_PO_4_ solution (50 mL, 0.2 M), and an aqueous NaOH solution (78 mL, 0.1 M); pH (7.4) was adjusted by adding a solution of KH_2_PO_4_ or NaOH. A Lambda 20 (Perkin–Elmer-PharmaSpec 1700, Boston, MA, USA) UV–Vis double beam spectrophotometer was used for the assays.

#### 2.3.1. Inhibition of Linoleic Acid Peroxidation (ILPO)

A total of 10 μL of a 16 mM linoleate sodium solution and 0.93 mL of a 0.05 M phosphate buffer (pH 7.4), prethermostatted at 37 °C, were added to the UV cuvette. Then, 50 μL of a 40 mM AAPH solution was added and used as a free radical initiator at 37 °C in presence of air [18]. Finally, 10 μL of the tested compounds were added. The oxidation of linoleic acid sodium salt results in a conjugated diene hydroperoxide. The reaction was monitored by measuring the absorbance at 234 nm. Trolox was used as a reference compound and positive control.

#### 2.3.2. In Vitro Inhibition of Soybean LOX

The in vitro study was performed as previously described [25]. The compounds were incubated at room temperature with sodium linoleate (0.1 mM) and 0.2 mL of soybean lipoxygenase solution (1/9 × 10^–4^ *w*/*v* in phosphate buffer saline). The method was based on the conversion of sodium linoleate to 13-hydroperoxylinoleic acid, which was detected by measuring absorbance at 234 nm. NDGA was used as a positive control. To determine the IC_50_ values, serial dilutions of compounds were used. Blank determination served as a negative control.

#### 2.3.3. Competition of the Tested Compounds with DMSO for Hydroxyl Radicals

Hydroxyl radicals were generated by the Fe^3+^/ascorbic acid system and detected by the determination of formaldehyde generated by the oxidation of DMSO. EDTA (0.1 mM), Fe^3+^ (167 μM), and DMSO (33 mM) in phosphate buffer (50 mM, pH 7.4); the tested compounds (0.1 mM) and ascorbic acid (10 mM) were mixed in test tubes and incubated at 37 °C for 30 min. [25] The reaction was stopped by adding CCl_3_COOH (17% *w*/*v*), and the percentage (%) scavenging activity of the tested compounds for hydroxyl radicals was calculated. Trolox was used as a positive control.

#### 2.3.4. ABTS^+^–Decolorization Assay in Ethanolic Solution for Antioxidant Activity

In order to produce the ABTS radical cation (ABTS^+·^), ABTS stock solution in water (7 mM) was mixed with potassium persulfate (2.45 mM) and left in the dark at room temperature for 12–16 h before use. The assay was performed as previously described [25]. Absorbances of the mixed solution were measured after 1 min at 734 nm. Trolox was used as a positive control.

#### 2.3.5. DPPH Radical-Scavenging Assay

The assay was performed as previously described [26] by incubating compounds at a concentration of 100 µM in the presence of 2,2-diphenyl-1-picrylhydrazyl radical (DPPH, 70 µM) in methanol for 90 min, protected from sun light. The absorbance at 517 nm was then determined using a Synergy™ H4 microplate reader (BioTek Instruments, Inc., Winooski, VT, USA). The experiments were performed in triplicate, and the blank (compound without DPPH) was substracted. The percentage of DPPH free radicals was calculated using the equation DPPH free radicals (%) = ((*A*_0_ − *A*_1_)/*A*_0_) × 100, where *A_0_* is the absorbance of MeOH and *A*_1_ is the absorbance of the compound. Trolox and resveratrol were used as positive controls.

### 2.4. Inhibition of Cholinesterases and Monoamine Oxidases

The inhibitory potencies of the compounds towards ChEs were determined following the procedure described previously [26]. Recombinant ChEs, namely hAChE and hBChE, were kindly provided by Dr. Xavier Brazzolloto and Dr. Florian Nachon (IRBA, Brétigny sur Orge, France). The assay was performed in sodium phosphate buffer (0.100 M, pH = 8.0), containing 370 µM 5,5′-dithiobis (2-nitrobenzoic acid), 500 µM substrates (acetylthiocholine/butyrylthiocholine iodide for hAChE and hBChE, respectively), and hAChE or hBChE (50 pM and 1 nM, respectively). The reactions were started by adding the substrate. The final concentration of DMSO was always 1% (*v*/*v*). Change of the absorbance at 412 nm was monitored for 2 min using a microplate reader (Synergy™ H4, BioTek Instruments, USA). For inhibitory screening, compounds were assayed at 100 µM in triplicate. The initial velocities in the presence (*v_i_*) and absence (*v*_0_) of the compounds were calculated, and the inhibitory potencies were expressed as residual activities (RAs): RA (%) = *v_i_* / *v*_0_ × 100. For IC_50_ determination, a serial dilution of the compounds was prepared. IC_50_ values were calculated using the 4-parameter logistic function via GraphPad Prism 9.3 software (GraphPad Software, San Diego, CA, USA). Tacrine and donepezil were used as positive controls.

The effects of the compounds on hMAO-A/B were investigated using a fluorimetric assay following a method previously described in the literature [26]. Recombinant hMAO expressed in BTI-TN-5B1-4 baculovirus infected insect cells, *p*-tyramine hydrochloride, horseradish peroxidase (HRP, type II) were from Sigma Aldrich. Briefly, 100 µL of 50 mM potassium phosphate buffer (pH = 7.4, 0.05% (*v*/*v*) Triton X-114), containing the compounds at a concentration of 100 µM and hMAO, was incubated at 37 °C for 15 min. The reaction was started by adding 200 µM Amplex Red (200 µM, final concentration), 1 U/mL HRP, and 1 mM *p*-tyramine (200 µL, final volume). The increase in fluorescence emission (*λ*_ex_  =  530 nm, *λ*_em_  =  590 nm) was monitored over 20 min period using a microplate reader (Synergy™ H4, BioTek Instruments, USA). As for control, DMSO replaced the compound solution, and phosphate-buffered solution was used instead of enzyme to determine the *b* (blank) value. Initial velocities were calculated from the trends obtained, with each measurement carried out in duplicate. The inhibitory potencies were expressed as RAs (%) = (*v_i_* − *b*)/ (*v*_0_ − *b*) × 100. Harmine and safinamide were used as positive controls.

### 2.5. Inhibition or Aβ_1–42_ Aggregation

Thioflavin-T (ThT) assay [27] was performed to investigate the effect of the compounds on the aggregation of Aβ_1–42_ (HFIP-pretreated Aβ_1–42_ peptide Merck Millipore, Darmstadt, Germany). Prior to the incubations, 1 mM Aβ_1–42_ peptide stock solution in DMSO was diluted in 150 mM HEPES buffer (pH = 7.4, 150 mM NaCl) to obtain a concentration of 7.5 μM. Subsequently, 20 µL of Aβ_1–42_ (10 µM, final concentration) was mixed with the compounds (10 µM, final concentration) on black-walled 96-well plates and diluted with ThT solution (14.3 μM stock solution in HEPES-buffered solution; 10 μM final concentration). The final volume of the mixture was 100 μL, and the DMSO concentration was always 3% (*v*/*v*). Each sample was prepared in four technical replicates. ThT fluorescence (*λ*_ex_  =  440 nm, *λ*_em_  =  490 nm) was measured using a microplate reader (Synergy™ H4, BioTek Instruments, Inc., Winooski, VT, USA) for 36–48 h. Inhibition of Aβ_1–42_ aggregation was expressed as the percent inhibition (% inh = (1 − *F_i_*/*F*_0_) × 100), where *F_i_* is the increase in fluorescence of compound-treated Aβ_1–42_, and *F*_0_ is the increase in fluorescence of non-treated Aβ_1–42_.

### 2.6. Molecular Docking of Inhibitor 5 into hBChE

Nitrone **5** in protonated form was assembled using Discovery Studio software, version 2.1, using default settings. Using the CHARMm force field [28] and partial atomic charges, the molecular geometry of nitrone **5** was energy-minimized using the adopted-based Newton–Raphson algorithm. The coordinates of hBChE in complex with tacrine (PDB ID: 4BDS) were taken from the Protein Data Bank (PDB). Proper bonds, bond orders, hybridization, and charges were assigned using the protein model tool of Discovery Studio software, version 2.1. AutoDockTools (ADT; version 1.5.6) was used to add hydrogens and partial charges for proteins and ligands using Gasteiger charges, as well as to convert both to pdbqt format. Docking calculations were performed with the program Autodock Vina as “blind dockings”, where a grid box of size 66 × 60 × 74 Å, with grid points separated by 1 Å, was positioned at the center of the protein (x = 136.0; y = 123.59; z = 38.56). Default parameters were used, except for num_modes, which was set to 40. The conformation with the lowest docking-energy was considered the most stable orientation. Finally, the generated docking results were loaded directly into Discovery Studio Visualizer 2021.

### 2.7. Neuroprotection Experiments

The MTT reduction method was selected to evaluate the neuroprotective profile of selected compounds in an in vitro neurodegeneration model [29]. Briefly, the SH-SY5Y neuroblastoma cell culture was maintained and seeded, as previously described [30]. After 24 h of cell culturing, the cells were treated with **1**, **5**, or **6** at the concentrations indicated in Figure 5 for an additional 24 h. Then, the medium was replaced with a fresh one containing FBS 1%, compounds, and OA at a final concentration of 20 nM. The AD drug memantine, at a concentration of 30 nM, was used as a standard. Twenty hours later, cell viability was measured by the MTT method. For this purpose, yellow-colored MTT at a final concentration of 1.2 mM was added to the wells, where it was reduced to purple, and water-insoluble formazan by mitochondrial dehydrogenases of viable cells. After removal of the medium, the purple formazan was dissolved with 0.3 mL of DMSO, and the absorbance of each well was measured in a spectrophotometric reader at 540 nm.

## 3. Results and Discussion

### 3.1. Chemistry

**Scheme 1:**
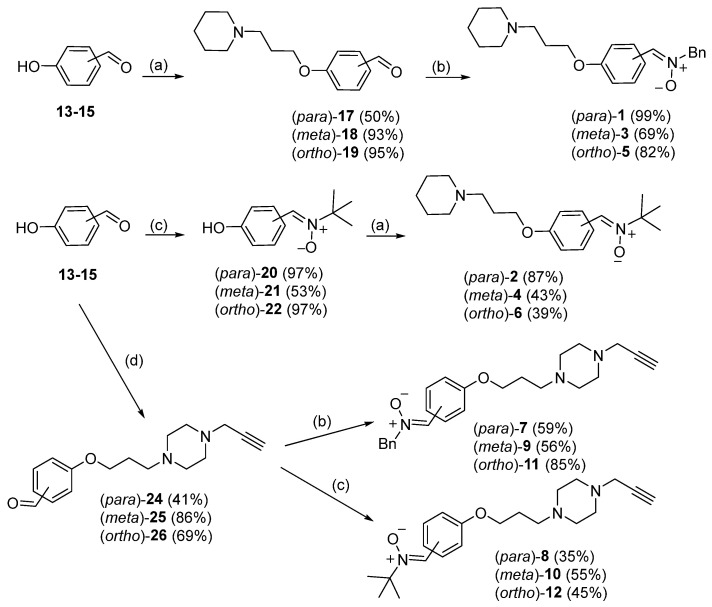
Synthesis of **PBN**-derived polyfunctionalized nitrones **1**–**12**: (a) 1-(3-chloropropyl) piperidine (**16**), K_2_CO_3_, CHCl_3_/H_2_O, 80 °C; (b) BnNHOH.HCl, Na_2_SO_4_, NaHCO_3_, THF, 80 °C; (c) *tert*-BuNHOH.HCl, Na_2_SO_4_, AcONa, EtOH, MWI, 90 °C, 15 bar; (d) 1-(3-chloropropyl)-4-(prop-2-yn-1-yl) piperazine (**23**), K_2_CO_3_, CHCl_3_/H_2_O, 80 °C.

The synthesis of **PBN**-nitrones **1**–**12** was carried out as shown in Scheme 1, starting from hydroxybenzaldehydes **13**–**15**. *O*-alkylation of the precursors with 1-(3-chloropropyl) piperidine **16** yielded intermediates **17**–**19** [21], which were further reacted with *N*-benzylhydroxylamine hydrochloride to afford **PBN**-piperidinepropoxy nitrones **1**, **3** and **5** in good overall yields (Scheme 1). To increase the yields of **PBN**-piperidinepropoxy nitrones **4–6** synthesis, the reaction sequence was reversed; i.e., *N-tert*-butyl nitrones **20**–**21 [22]** were *O*-alkylated with 1-(3-chloropropyl) piperidine (**16**) (Scheme 1). Hydroxybenzaldehydes **13**–**15** were reacted with 1-(3-chloropropyl)-4-(prop-2-yn-1-yl) piperazine (**23**) [20], followed by reaction with the corresponding *N*-alkylhydroxylamine hydrochloride to furnish **PBN**-functionalized nitrones **7**–**12** (Scheme 1).

All nitrones were isolated as *Z* isomers [20] and yielded analytical and spectroscopic data in good agreement with their structures (see Experimental Part and Supplementary Material).

### 3.2. Antioxidant Assays

The in vitro bioactivity of **PBN** and nitrones **1**–**12** was evaluated in a diverse range of antioxidant assays, using nordihydroguaiaretic acid (NDGA) and Trolox as controls [25]. Free radicals generated during the biochemical function of all aerobic organisms, are a highly reactive species that can damage biological molecules, i.e., lipids, DNA, and proteins, and subsequently cause neurodegenerative diseases, cancer, and stroke. Different approaches are used to determine the antioxidant capacity of compounds to obtain results related to different mechanisms of action. Factors such as solubility and steric hindrance, which may be of paramount importance in one environment but not in another, are varied, and the antioxidant properties of compounds are evaluated in different environments.

AAPH is a free radical-generating, water-soluble azo compound that generates peroxyl radicals in solution and induces oxidation of linoleic acid at room temperature and a constant and reproducible rate, without generating hydrogen peroxide. The method measures how effectively antioxidants can inhibit lipid peroxidation in vitro. As shown in Table 1, only nitrone **4**, which combines the *tert*-butyl and piperidinepropyloxy moieties, exhibits potent anti-lipid peroxidation activity (45%), although this is lower, compared to Trolox (93%). The group of compounds exhibiting low antioxidant activity includs nitrones **10**, **12** (31%), and **3** (29%). The common structural feature of active nitrones is the presence of a *tert*-butyl moiety as R^1^ (Table 1). On the contrary, when a benzyl group is introduced, the inhibition of lipid peroxidation is lower. With respect to **PBN**, the absence of -OR substituents leads to decreased activity (11%), compared to nitrone **4**. *Meta*-substitution in the series of **PBN**-piperidinepropyloxy, as well as *N*-propargyl piperazinepropyloxy (e.g., nitrone **10**) nitrones, leads to improved antioxidant properties in the ABTS assay.

Among the ROS, the hydroxyl free radical (^•^OH) is the most reactive and toxic, reacting with all biologically important molecules, such as DNA, lipids, or carbohydrates. Therefore, we determined the competition of nitrones **1**–**12** with DMSO for the hydroxyl radicals to investigate their ^•^OH scavenging ability. Of the two series, the *N*-propargylpiperazinepropyloxy-substituted nitrones are more potent, with nitrone **9** being the most potent, followed by nitrone **11,** both of which are Bn substituted analogues. As observed in the ABTS assay, the *meta*-substituted derivatives are more potent in comparison to *ortho-* and *para*-substituted counterparts. Moreover, the *tert*-Bu substituted *p*-*N*-propargylpiperazinepropyloxy derivative strongly competes with DMSO for hydroxyl radicals. Of the piperidinepropyloxy derivatives, the *meta*- and *para*-substituted *tert*-butyl nitrones **2** and **4**, respectively, are equipotent hydroxyl radical scavengers.

Among the nitrones tested, the *o-tert*-butyl substituted piperidinepropoxy derivative **5** (10 μM) is the most potent lipoxygenase (LOX) inhibitor, whereas nitrones **1** (60 μM) and **9** (100 μM) have IC_50_ values in the same range (Table 1).

No significant results were taken from the scavenging experiment of the nitrones with the cationic radical ABTS^+^ (13–31%). The highest activity (31%) was shown by the **3**, the Bn-substituted *N*-propargyl piperazinepropoxy derivative. As shown in Table 1, the **PBN**-analogues were inactive in an orthogonal 2,2-diphenyl-1-picrylhydrazyl (DPPH) assay.

From the analysis of antioxidant activities, we concluded that nitrone **5** can be considered the most potent LOX inhibitor, which might result in anti-inflammatory effect in cellulo and in vivo.

To evaluate the capacity of **PBN**-analogues **1**–**12** (Figure 1) to act as multifunctional ligands, we screened the compounds on human MAOs (hMAO-A, hMAO-B) and ChEs, namely acetylcholinesterase (hAChE) and butyrylcholinestrase (hBChE) [26]. As shown in Table 2, the nitrones do not inhibit hMAO-A/hMAO-B and hAChE, whereas **PBN**-analogues **1**, **3**, **4**, **10**, and **11** (Figure 1, Table 2) inhibit hBChE, with IC_50_ values ranging from 16 to 67 µM. **PBN**-analogues **5** and **6** were the most potent and selective hBChE inhibitors, with IC_50_ values of 3.46 ± 0.27 µM and 6.52 ± 1.12 µM, respectively. These results prompted us to perform modelling docking simulations using AutoDock Vina to propose the binding mode of nitrone **5** to hBChE [31]. The crystal structure of hBChE, bound with tacrine, was taken from the RCSB Protein Data Bank (PDB ID: 4BDS).

### 3.3. Additional Pharmacological Evaluation

**Figure 2:**
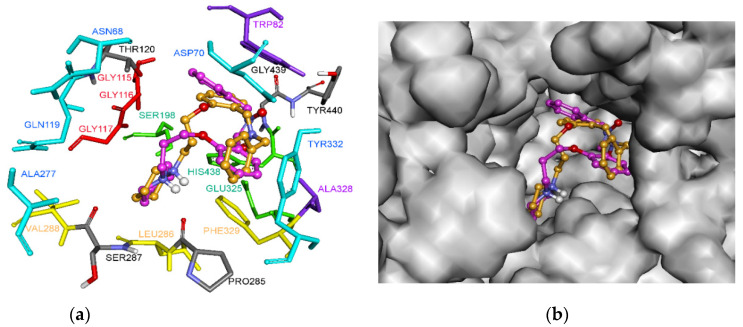
Proposed binding modes for nitrone 5 in the active site of hBChE. Nitrone **5** in mode I, the carbon atoms are shown in pink (binding energy: –9.4 kcal/mol). Nitrone **5** in mode II is shown in orange (binding energy: –9.2 kcal/mol). (**a**) The different subsites of the active site are colored as follows: catalytic Ser198 and His438 in green, oxyanion hole in red, CBS in violet, ABP in yellow, and PAS in blue; (**b**) Outer surface of the hBChE active site, with both binding poses of nitrone **5**.

**Figure 3:**
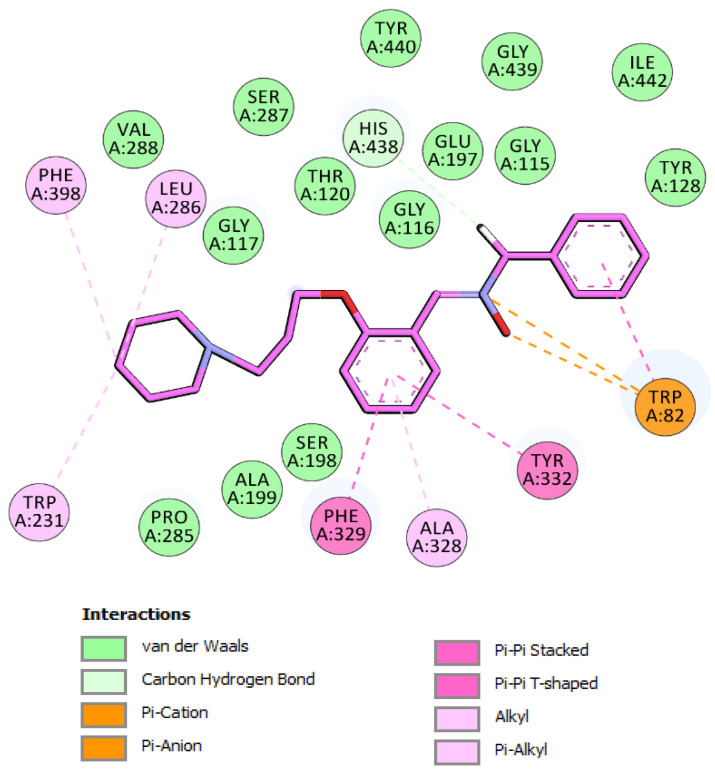
Schematic 2D representation of hBChE binding site amino acids interacting with nitrone **5**. Binding mode I.

**Figure 4:**
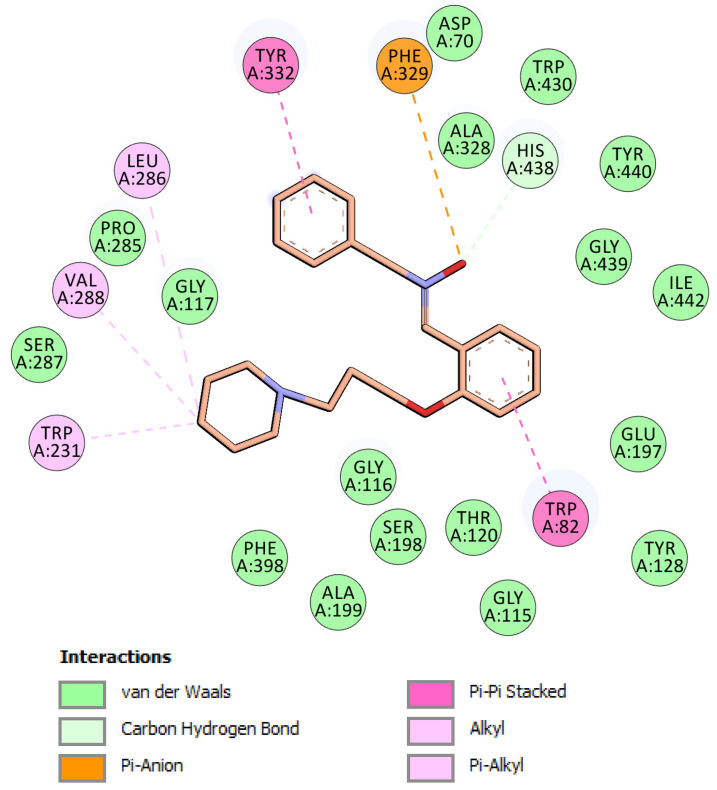
Schematic 2D representation of hBChE binding site amino acids interacting with nitrone **5**. Binding mode II.

Docking results show that nitrone **5** has two main predicted binding modes, modes I and II (Figure 2). In both binding modes, the three rings occupied the same regions inside the active site cavity, where piperidines were spatially overlapped and pointed towards the catalytic amino acid residues Ser198 and His438. However, binding modes I and II differed in the relative orientation of benzyl and phenoxy motifs. In mode I, the position of the phenoxy motif overlapped with the benzyl group in mode II and vice versa. Despite the reversed orientation, both rings interacted with the same key amino acid residues (Figure 3 and Figure 4).

The analysis of binding mode I (Figure 3), the most energetically favorable mode, highlighted dual-binding interactions with both the catalytic triad (i.e., the catalytic anionic site and CAS) and peripheral anionic site (PAS). The phenyl, nitrone, and phenoxy moieties interacted with the choline binding site (CBS) through π-π, π-cation, and π-anion interactions with Trp82, as well as with Ala328 through van der Waals interactions. In this binding mode, the methylene of the benzyl group pointed to the catalytic residues and showed a carbon hydrogen interaction with His438. In addition, the phenoxy core interacted with Tyr332 of PAS by π-π T-shaped interaction, and with Phe329 on the acyl binding pocket (ABP) through a π-π stacked interaction. Moreover, piperidine completely occupied the ABP and formed π-alkyl and alkyl interactions with Trp231 and Leu286. In binding mode I, nitrone **5** is located near Tyr128, Ile442, Gly115, Gly116, Gly117, Gly439, Tyr440, Glu197, Thr120, Ser287, Ser198, Ala199, Pro285, and Val288 to form favorable van der Waals interactions (Figure 3).

In mode II (Figure 4), the phenoxy moiety of **5** was oriented towards the bottom of the active site and binds the CAS region of the enzyme, establishing π-π and van der Waals interactions with two key amino acids of the CBS, namely Trp82 and Ala328. In addition, the π-anion interaction was established between Phe329 (ABP) and the oxygen atom of the nitrone moiety. This oxygen also interacted with His438 via carbon hydrogen bonding, which is presumably important for hBChE inhibition. Residues Trp231, Val288, and Leu286 further stabilized the position of piperidine in the active site via π-alkyl and alkyl interactions (Figure 4). The *N*-benzyl nitrone **5** interacted with PAS, where phenyl ring formed π-π stacked interactions with Tyr332 and van der Waals interactions with Asp70.

Next, the absorption, distribution, metabolism, and elimination (ADME) properties for nitrone **5** were predicted using the QikProp module of Schrodinger suite (Schrödinger release 2021-4: QikProp, Schrödinger, LLC, New York, NY, 2021) to assess the drug-like properties (Table 3). Pharmacologically relevant properties and descriptors of nitrone **5** were predicted and some of the crucial properties are highlighted here. According to Lipinski’s rule of five (ROF) [32], in general, a drug candidate has to show no more than one violation of the following criteria: (1) no more than five hydrogen bond donors (donorHB), (2) no more than 10 hydrogen bond acceptors (accptHB), (3) a molecular weight (MW) lower than 500 D, and (4) an octanol–water partition coefficient (logPo/w) no greater than 5. As shown in Table 3, the partition coefficient (QPlogPo/w) for nitrone **5** is slightly above 5, and this is the only violation of the Lipinski’s ROF. The drug-likeness of nitrone **5** also follows the Jorgensen’s rule of three (ROT) [33]: QPlogS > −5.7, QPCaco > 22 nm/s and number of primary metabolites < 7. The solubility of organic molecules in water has a significant impact on several ADME-related properties. This compound presented a solubility (QPlogS) value that is within the limits (−6.5–0.5 mol/dm^3^). The BBB (blood–brain barrier) permeability is another important parameter that affects the in vivo biological activity. Drugs that target the central nervous system must cross the BBB to achieve the desired pharmacological effect. The hydrophilicity (logS) and log BB are the principle descriptors of CNS penetration. Log BB is a hybrid parameter determined by permeability, plasma, and brain tissue binding, as well as an active transport mechanism, with a desired range from −3.0 to +1.2. The log BB value, calculated for nitrone **5,** was in the required range, which proposed sufficient crossing of BBB. Caco-2 cell permeability is an indication of intestinal absorption of drugs; in line with our results, nitrone **5** shows great permeation (1388.113 nm/sec). Orally active drugs that are transported by the transcellular route should not exceed the PSA of about 120 Å^2^. For good brain penetration of CNS drugs, this number should even be tailored to PSA < 100 Å^2^ or even lower, i.e., <60–70 Å^2^. The value of PSA for nitrone **5** is 32.545 Å^2^, which, again, meant good penetration of BBB. Other physicochemical descriptors, calculated using QikProp (Table 3), were within the desired value ranges; thus, nitrone **5** can be regarded as an antioxidant with suitable drug-like properties.

Finally, the capacity of the nitrones to inhibit amyloid β (Aβ_1–42_) aggregation was assessed in ThT assay, as described previously [27]. The majority of compounds did not inhibit aggregation of Aβ_1–42_ under the conditions used, only nitrone **1** (Figure 1) inhibited Aβ_1–42_ aggregation (42.2 ± 5.7%) at the 10 µM compound and 1.5 µM Aβ_1–42_.

**Figure 5:**
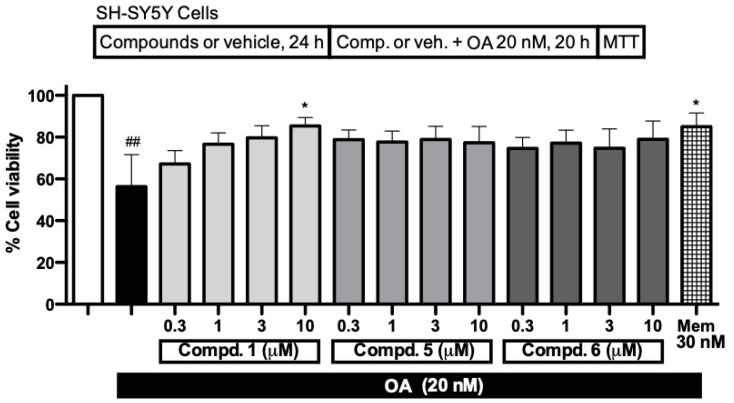
Cell viability (in %) of SH-SY5Y neuroblastoma cells challenged with okadaic acid (OA), and neuroprotective effect of compounds 1, 5 and 6 (0.3–10 µM). Cell viability of cells subjected to OA (20 nM) for 20 h was measured using MTT assay, and compared with cell viability of vehicle treated cells (white bar). Data are mean ± SEM of four independent experiments. ^##^ *p* < 0.01, with respect to untreated cells (white bar); * *p* < 0.05, with respect to SH-SY5Y cells only treated with OA (black bar).

Based on the results of the in vitro assays, described above, the neuroprotective activity of nitrones **1**, **5**, and **6** was investigated. SH-SY5Y neuroblastoma cells were first pre-incubated with nitrones, and then exposed to the toxic stimuli, i.e., okadaic acid (20 nM) for 20 h (Figure 5). In vitro exposure of SH-SY5Y cells to okadaic acid is a widely used in vitro model for neuronal damage [34]. Okadaic acid inhibits Ser/Thr phosphatases, e.g., PP2A, a major regulatory enzyme of the balance between phosphorylation and dephosphorylation, resulting in changes, e.g., excitotoxicity and oxidative stress, which, in turn, affected cell viability. Increased oxidative stress is related to the involvement of PP2A-mediated activity in the modulation of phosphoproteins that trigger inflammatory and stress signalling [35]. Nitrones **1**, **5**, and **6** augmented the OA-reduced cell viability (Figure 5), with **1** showing a clear dose-response effect, being statistically significant at 10 µM. Compounds **5** and **6** showed a sustained but slight protecting profile, elevating cell viability by about 50%, compared to the OA-treated cells that did not reach statistical significance. The AD drug memantine at a concentration of 30 nM was used as standard in these experiments [36]. Thus, we appreciated that the determined antioxidant properties of nitrones **1**, **5**, and **6** turned into a moderate neuroprotective feature under the OA model of neuronal damage.

## 4. Conclusions

To sum up, in this work, we report the synthesis, antioxidant, and neuroprotective properties of twelve polyfunctionalized α-phenyl-*tert*-butyl(benzyl) nitrones. The antioxidant capacity of the nitrones was studied by different assays, including the inhibition of lipid peroxidation induced by AAPH, hydroxyl radical scavenging assay, ABTS^+∙^–decolorization assay, DPPH scavenging assay, and inhibition of soybean lipoxygenase. Monoamine oxidase and cholinesterase inhibitory potencies, as well as the inhibition of the amyloid β aggregation, were also determined. A comprehensive evaluation revealed that nitrone **5** ((*Z*)-*N*-benzyl-1-(2-(3-(piperidin-1-yl)propoxy)phenyl)methanimine oxide) is one of the most potent antioxidants, with high ABTS^+∙^ scavenging activity (19%) and potent lipoxygenase (IC_50_ = 10 µM), as well as butyrylcholinesterase (IC_50_ = 3.46 ± 0.27 µM) inhibition with slight neuroprotective activity in OA-induced cellular model of neuronal damage. Overall, these results pave the way for further in-depth neuroprotection analysis of nitrone **5** in in vitro and in vivo models of stroke and possibly other neurodegenerative diseases in which the oxidative stress is identified as a critical player.

## Figures and Tables

**Table 1:** Antioxidant activity of **PBN**-polyfunctionalized nitrones **1**–**12**.

Nitrones/Standard	ClogP *^a^*	ILPO *^b^* (%)	LOX Inhibition (% *^b^* or IC_50_ [μM])	OH Scav. Capacity (%) *^b^*	ABTS^+^ (%) *^b^*	DPPH *^b^*
**PBN**	3.02	11	23%	no	5	no
**1**	3.75	**n.a.**	**60 μM**	**7**	**27**	**0**
**2**	3.22	n.a.	n.a.	54	n.a.	0
**3**	3.75	**29**	**n.a.**	**n.a.**	**31**	**2**
**4**	3.22	**45**	**37%**	**54**	**23**	**0**
**5**	3.68	**n.a.**	**10 μM**	**9**	**19**	**0**
**6**	3.15	**n.a.**	**39%**	**13**	**n.a.**	**0**
**7**	2.90	n.a.	27.2%	n.a.	28	0
**8**	2.37	n.a.	23.3%	78	10	0
**9**	2.90	n.a.	100 μM	81	n.a.	1
**10**	2.37	**31**	**no**	**20**	**13**	**0**
**11**	2.83	**6**	**38%**	**71**	**15**	**0**
**12**	2.30	31	25%	9.8	13	o
**NDGA**	3.92	n.d.	0.45 μM	n.d.	n.d.	n.d.
**Trolox**	3.09	93	n.d.	88	91	n.d.

*^a^* Biobyte Corporation, C-QSAR database, 201 W Fourth Str., Suite # 204, Claremont CA 91711-4707, USA. *^b^* Compounds assayed at 100 µM. Values are means of three or four different determinations. n.a.: not active. n.d.: not determined. no: no activity under the experimental conditions.

**Table 2:** In vitro ChE and MAO inhibitory potencies of nitrones **1**–**12**.

Nitrones	hMAO-A	hMAO-B	hAChE	hBChE
	IC_50_ [µM] ± SEM *^a^*			
**1**	n.a. *^b^*	n.a. *^b^*	n.a. *^b^*	45.82 ± 0.96
**2**	n.a. *^b^*	n.a. *^b^*	n.a. *^b^*	n.a. *^b^*
**3**	n.a. *^b^*	n.a. *^b^*	n.a. *^b^*	16.35 ± 1.03
**4**	n.a. *^b^*	n.a. *^b^*	n.a. *^b^*	28.87 ± 3.41
**5**	n.a. *^b^*	n.a. *^b^*	n.a. *^b^*	**3.46 ± 0.27**
**6**	n.a. *^b^*	n.a. *^b^*	n.a. *^b^*	**6.52 ± 1.12**
**7**	n.a. *^b^*	n.a. *^b^*	n.a. *^b^*	n.a. *^b^*
**8**	n.a. *^b^*	n.a. *^b^*	n.a. *^b^*	n.a. *^b^*
**9**	n.a. *^b^*	n.a. *^b^*	n.a. *^b^*	n.a. *^b^*
**10**	n.a. *^b^*	n.a. *^b^*	n.a. *^b^*	51.04 ± 2.21
**11**	n.a. *^b^*	n.a. *^b^*	n.a. *^b^*	67.30 ± 6.48
**12**	n.a. *^b^*	n.a. *^b^*	n.a. *^b^*	n.a. *^b^*

*^a^* IC_50_ values are expressed as mean ± SEM of three independent experiments. *^b^* n.a., not active (residual activity—RA at 100 µM > 50%).

**Table 3:** Qikprop calculated physicochemical properties for nitrone **5**.

**QPPCaco**	**PSA**	**QPlogBB**	**metab**	**QPlogKhsa**	**% HOA**	**ROF**	**ROT**
1388.113	32.545	0.131	3	0.919	100.000	1	0
**MW**	**SASA**	**volume**	**donorHB**	**accptHB**	**QPlogPo/w**	**QPlogS**	**MW**
352.475	725.561	1269.781	0.000	3.750	5.441	–5.318	352.475

**MW**: molecular weight of the molecule (130.0–725.0). **SASA**: total solvent-accessible surface area, in square angstroms, using a probe with a 1.4 Å radius (limits 300.0–1000.0). **Volume**: total solvent-accessible volume, in cubic angstroms, using a probe with a 1.4 Å radius (limits 500.0–2000.0). **DonorHB**: estimated number of hydrogen bonds that would be accepted by the solute (limits: 2.0–20.0). **AccptHB**: estimated number of hydrogen bonds that would be donated by the solute (limits: 0.0–6.0). **QPlogPo/w**: predicted octanol/water partition coefficient (limits −2.0–6.5). **QPlogS**: predicted aqueous solubility. S, in mol/dm^3^, is the concentration of the solute saturated solution that is in equilibrium with crystalline solid (limits −6.5–0.5). **QPPCaco**: predicted apparent Caco-2 cell permeability in nm/sec. Caco-2 cells are a model for the gut-blood barrier. QikProp predictions are for non-active transport (<25 poor, >500 great). **PSA**: van der Waals surface area of polar nitrogen and oxygen atoms (limits 7.0–200.0). **QPlog BB**: predicted brain/blood partition coefficient (limits −3.0–1.2). **Metab**: number of likely metabolic reactions (limits 1–8). **QPlogKhsa**: prediction of binding to human serum albumin (limits −1.5–1.5). **HOA**: predicted qualitative human oral absorption on 0 to 100% scale. **ROF**: number of violations of Lipinski’s rule of five. **ROT**: number of violations of Jorgensen’s rule of three.

## Data Availability

No application.

## Supplementary Materials

The following supporting information can be downloaded at: https://www.mdpi.com/article/10.3390/antiox11091735/s1, NMR and HRMS spectra of nitrones **1**–**12**.

## Abbreviations

AAPH: 2,2′-azobis(2-amidinopropane) dihydrochloride; (h)AChE, (human) acetylcholinesterase; AD, Alzheimer’s disease; BBB, blood−brain barrier; (h)BChE, (human) butyrylcholinesterase; ChEs, cholinesterase(s); CNS, central nervous system; HRP, horse-radish peroxidase; LOX, lipoxygenase; LP, lipid peroxidation; MAO, monoamine oxidase; MTT, 3-(4,5-dimethylthiazol-2-yl)-2,5-diphenyltetrazolium bromide; NDGA, nordihydroguaiaretic acid; OA, okadaic acid; PD, Parkinson’s disease; ROS, reactive oxygen species; ThT, thioflavin-T.
