# Supplementary material for: Polyfunctionalized α-Phenyl-tert-butyl(benzyl)nitrones: Multifunctional Antioxidants for Stroke Treatment
Source: Antioxidants (Basel). 2022 Aug 31;11(9):1735. doi: 10.3390/antiox11091735 (PMC9495348; doi:10.3390/antiox11091735)
Supplement: Supplementary file 1 [file antioxidants-11-01735-s001.zip › antioxidants-1883242-supplementary.pdf]

Supplementary Material

Polyfunctionalized α-Phenyl-tert-butyl(benzyl)nitrones: Multifunctional Antioxidants for Stroke Treatment

Daniel Diez-Iriepa,[1,2] Damijan Knez,[3] Stanislav Gobec,[3] Isabel Iriepa,[2,4] Cristóbal de los Ríos,[5,6] Isaac Bravo,[5,6] Francisco López-Muñoz,[7,8] José Marco-Contelles[1,9*] and Dimitra Hadjipavlou-Litina[10,*]

[1] Laboratory of Medicinal Chemistry, Institute of Organic Chemistry (CSIC), Juan de la Cierva 3, 28006-Madrid, Spain
[2] Universidad de Alcalá, Departamento de Química Orgánica y Química Inorgánica, Ctra. Madrid-Barcelona Km 33.6, 28871 Alcalá de Henares, Madrid, España
[3] University of Ljubljana, Faculty of Pharmacy, 1000 Ljubljana, Slovenia
[4] Institute of Chemical Research Andrés M. del Río, Alcalá University, 28805-Alcalá de Henares, Madrid, Spain
[5] Institute of Health Research, Hospital Universitario de la Princesa, 28006 Madrid, Spain
[6] Department of Pharmacology, Autonomous University of Madrid, 28034, Madrid, Spain
[7] Faculty of Health, Camilo José Cela University of Madrid (UCJC), Spain
[8] Neuropsychopharmacology Unit, "Hospital 12 de Octubre" Research Institute, Madrid, Spain
[9] Center for Biomedical Network Research on Rare Diseases (CIBERER), CIBER, ISCIII, Madrid, Spain
[10] Department of Pharmaceutical Chemistry, School of Pharmacy, Faculty of Health Sciences, Aristotle University of Thessaloniki, Thessaloniki 54124, Greece

Daniel Diez-Iriepa: daniel.diezi@uah.es; Damijan Knez: damijan.knez@ffa.uni-lj.si; Stanislav Gobec: stanislav.gobec@ffa.uni-lj.si; Isabel Iriepa: isabel.iriepa@uah.es; Cristóbal de los Ríos: cristobal.delosrios@inv.uam.es; Isaac Bravo: isaac.bravo@estudiante.uam.es; Francisco López-Muñoz: flopez@ucjc.edu; José Marco-Contelles: jlmarco@iqog.csic.es;

* Correspondence: * D.H.L.: hadjipav@pharm.auth.gr; Tel.: +302310997627; José Marco-Contelles: jlmarco@iqog.csic.es

Content

NMR and HRMS of compounds 17-19, 20-22, 24-26,

and nitrones 1-12……………………………………………………………………..S2-S64

Compound 17

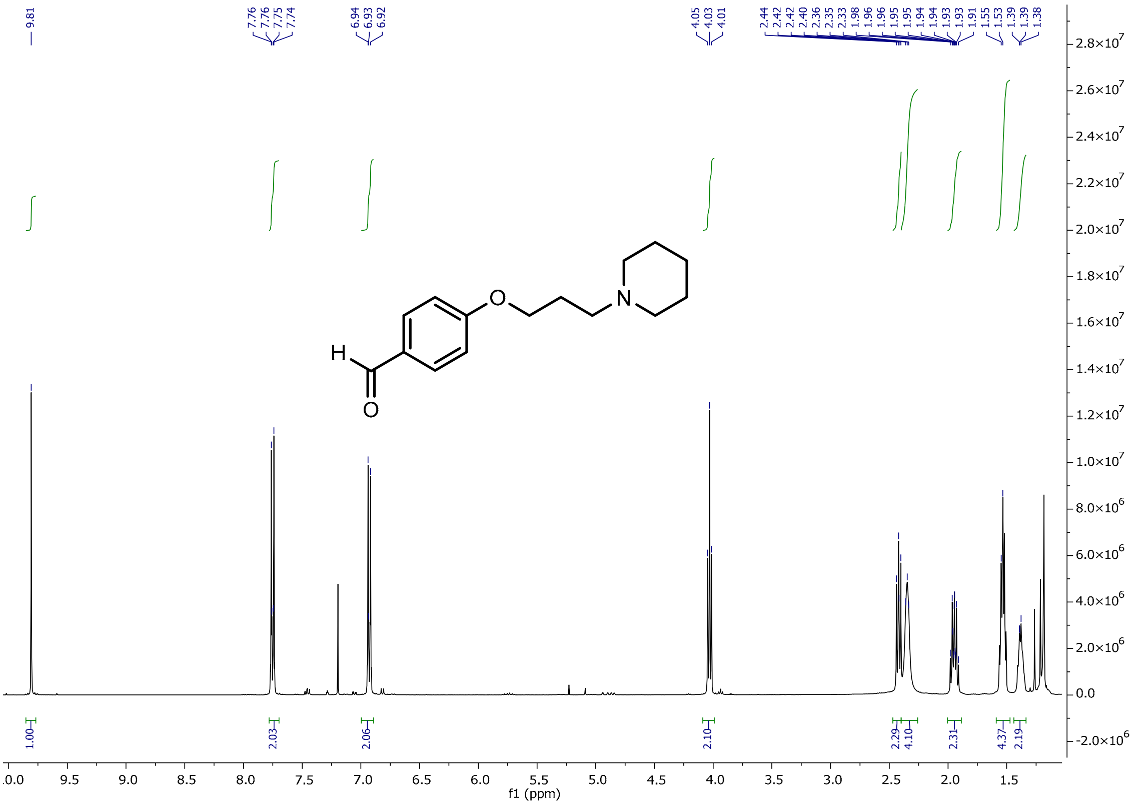
9.81
7.76 7.76 7.75 7.74
6.94 6.93 6.92
4.05 4.03 4.01
2.44 2.42 2.42 2.40 2.36 2.35 2.33 1.98 1.96 1.96 1.95 1.95 1.94 1.94 1.93 1.93 1.91 1.55 1.53 1.39 1.39 1.38
H
O
O
N
1.00
2.03
2.06
2.10
2.29
4.10
2.31
4.37
2.19
f1 (ppm)

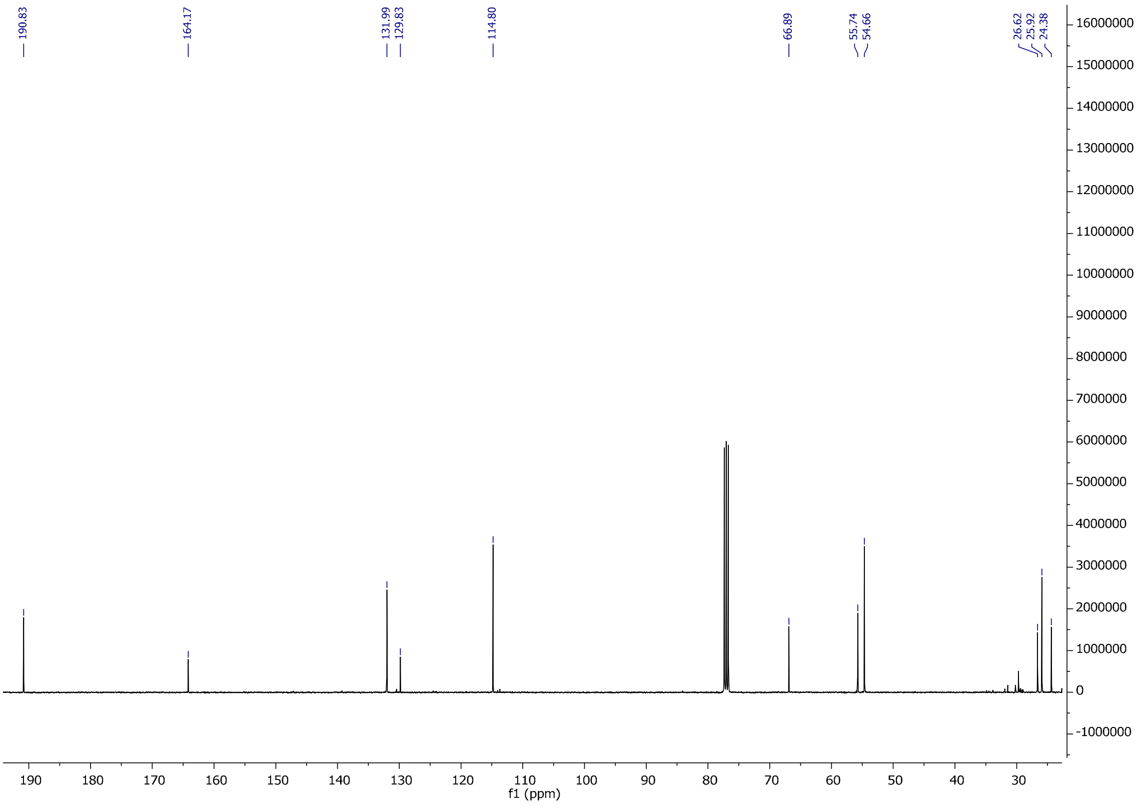
190.83
164.17
131.99
129.83
114.80
66.89
55.74
54.66
26.62
25.92
24.38
f1 (ppm)

Qualitative Compound Report

**Data File**	1526_DDI122_01.d	**Sample Name**	DDI122
**Sample Type**	Sample	**Position**	Vial 14
**Instrument Name**	Instrument 1	**User Name**	
**Acq Method**	ESI_ACN_75_pos_new.m	**Acquired Time**	6/9/2022 2:28:27 PM (UTC+02:00)
**IRM Calibration Status**	Success	**DA Method**	Defecto_modificado.m
**Comment**			

**Sample Group**		**Info.**	
**User**	MIREIA TOLEDANO	**Stream Name**	LC 1
**Acquisition Time (Local)**	6/9/2022 2:28:27 PM (UTC+02:00)	**Acquisition SW Version**	6200 series TOF/6500 series Q-TOF B.08.00 (B8058.3 SP1)
**QTOF Driver Version**	8.00.00	**QTOF Firmware Version**	2.712
**Tune Mass Range Max.**	3200		

Compound Table

Compound Label	RT	Mass	Abund	Formula	Tgt Mass	Diff (ppm)	Hits (DB)
Cpd 1: C15 H21 N O2; 0.336	0.336	247.1571	1997773	C15 H21 N O2	247.1572	-0.46	1

Compound Label	*m/z*	RT	Algorithm	Mass
Cpd 1: C15 H21 N O2; 0.336	248.1642	0.336	Find by Formula	247.1571

MS Zoomed Spectrum

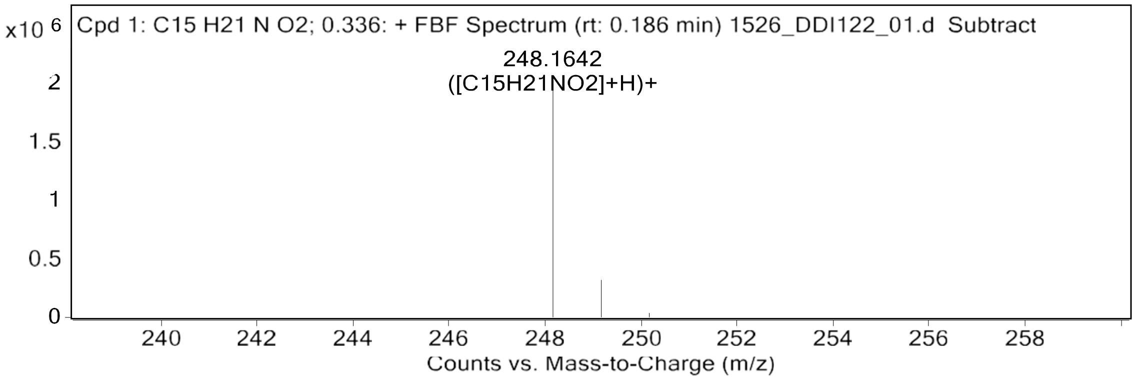

MS Spectrum Peak List

m/z	z	Abund	Formula	Ion
248.1642	1	1997773	C15H21NO2	(M+H)+
249.1689	1	318535.75	C15H21NO2	(M+H)+
250.1719	1	35877.32	C15H21NO2	(M+H)+

MS Zoomed Spectrum

Qualitative Compound Report

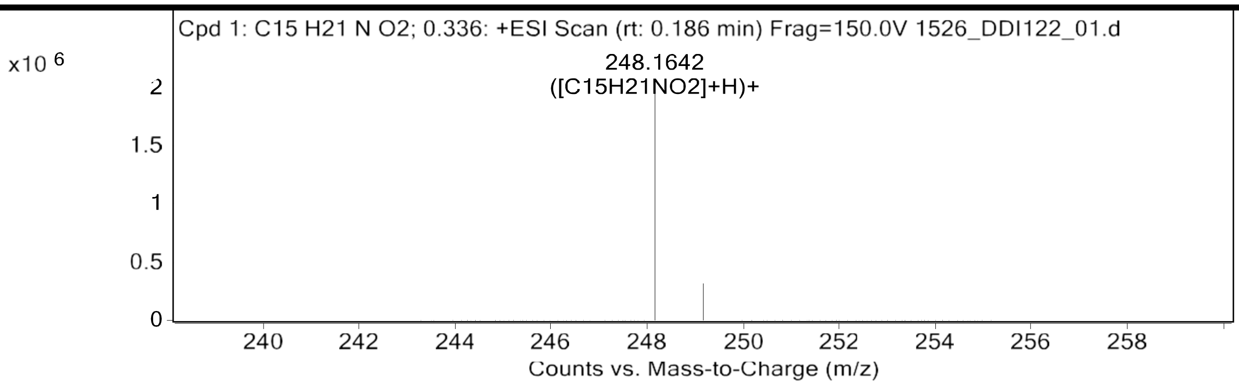

MS Spectrum Peak List

m/z	*Calc m/z*	Diff(ppm)	z	Abund	Formula	Ion
248.1642	248.1645	-1.38	1	1997773	C15H21NO2	(M+H)+
249.1689	249.1678	4.61	1	318535.75	C15H21NO2	(M+H)+
250.1719	250.1705	5.59	1	35877.32	C15H21NO2	(M+H)+

--- End Of Report ---

Nitrone 1

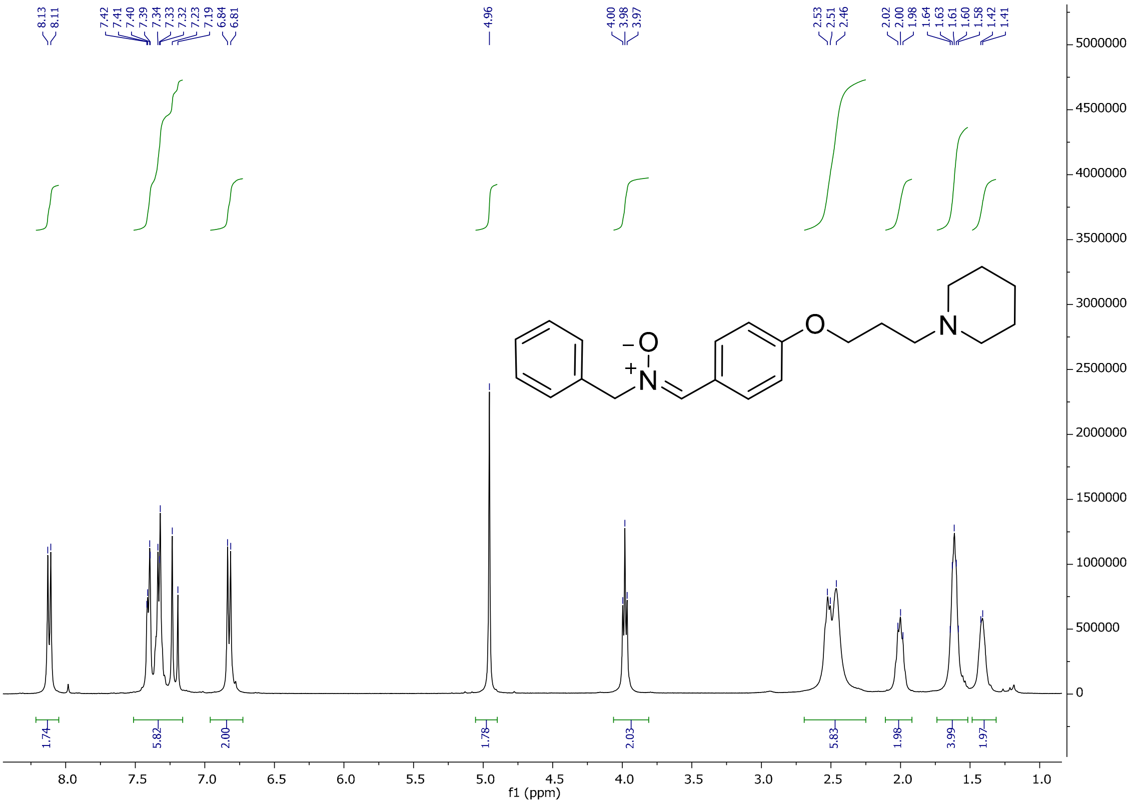

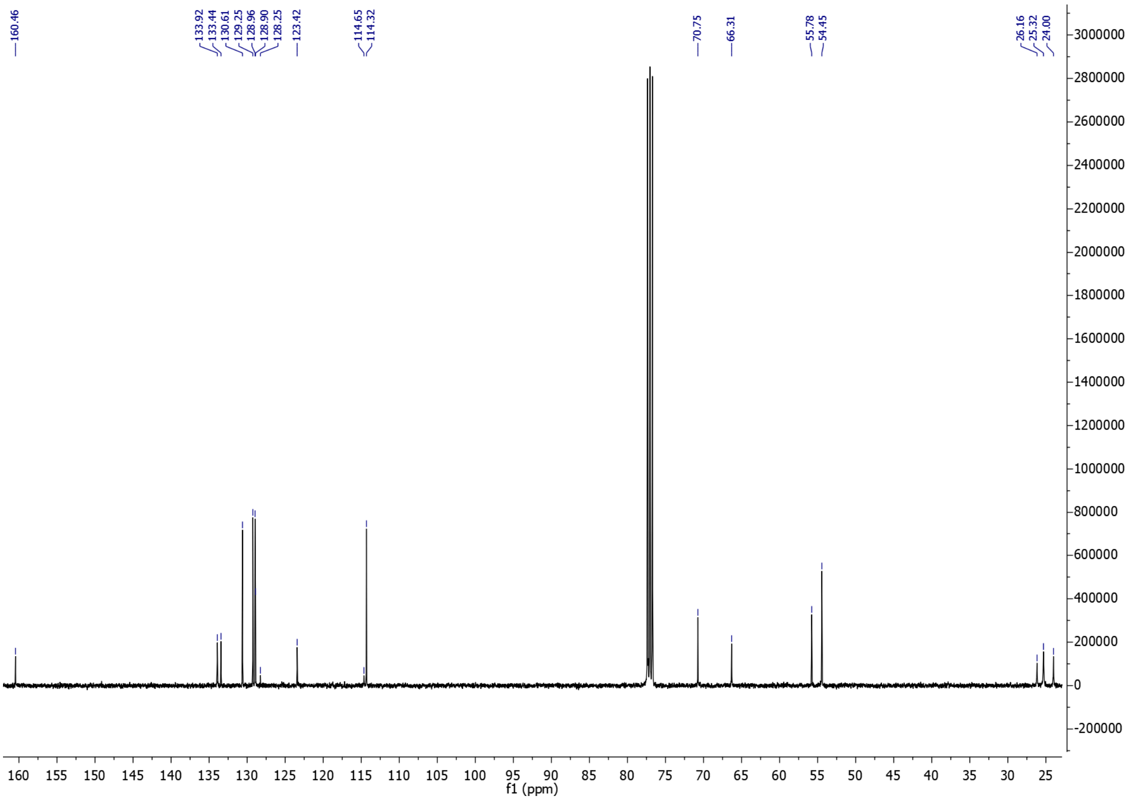

Qualitative Compound Report

**Data File**	250_DDI125_01.d	**Sample Name**	DDI125
**Sample Type**	Sample	**Position**	Vial 4
**Instrument Name**	Instrument 1	**User Name**	
**Acq Method**	ESI_ACN_75_pos_new.m	**Acquired Time**	2/19/2021 11:21:38 AM (UTC+01:00)
**IRM Calibration Status**	Some Ions Missed	**DA Method**	Defecto_modificado.m
**Comment**			

**Sample Group**		**Info.**	
**User**	DANIEL DIEZ	**Stream Name**	LC 1
**Acquisition Time (Local)**	2/19/2021 11:21:38 AM (UTC+01:00)	**Acquisition SW Version**	6200 series TOF/6500 series Q-TOF B.08.00 (B8058.3 SP1)
**QTOF Driver Version**	8.00.00	**QTOF Firmware Version**	2.712
**Tune Mass Range Max.**	1700		

Compound Table

Compound Label	RT	Mass	Abund	Formula	Tgt Mass	Diff (ppm)	Hits (DB)
Cpd 1: C22 H28 N2 O2; 2.710	2.71	352.2168	423509	C22 H28 N2 O2	352.2151	4.85	1

Compound Label	*m/z*	RT	Algorithm	Mass
Cpd 1: C22 H28 N2 O2; 2.710	353.2243	2.71	Find by Formula	352.2168

MS Zoomed Spectrum

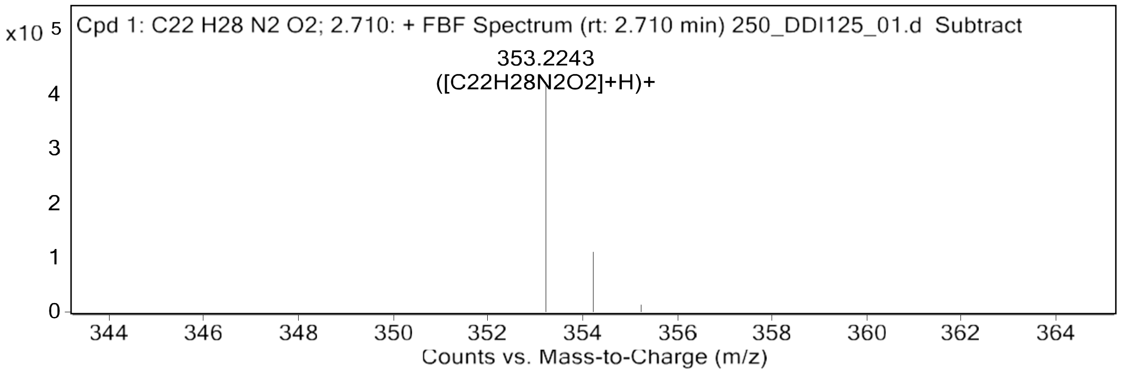

MS Spectrum Peak List

m/z	z	Abund	Formula	Ion
353.2243	1	423508.72	C22H28N2O2	(M+H)+
354.2257	1	110889.88	C22H28N2O2	(M+H)+
355.2354	1	13569.22	C22H28N2O2	(M+H)+

MS Zoomed Spectrum

Qualitative Compound Report

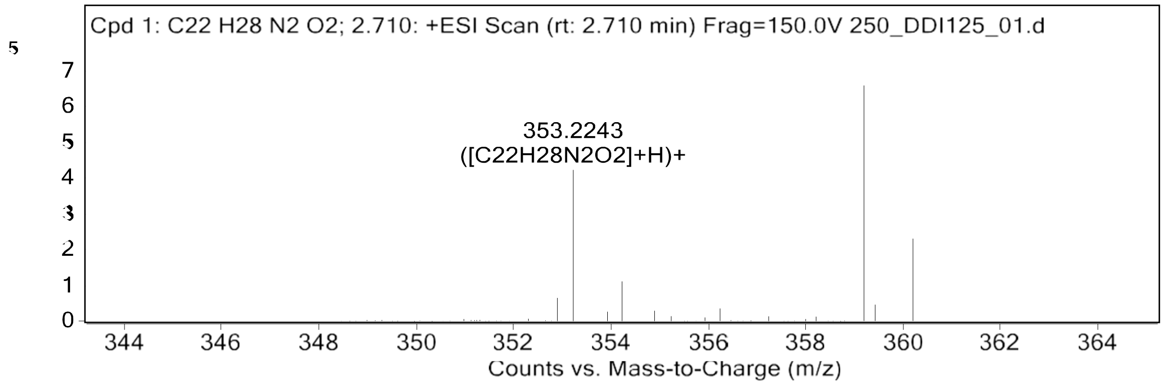

MS Spectrum Peak List

m/z	*Calc m/z*	Diff(ppm)	z	Abund	Formula	Ion
353.2243	353.2224	5.56	1	423508.72	C22H28N2O2	(M+H)+
354.2257	354.2256	0.28	1	110889.88	C22H28N2O2	(M+H)+
355.2354	355.2285	19.36	1	13569.22	C22H28N2O2	(M+H)+

--- End Of Report ---

Compound 18

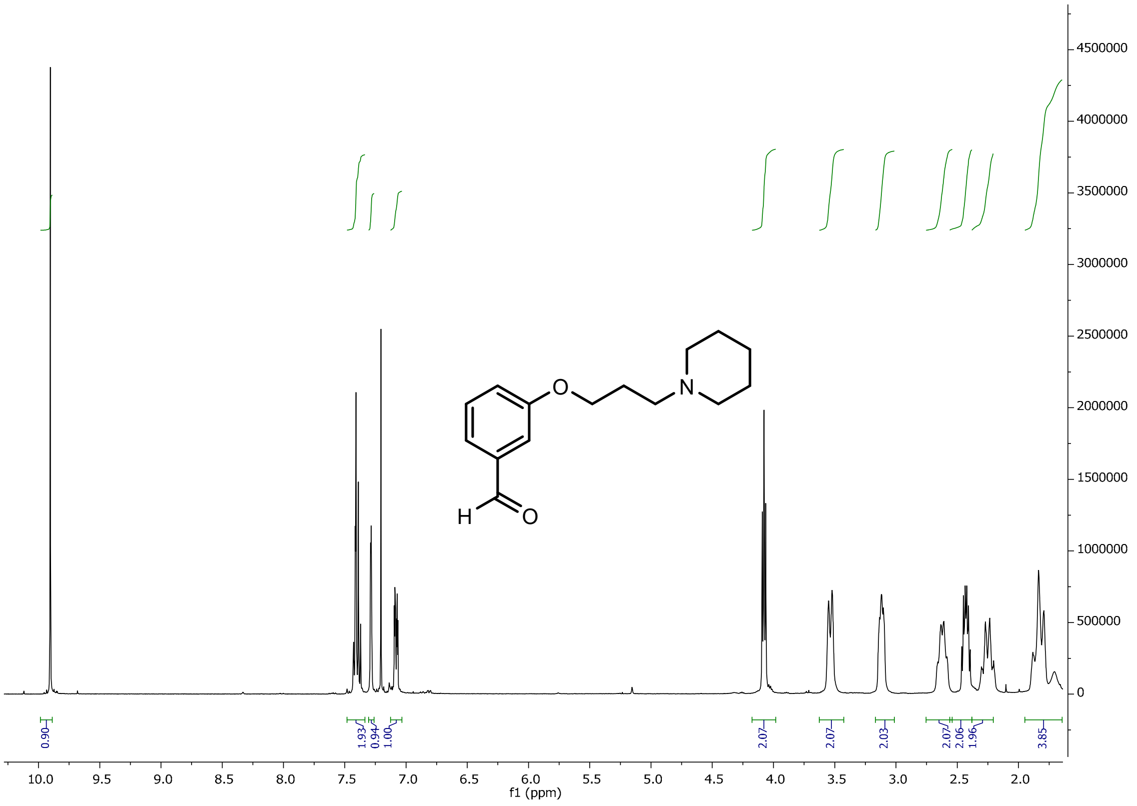

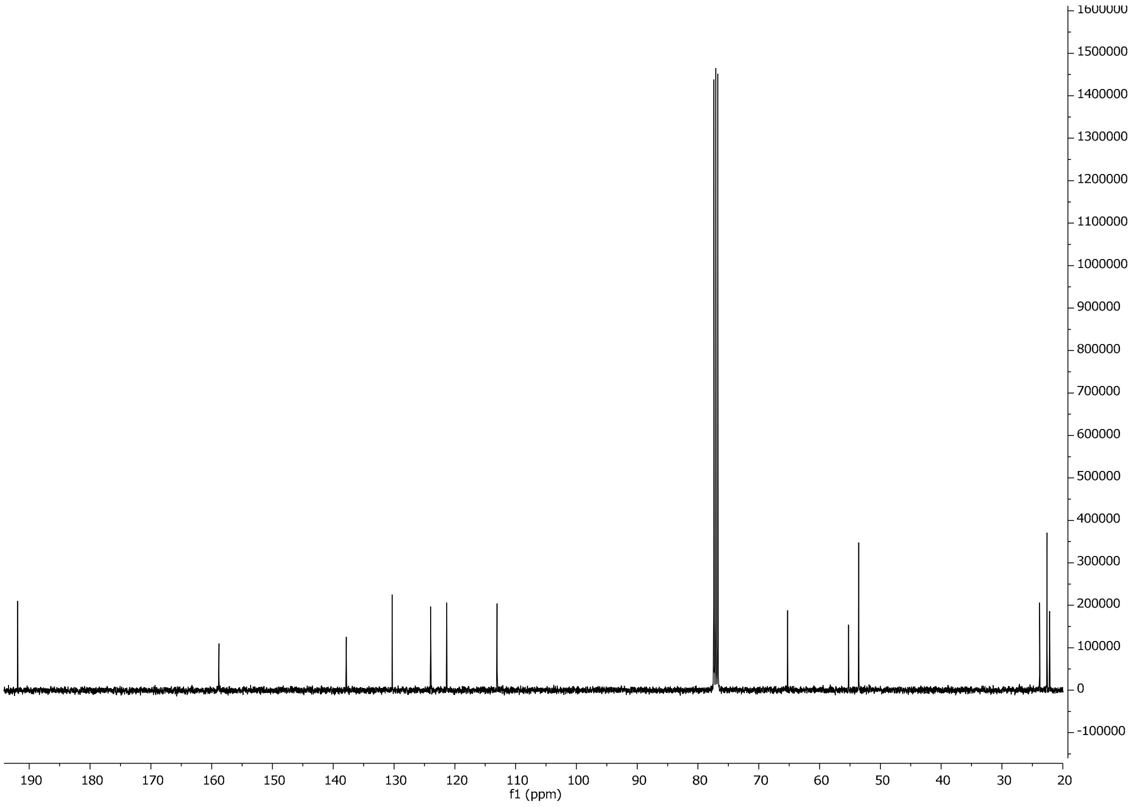

Qualitative Compound Report

**Data File**	1527_DDI127_01.d	**Sample Name**	DDI127
**Sample Type**	Sample	**Position**	Vial 15
**Instrument Name**	Instrument 1	**User Name**	
**Acq Method**	ESI_ACN_75_pos_new.m	**Acquired Time**	6/9/2022 2:35:45 PM (UTC+02:00)
**IRM Calibration Status**	Success	**DA Method**	Defecto_modificado.m
**Comment**			

**Sample Group**		**Info.**	
**User**	MIREIA TOLEDANO	**Stream Name**	LC 1
**Acquisition Time (Local)**	6/9/2022 2:35:45 PM (UTC+02:00)	**Acquisition SW Version**	6200 series TOF/6500 series Q-TOF B.08.00 (B8058.3 SP1)
**QTOF Driver Version**	8.00.00	**QTOF Firmware Version**	2.712
**Tune Mass Range Max.**	3200		

Compound Table

Compound Label	RT	Mass	Abund	Formula	Tgt Mass	Diff (ppm)	Hits (DB)
Cpd 1: C15 H21 N O2; 2.272	2.272	247.1574	1089957	C15 H21 N O2	247.1572	0.63	1

Compound Label	*m/z*	RT	Algorithm	Mass
Cpd 1: C15 H21 N O2; 2.272	248.1646	2.272	Find by Formula	247.1574

MS Zoomed Spectrum

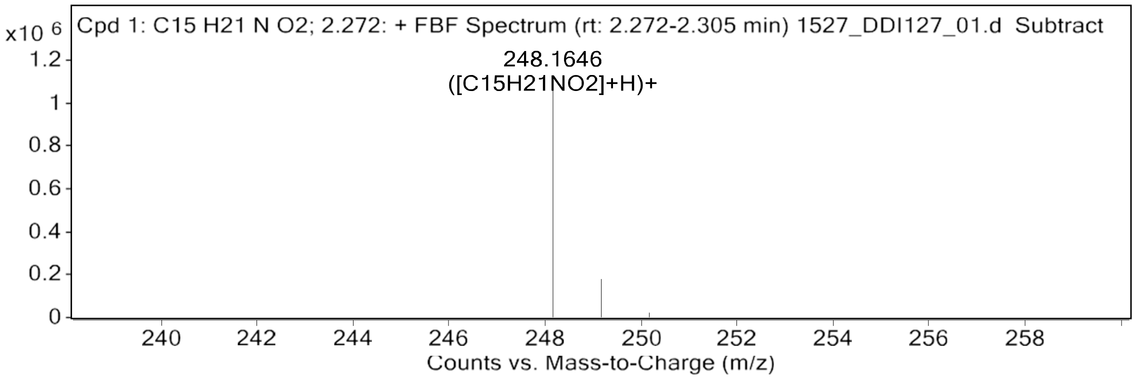

MS Spectrum Peak List

m/z	z	Abund	Formula	Ion
248.1646	1	1089956.63	C15H21NO2	(M+H)+
249.1681	1	178285.8	C15H21NO2	(M+H)+
250.1709	1	21668.81	C15H21NO2	(M+H)+

MS Zoomed Spectrum

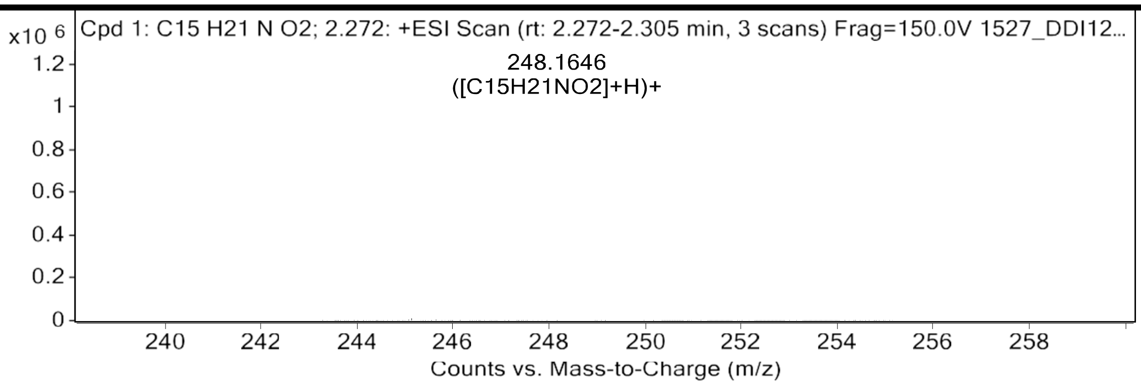

MS Spectrum Peak List

m/z	*Calc m/z*	Diff(ppm)	z	Abund	Formula	Ion
248.1646	248.1645	0.48	1	1089956.63	C15H21NO2	(M+H)+
249.1681	249.1678	1.43	1	178285.8	C15H21NO2	(M+H)+
250.1709	250.1705	1.7	1	21668.81	C15H21NO2	(M+H)+

--- End Of Report ---

Nitrone 3

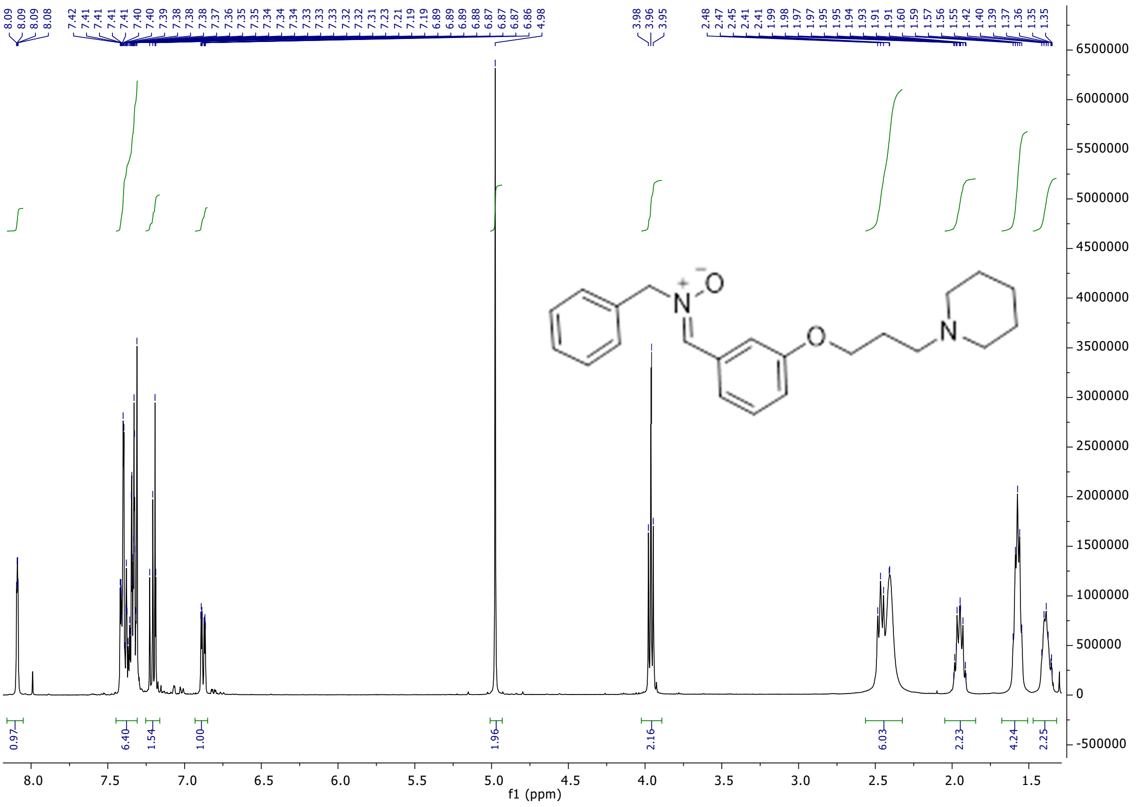

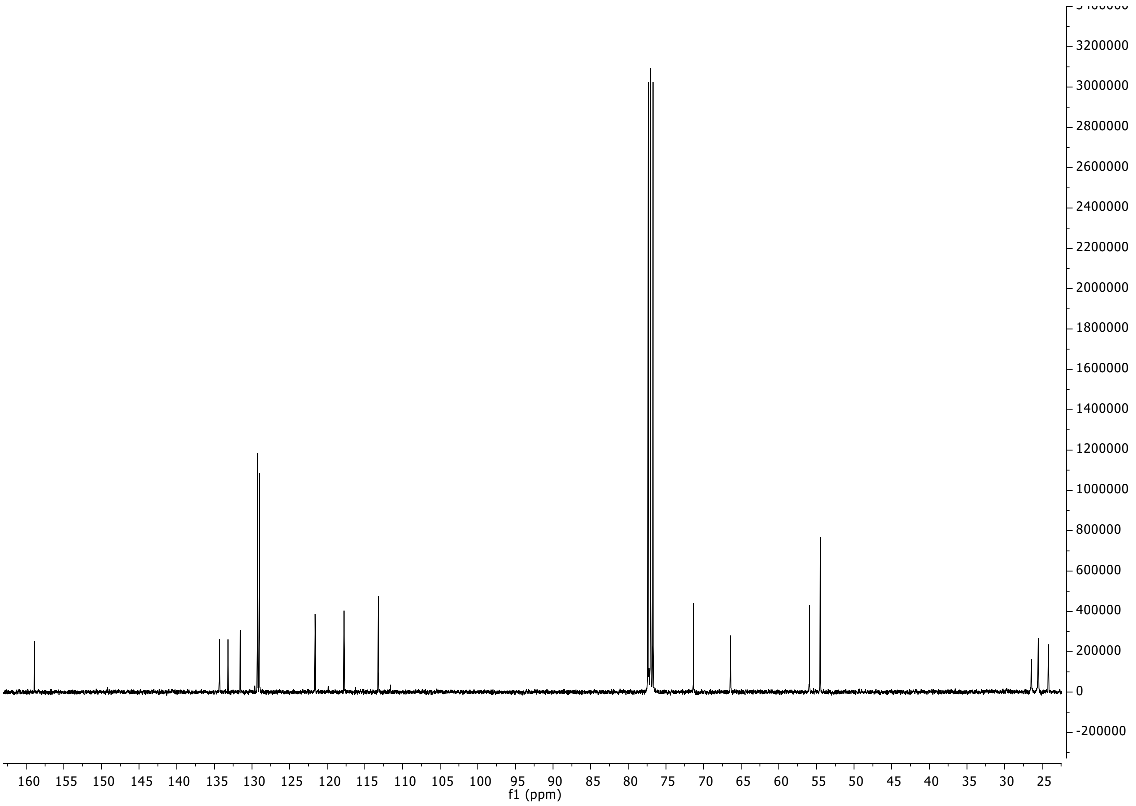

Qualitative Compound Report

**Data File**	251_DDI128_01.d	**Sample Name**	DDI128
**Sample Type**	Sample	**Position**	Vial 5
**Instrument Name**	Instrument 1	**User Name**	
**Acq Method**	ESI_ACN_75_pos_new.m	**Acquired Time**	2/19/2021 11:28:56 AM (UTC+01:00)
**IRM Calibration Status**	Some Ions Missed	**DA Method**	Defecto_modificado.m
**Comment**			

**Sample Group**		**Info.**	
**User**	DANIEL DIEZ	**Stream Name**	LC 1
**Acquisition Time (Local)**	2/19/2021 11:28:56 AM (UTC+01:00)	**Acquisition SW Version**	6200 series TOF/6500 series Q-TOF B.08.00 (B8058.3 SP1)
**QTOF Driver Version**	8.00.00	**QTOF Firmware Version**	2.712
**Tune Mass Range Max.**	1700		

Compound Table

Compound Label	RT	Mass	Abund	Formula	Tgt Mass	Diff (ppm)	Hits (DB)
Cpd 1: C22 H28 N2 O2; 1.219	1.219	352.2153	14037230	C22 H28 N2 O2	352.2151	0.5	1

Compound Label	*m/z*	RT	Algorithm	Mass
Cpd 1: C22 H28 N2 O2; 1.219	353.2225	1.219	Find by Formula	352.2153

MS Zoomed Spectrum

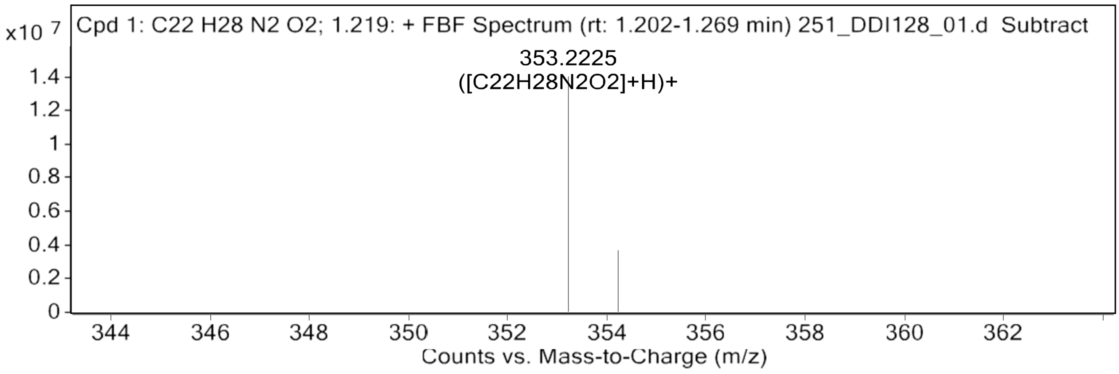

MS Spectrum Peak List

m/z	z	Abund	Formula	Ion
353.2225	1	14037230	C22H28N2O2	(M+H)+
354.2258	1	3672781.25	C22H28N2O2	(M+H)+

MS Zoomed Spectrum

Qualitative Compound Report

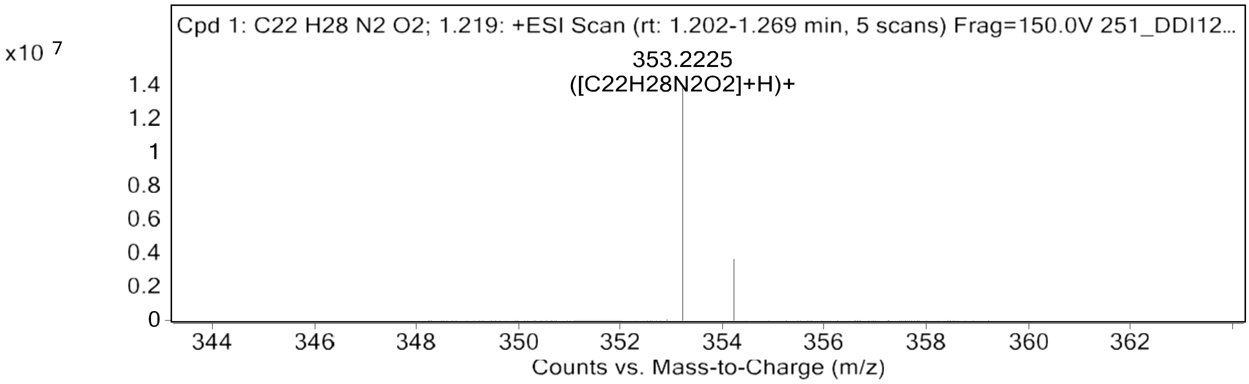

MS Spectrum Peak List

m/z	*Calc m/z*	Diff(ppm)	z	Abund	Formula	Ion
353.2225	353.2224	0.48	1	14037230	C22H28N2O2	(M+H)+
354.2258	354.2256	0.58	1	3672781.25	C22H28N2O2	(M+H)+

--- End Of Report ---

Compound 19

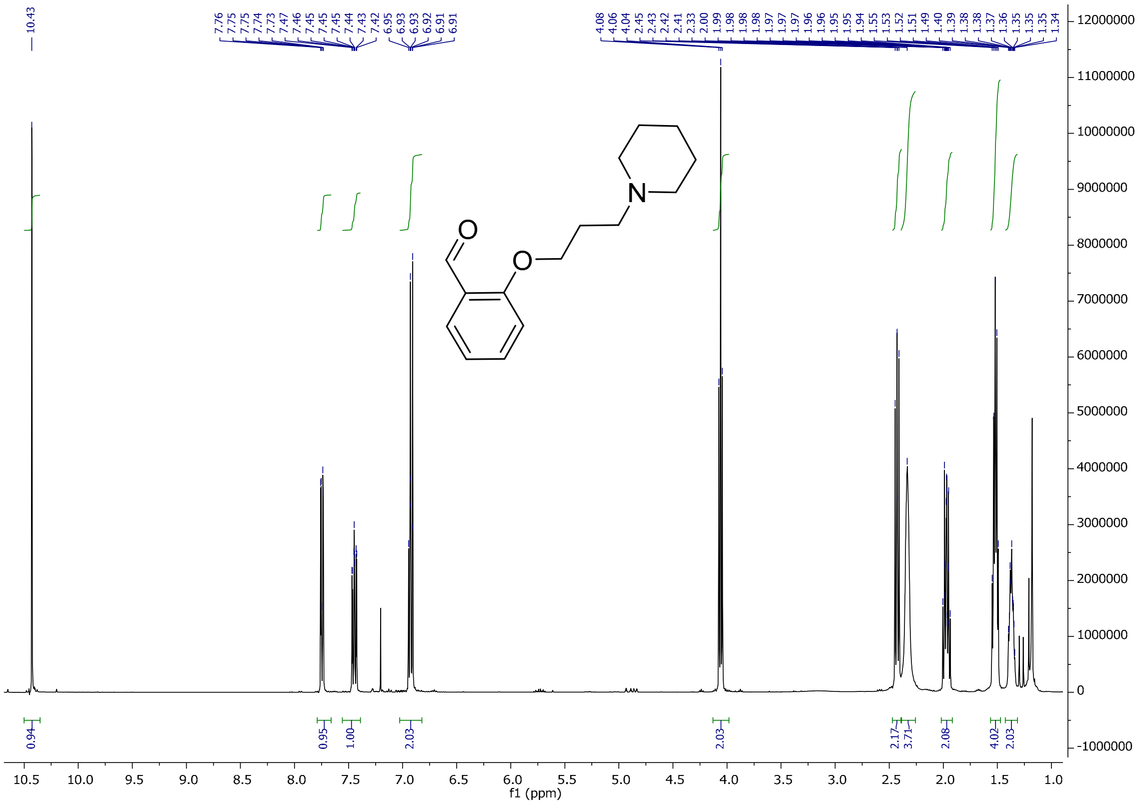
10.43
7.76 7.75 7.75 7.74 7.73 7.47 7.46 7.45 7.45 7.45 7.44 7.43 7.42 6.95 6.93 6.93 6.92 6.91 6.91
4.08 4.06 4.04 2.45 2.43 2.42 2.41 2.33 2.00 1.99 1.98 1.98 1.98 1.97 1.97 1.97 1.96 1.96 1.95 1.95 1.94 1.55 1.53 1.53 1.52 1.51 1.49 1.40 1.39 1.38 1.38 1.37 1.36 1.35 1.35 1.35 1.34
O
O
N
0.94
0.95
1.00
2.03
2.03
2.17
3.71
2.08
4.02
2.03
f1 (ppm)

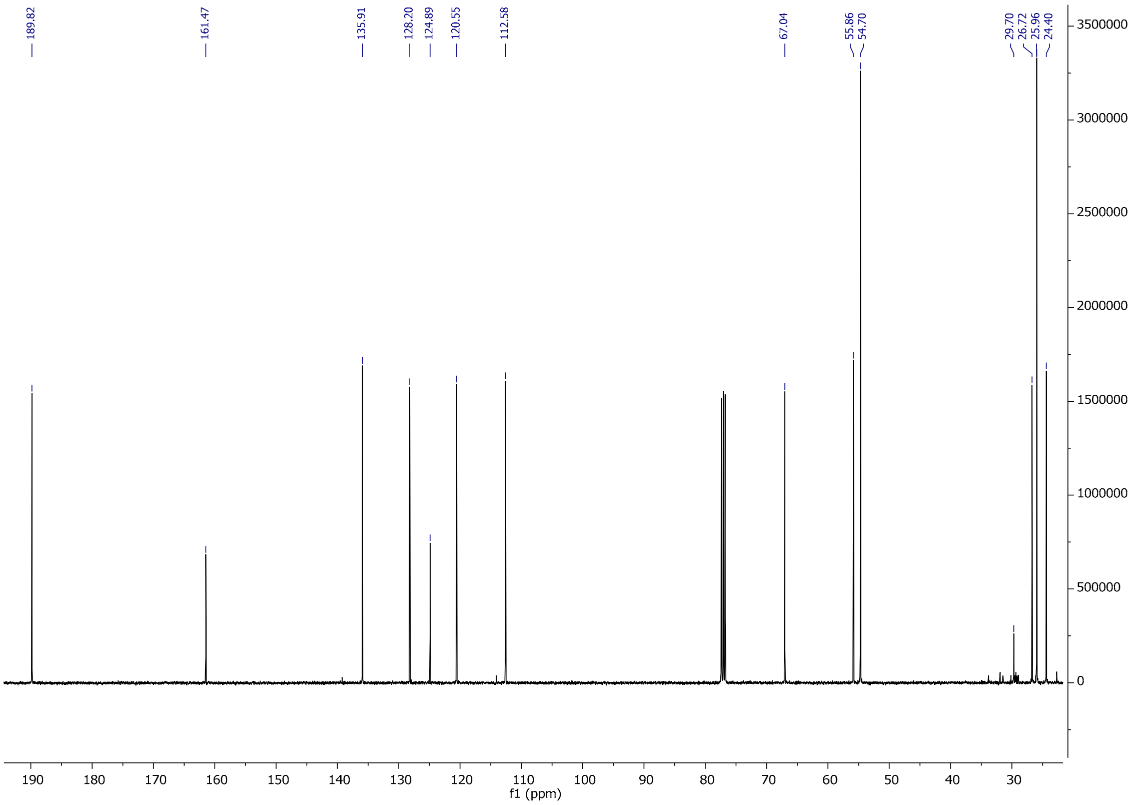
189.82
161.47
135.91
128.20
124.89
120.55
112.58
67.04
55.86
54.70
29.70
26.72
25.96
24.40
f1 (ppm)

Qualitative Compound Report

**Data File**	1529_DDI130_01.d	**Sample Name**	DDI130
**Sample Type**	Sample	**Position**	Vial 17
**Instrument Name**	Instrument 1	**User Name**	
**Acq Method**	ESI_ACN_75_pos_new.m	**Acquired Time**	6/9/2022 2:50:27 PM (UTC+02:00)
**IRM Calibration Status**	Success	**DA Method**	Defecto_modificado.m
**Comment**			

**Sample Group**		**Info.**	
**User**	MIREIA TOLEDANO	**Stream Name**	LC 1
**Acquisition Time (Local)**	6/9/2022 2:50:27 PM (UTC+02:00)	**Acquisition SW Version**	6200 series TOF/6500 series Q-TOF B.08.00 (B8058.3 SP1)
**QTOF Driver Version**	8.00.00	**QTOF Firmware Version**	2.712
**Tune Mass Range Max.**	3200		

Compound Table

Compound Label	RT	Mass	Abund	Formula	Tgt Mass	Diff (ppm)	Hits (DB)
Cpd 1: C15 H21 N O2; 0.106	0.106	247.1574	225721	C15 H21 N O2	247.1572	0.81	1

Compound Label	*m/z*	RT	Algorithm	Mass
Cpd 1: C15 H21 N O2; 0.106	248.1648	0.106	Find by Formula	247.1574

MS Zoomed Spectrum

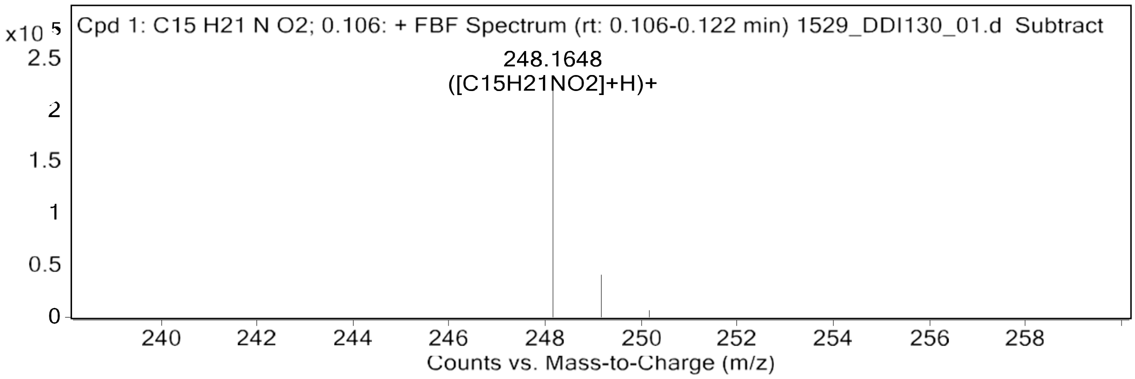

MS Spectrum Peak List

m/z	z	Abund	Formula	Ion
248.1648	1	225720.94	C15H21NO2	(M+H)+
249.1676	1	41122.21	C15H21NO2	(M+H)+
250.1699	1	6849.54	C15H21NO2	(M+H)+

MS Zoomed Spectrum

Qualitative Compound Report

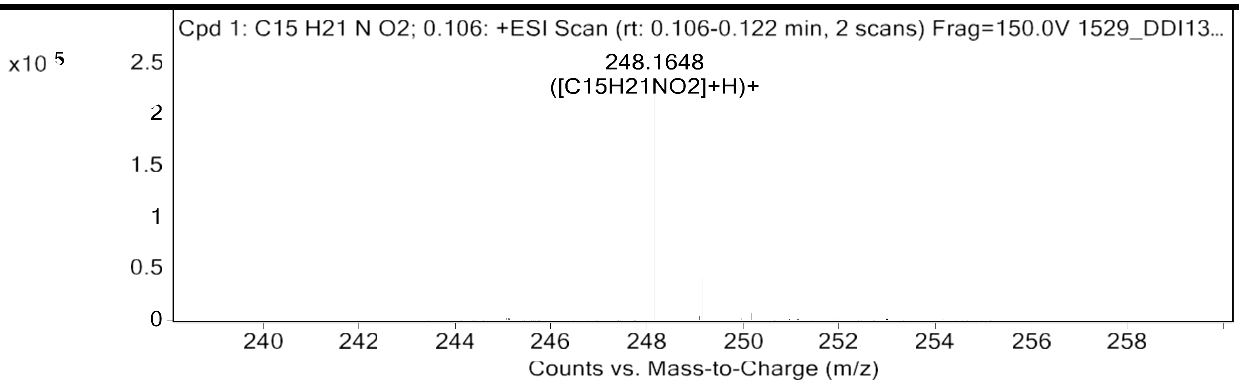

MS Spectrum Peak List

m/z	*Calc m/z*	Diff(ppm)	z	Abund	Formula	Ion
248.1648	248.1645	1.17	1	225720.94	C15H21NO2	(M+H)+
249.1676	249.1678	-0.67	1	41122.21	C15H21NO2	(M+H)+
250.1699	250.1705	-2.5	1	6849.54	C15H21NO2	(M+H)+

--- End Of Report ---

Nitrone 5

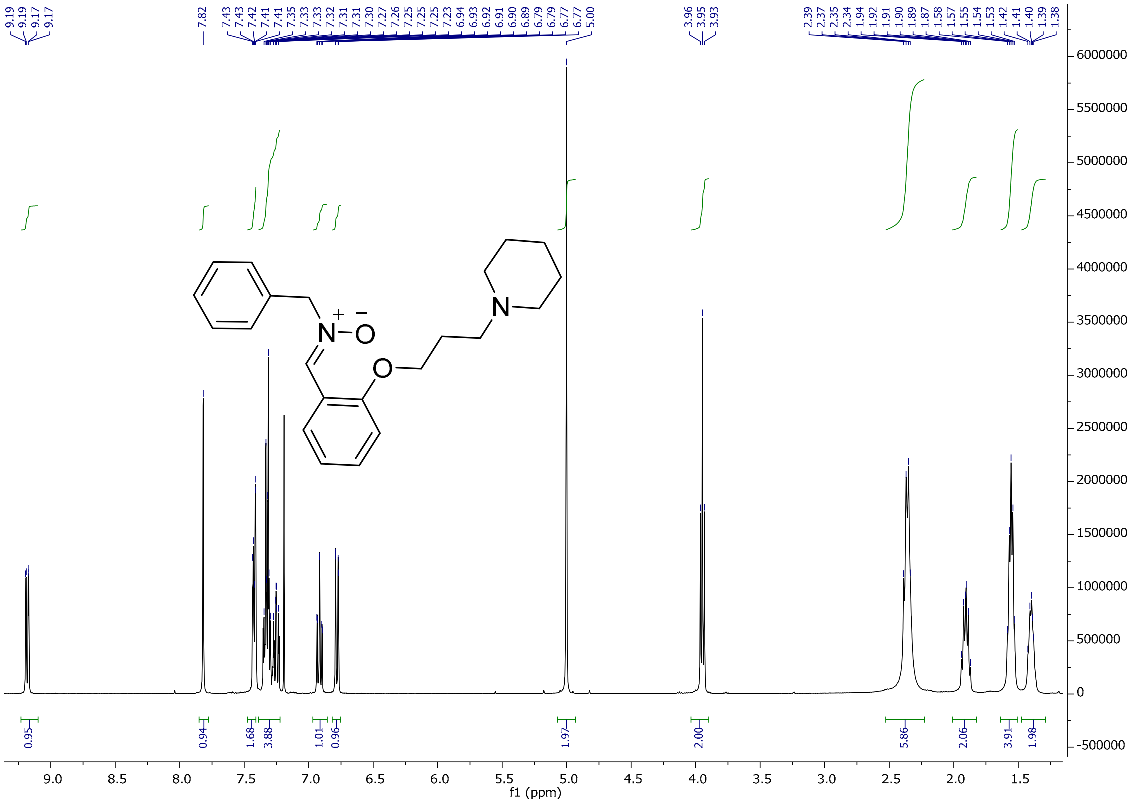
9.19 9.19 9.17 9.17
7.82
7.43 7.43 7.42 7.41 7.41 7.35 7.33 7.33 7.32 7.31 7.31 7.30 7.27 7.26 7.25 7.25 7.25 7.23 6.94 6.93 6.92 6.91 6.90 6.89 6.79 6.79 6.77 6.77 5.00
3.96 3.95 3.93
2.39 2.37 2.35 2.34 1.94 1.92 1.91 1.90 1.89 1.87 1.58 1.57 1.55 1.54 1.53 1.42 1.41 1.40 1.39 1.38
0.95 0.94 1.68 3.88 1.01 0.96 1.97 2.00 5.86 2.06 3.91 1.98
N
O
O
+
−
f1 (ppm)

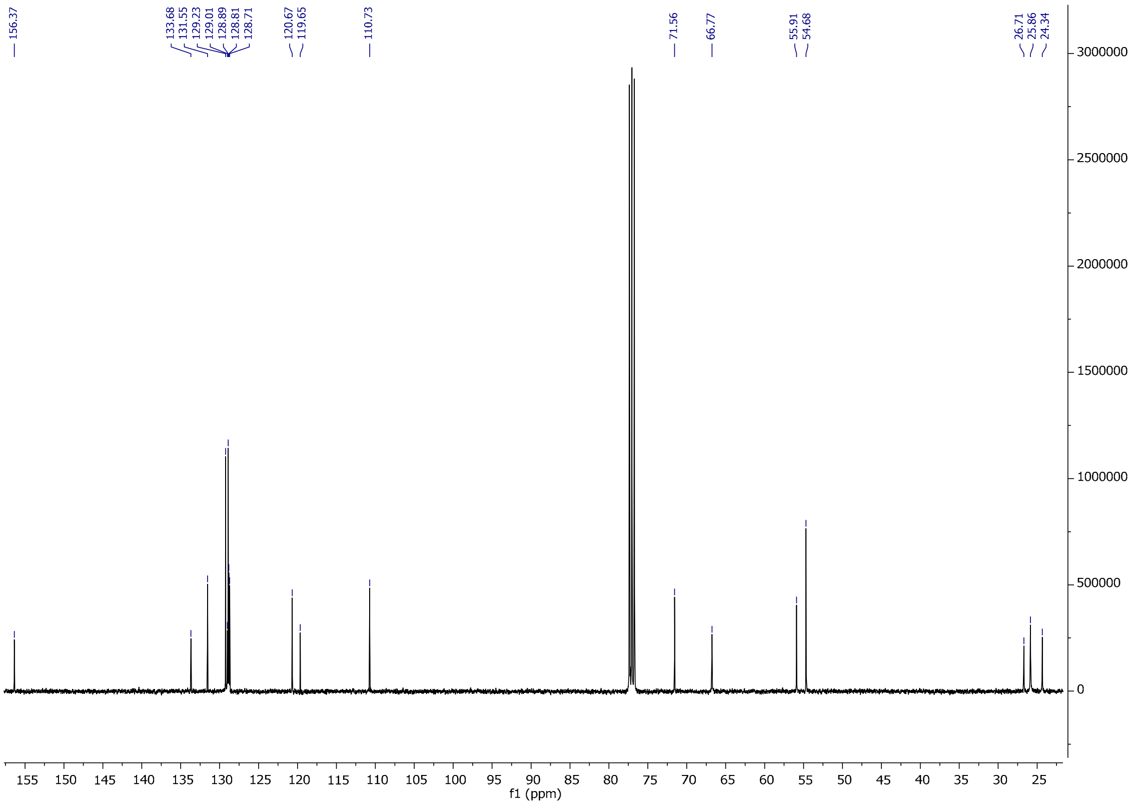
156.37
133.68 131.55 129.23 129.01 128.89 128.81 128.71
120.67 119.65
110.73
71.56
66.77
55.91 54.68
26.71 25.86 24.34
f1 (ppm)

Qualitative Compound Report

**Data File**	509_DDI131_02.d	**Sample Name**	DDI131
**Sample Type**	Sample	**Position**	Vial 2
**Instrument Name**	Instrument 1	**User Name**	
**Acq Method**	ESI_ACN_75_pos_new.m	**Acquired Time**	5/27/2021 11:43:19 AM (UTC+01:00)
**IRM Calibration Status**	Success	**DA Method**	Defecto_modificado.m
**Comment**			

**Sample Group**		**Info.**	
**User**	DANIEL DIEZ	**Stream Name**	LC 1
**Acquisition Time (Local)**	5/27/2021 11:43:19 AM (UTC+01:00)	**Acquisition SW Version**	6200 series TOF/6500 series Q-TOF B.08.00 (B8058.3 SP1)
**QTOF Driver Version**	8.00.00	**QTOF Firmware Version**	2.712
**Tune Mass Range Max.**	1700		

Compound Table

Compound Label	RT	Mass	Abund	Formula	Tgt Mass	Diff (ppm)	Hits (DB)
Cpd 1: C22 H28 N2 O2; 1.967	1.967	352.2149	1323828	C22 H28 N2 O2	352.2151	-0.54	1

Compound Label	*m/z*	RT	Algorithm	Mass
Cpd 1: C22 H28 N2 O2; 1.967	353.2221	1.967	Find by Formula	352.2149

MS Zoomed Spectrum

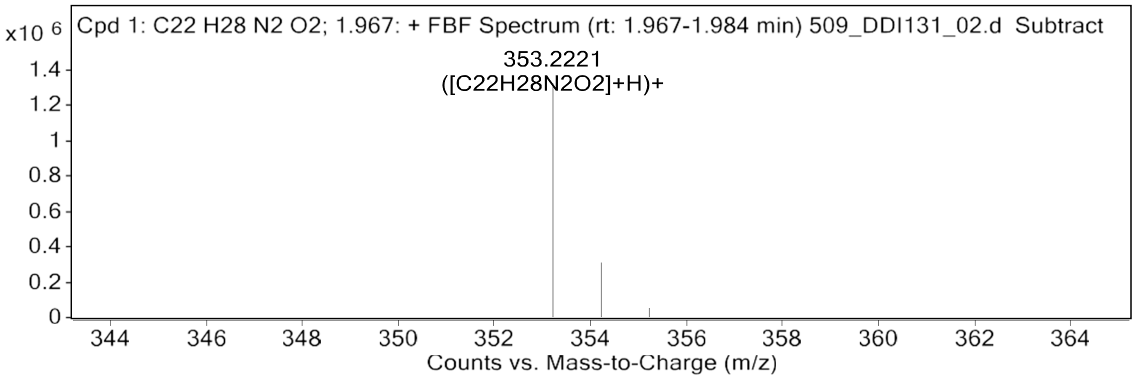

MS Spectrum Peak List

m/z	z	Abund	Formula	Ion
353.2221	1	1323828	C22H28N2O2	(M+H)+
354.2258	1	309089	C22H28N2O2	(M+H)+
355.2267	1	52167.43	C22H28N2O2	(M+H)+

MS Zoomed Spectrum

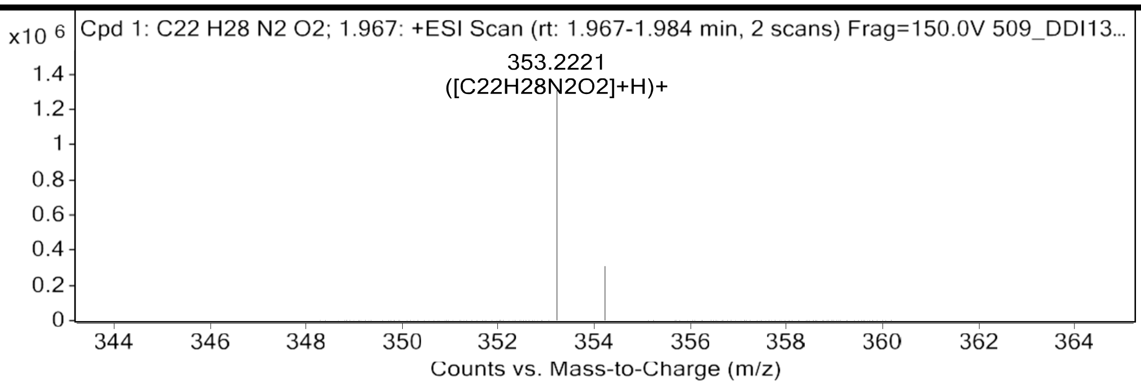

MS Spectrum Peak List

m/z	*Calc m/z*	Diff(ppm)	z	Abund	Formula	Ion
353.2221	353.2224	-0.65	1	1323828	C22H28N2O2	(M+H)+
354.2258	354.2256	0.66	1	309089	C22H28N2O2	(M+H)+
355.2267	355.2285	-4.98	1	52167.43	C22H28N2O2	(M+H)+

--- End Of Report ---

Compound 20

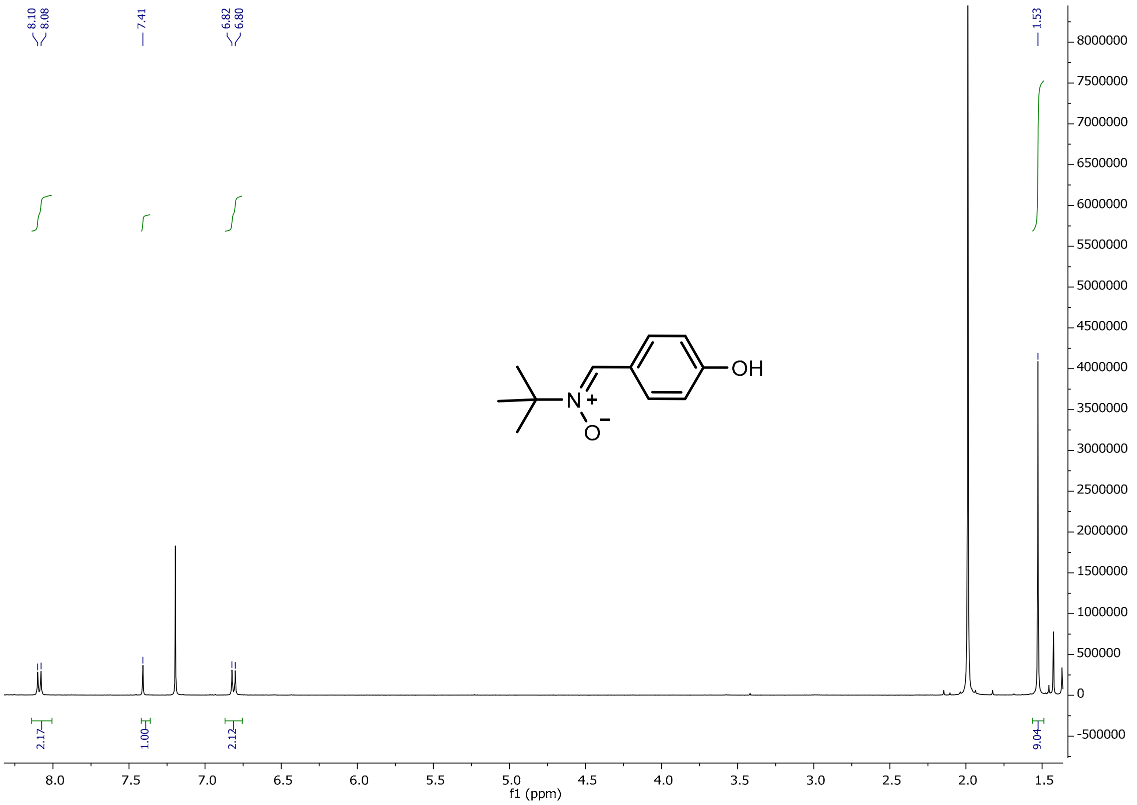

8.10
8.08
7.41
6.82
6.80
1.53
2.17
1.00
2.12
9.04
OH
N
O
f1 (ppm)

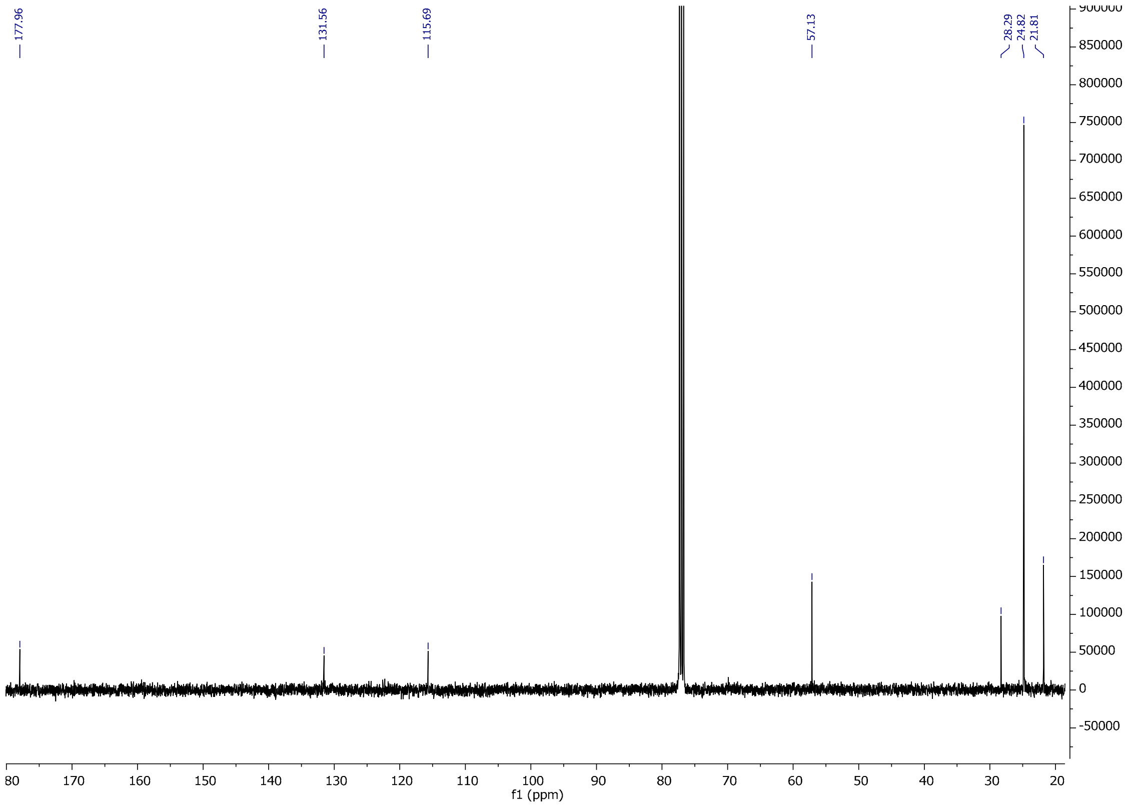

177.96
131.56
115.69
57.13
28.29
24.82
21.81
f1 (ppm)

Qualitative Compound Report

**Data File**	1530_DDI139_01.d	**Sample Name**	DDI139
**Sample Type**	Sample	**Position**	Vial 18
**Instrument Name**	Instrument 1	**User Name**	
**Acq Method**	ESI_ACN_75_pos_new.m	**Acquired Time**	6/9/2022 2:57:45 PM (UTC+02:00)
**IRM Calibration Status**	Success	**DA Method**	Defecto_modificado.m
**Comment**			

**Sample Group**		**Info.**	
**User**	MIREIA TOLEDANO	**Stream Name**	LC 1
**Acquisition Time (Local)**	6/9/2022 2:57:45 PM (UTC+02:00)	**Acquisition SW Version**	6200 series TOF/6500 series Q-TOF B.08.00 (B8058.3 SP1)
**QTOF Driver Version**	8.00.00	**QTOF Firmware Version**	2.712
**Tune Mass Range Max.**	3200		

Compound Table

Compound Label	RT	Mass	Abund	Formula	Tgt Mass	Diff (ppm)	Hits (DB)
Cpd 1: C11 H15 N O2; 1.335	1.335	193.1108	170228	C11 H15 N O2	193.1103	2.82	1

Compound Label	*m/z*	RT	Algorithm	Mass
Cpd 1: C11 H15 N O2; 1.335	194.1181	1.335	Find by Formula	193.1108

MS Zoomed Spectrum

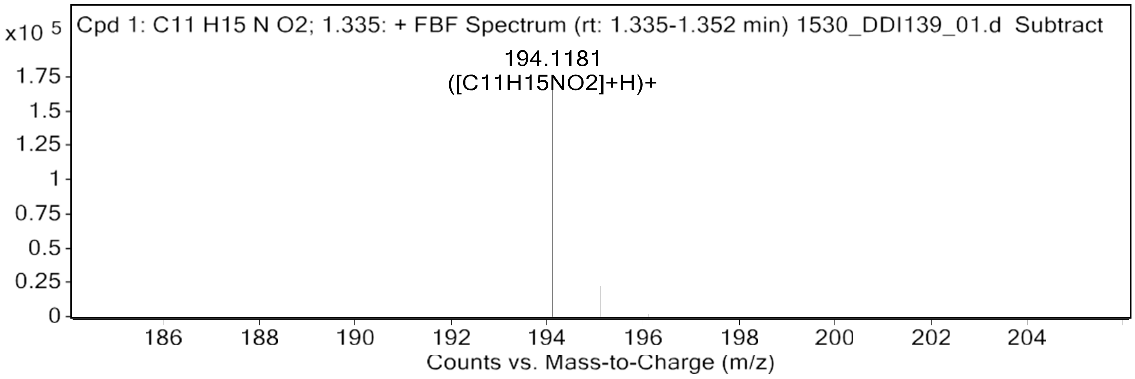

MS Spectrum Peak List

m/z	z	Abund	Formula	Ion
194.1181	1	170228.08	C11H15NO2	(M+H)+
195.1213	1	22448.3	C11H15NO2	(M+H)+
196.123	1	1998.05	C11H15NO2	(M+H)+

MS Zoomed Spectrum

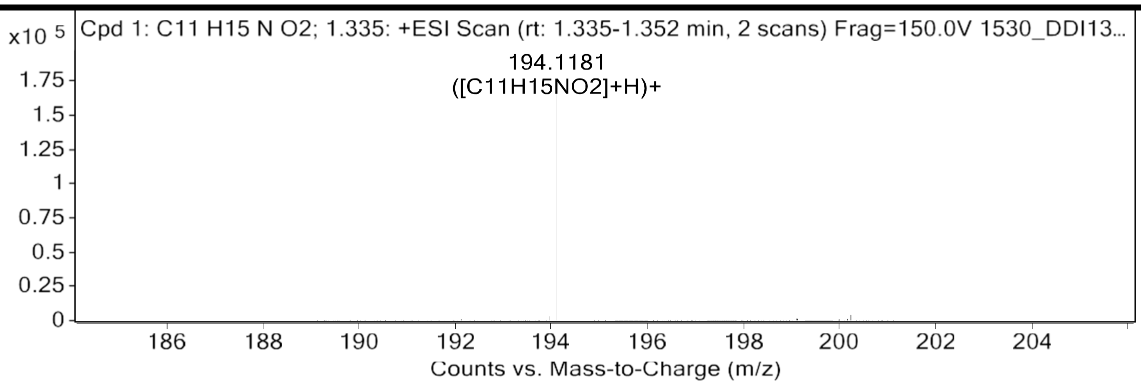

MS Spectrum Peak List

m/z	*Calc m/z*	Diff(ppm)	z	Abund	Formula	Ion
194.1181	194.1176	2.85	1	170228.08	C11H15NO2	(M+H)+
195.1213	195.1208	2.75	1	22448.3	C11H15NO2	(M+H)+
196.123	196.1232	-1.06	1	1998.05	C11H15NO2	(M+H)+

--- End Of Report ---

Nitrone 2

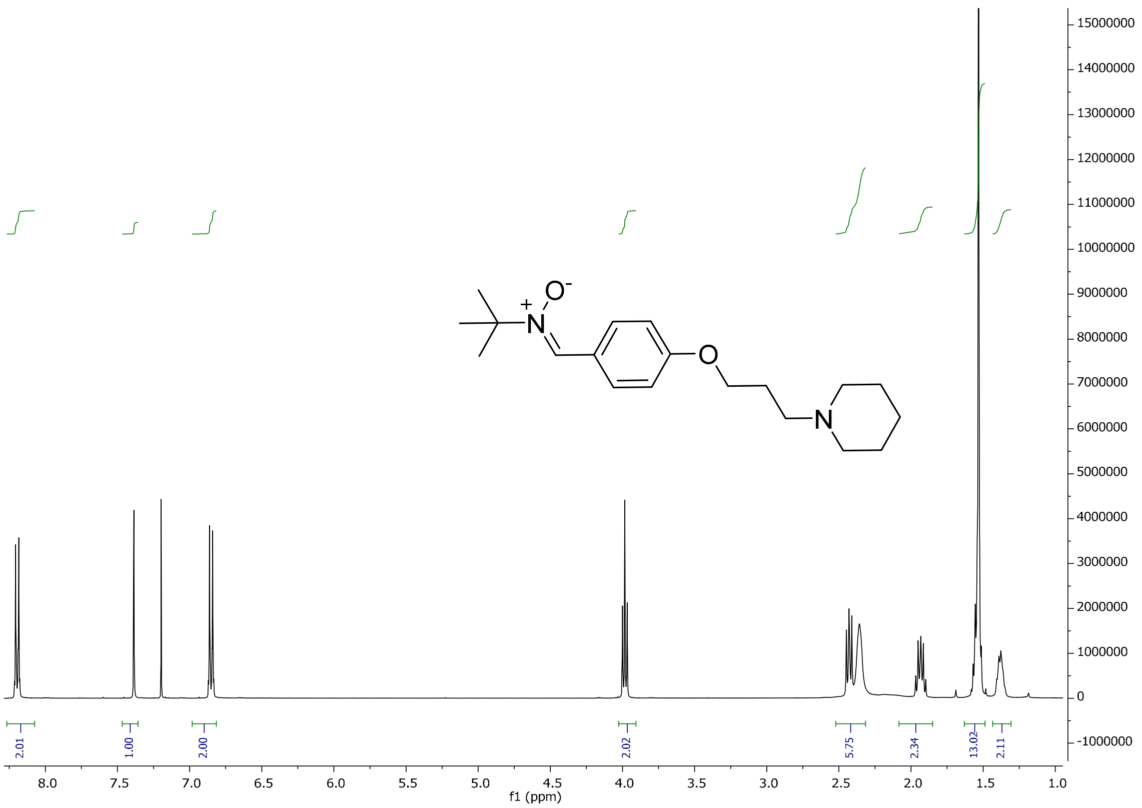

2.01
1.00
2.00
2.02
5.75
2.34
13.02
2.11
f1 (ppm)

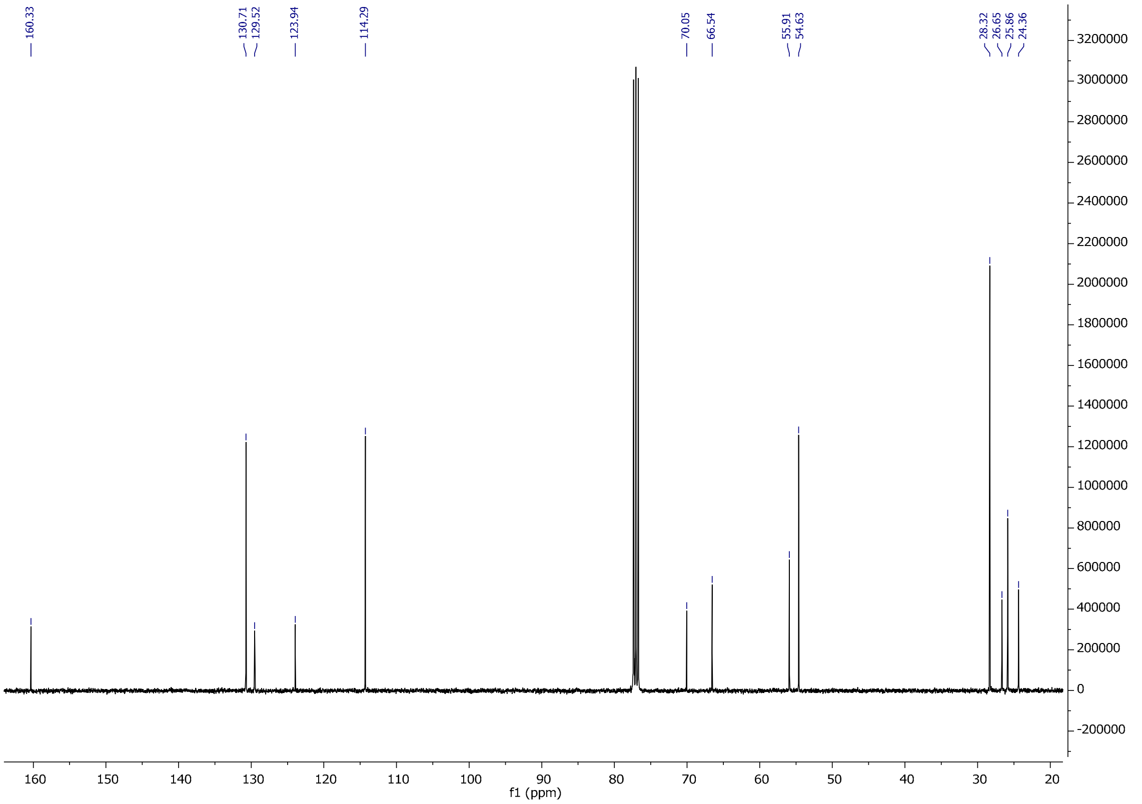

160.33
130.71
129.52
123.94
114.29
70.05
66.54
55.91
54.63
28.32
26.65
25.86
24.36
f1 (ppm)

Qualitative Compound Report

**Data File**	513_DDI140_01.d	**Sample Name**	DDI140
**Sample Type**	Sample	**Position**	Vial 6
**Instrument Name**	Instrument 1	**User Name**	
**Acq Method**	ESI_ACN_75_pos_new.m	**Acquired Time**	5/27/2021 12:22:23 PM (UTC+01:00)
**IRM Calibration Status**	Success	**DA Method**	Defecto_modificado.m
**Comment**			

**Sample Group**		**Info.**	
**User**	DANIEL DIEZ	**Stream Name**	LC 1
**Acquisition Time (Local)**	5/27/2021 12:22:23 PM (UTC+01:00)	**Acquisition SW Version**	6200 series TOF/6500 series Q-TOF B.08.00 (B8058.3 SP1)
**QTOF Driver Version**	8.00.00	**QTOF Firmware Version**	2.712
**Tune Mass Range Max.**	1700		

Compound Table

Compound Label	RT	Mass	Abund	Formula	Tgt Mass	Diff (ppm)	Hits (DB)
Cpd 1: C19 H30 N2 O2; 2.240	2.24	318.2306	1445475	C19 H30 N2 O2	318.2307	-0.35	1

Compound Label	*m/z*	RT	Algorithm	Mass
Cpd 1: C19 H30 N2 O2; 2.240	319.2379	2.24	Find by Formula	318.2306

MS Zoomed Spectrum

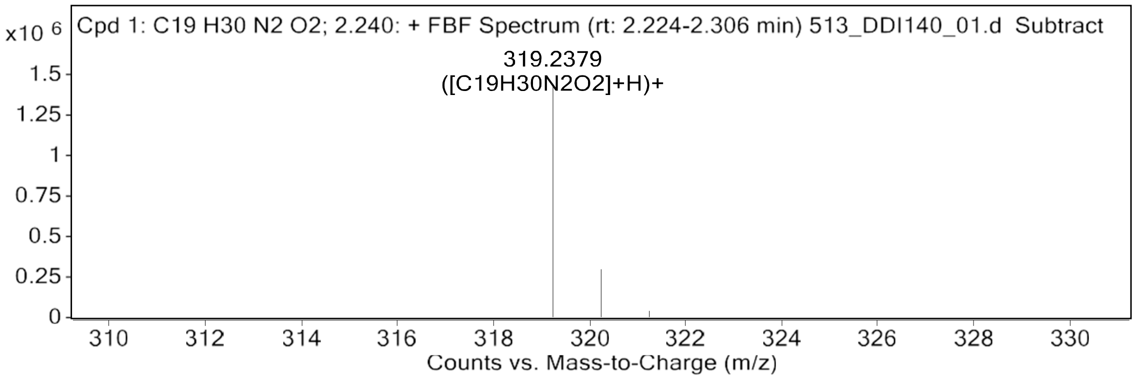

MS Spectrum Peak List

m/z	z	Abund	Formula	Ion
319.2379	1	1445475.13	C19H30N2O2	(M+H)+
320.2409	1	296249.41	C19H30N2O2	(M+H)+
321.2437	1	38924.45	C19H30N2O2	(M+H)+

MS Zoomed Spectrum

Qualitative Compound Report

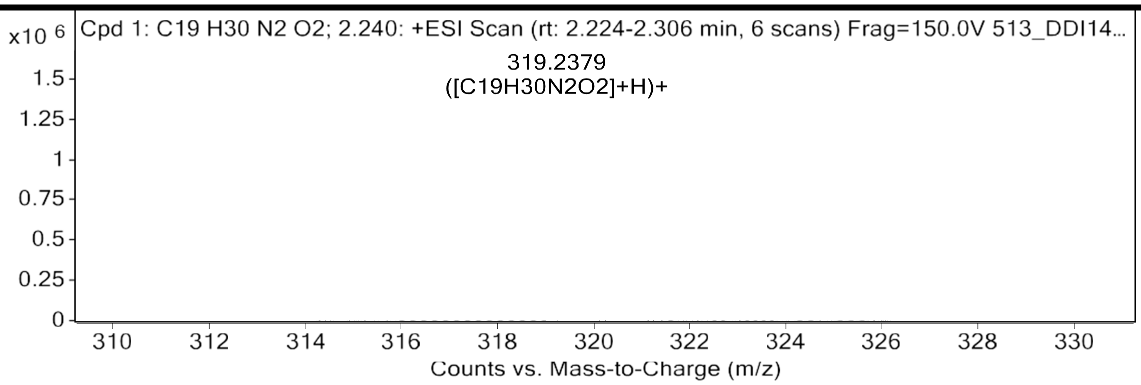

MS Spectrum Peak List

m/z	*Calc m/z*	Diff(ppm)	z	Abund	Formula	Ion
319.2379	319.238	-0.21	1	1445475.13	C19H30N2O2	(M+H)+
320.2409	320.2412	-0.89	1	296249.41	C19H30N2O2	(M+H)+
321.2437	321.2441	-1.08	1	38924.45	C19H30N2O2	(M+H)+

--- End Of Report ---

Compoiund 20

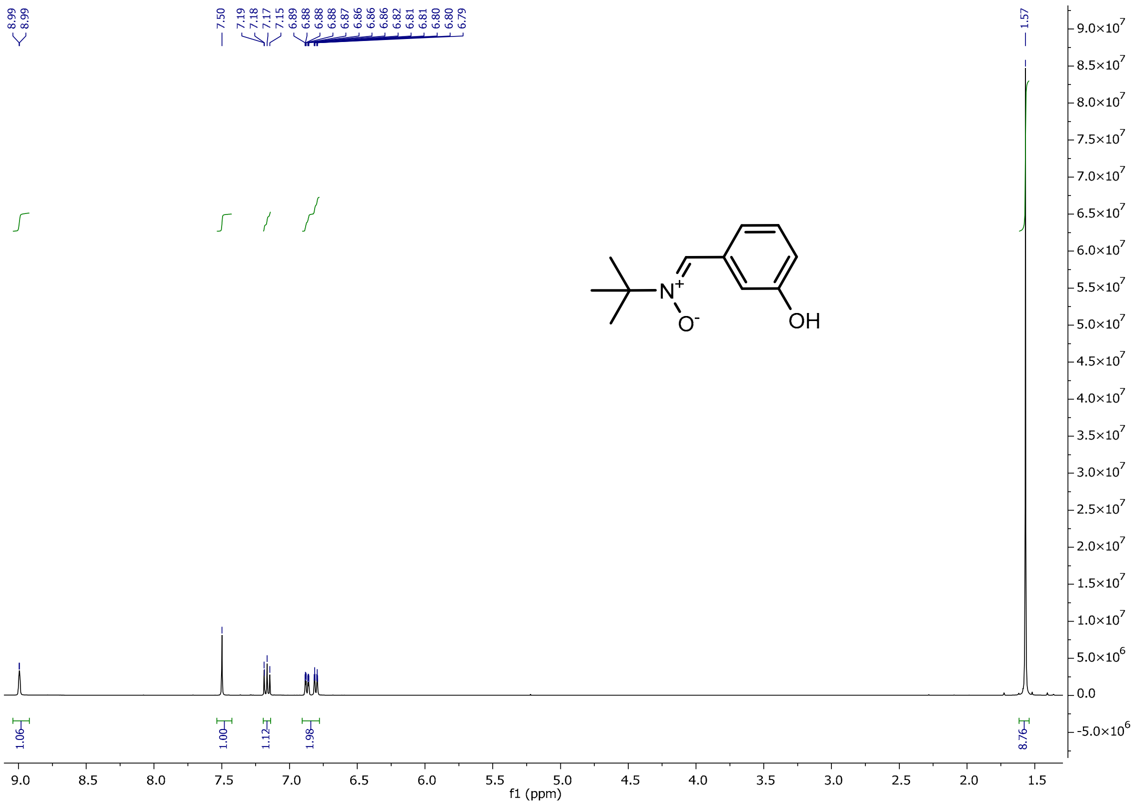

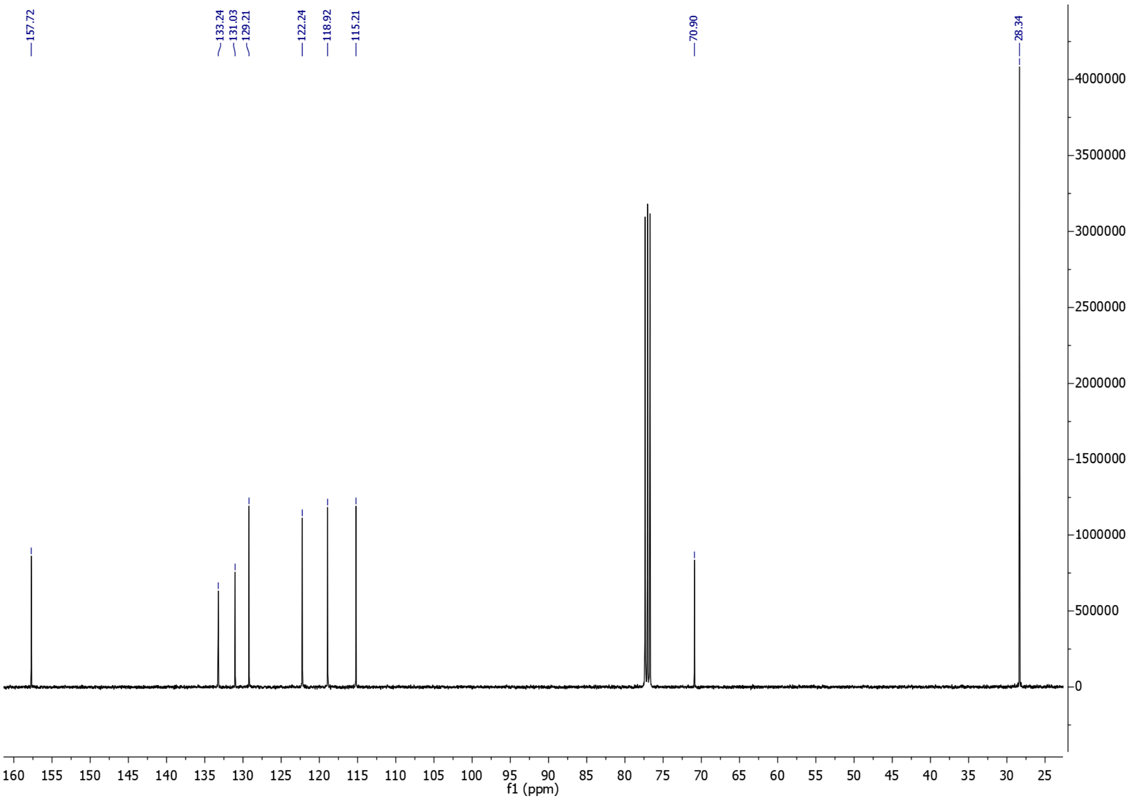

Qualitative Compound Report

**Data File**	1531_DDI141_01.d	**Sample Name**	DDI141
**Sample Type**	Sample	**Position**	Vial 19
**Instrument Name**	Instrument 1	**User Name**	
**Acq Method**	ESI_ACN_75_pos_new.m	**Acquired Time**	6/9/2022 3:04:59 PM (UTC+02:00)
**IRM Calibration Status**	Success	**DA Method**	Defecto_modificado.m
**Comment**			

**Sample Group**		**Info.**	
**User**	MIREIA TOLEDANO	**Stream Name**	LC 1
**Acquisition Time (Local)**	6/9/2022 3:04:59 PM (UTC+02:00)	**Acquisition SW Version**	6200 series TOF/6500 series Q-TOF B.08.00 (B8058.3 SP1)
**QTOF Driver Version**	8.00.00	**QTOF Firmware Version**	2.712
**Tune Mass Range Max.**	3200		

Compound Table

Compound Label	RT	Mass	Abund	Formula	Tgt Mass	Diff (ppm)	Hits (DB)
Cpd 1: C11 H15 N O2; 0.228	0.228	193.1101	6602274	C11 H15 N O2	193.1103	-1.18	1

Compound Label	*m/z*	RT	Algorithm	Mass
Cpd 1: C11 H15 N O2; 0.228	194.1172	0.228	Find by Formula	193.1101

MS Zoomed Spectrum

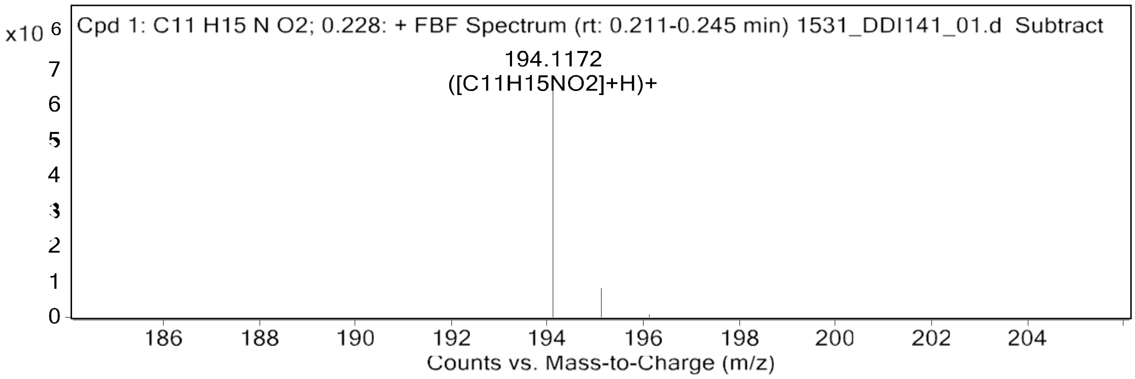

MS Spectrum Peak List

m/z	z	Abund	Formula	Ion
194.1172	1	6602273.5	C11H15NO2	(M+H)+
195.1213	1	825399.44	C11H15NO2	(M+H)+
196.1238	1	79819.62	C11H15NO2	(M+H)+

MS Zoomed Spectrum

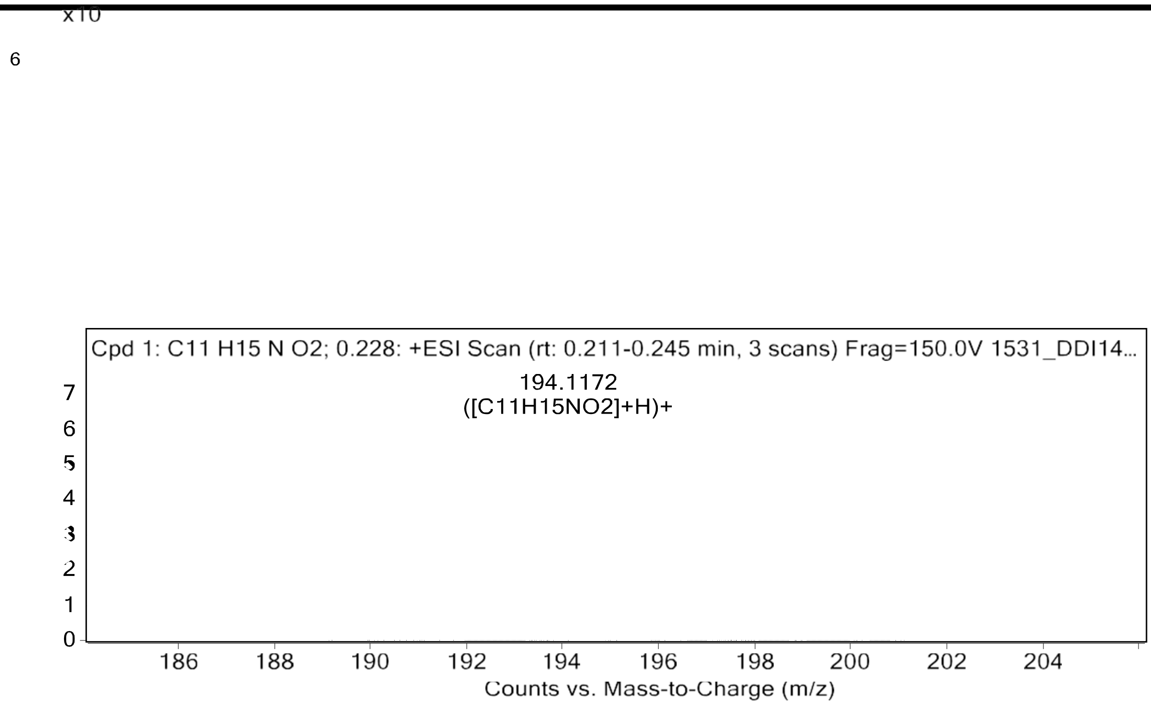

MS Spectrum Peak List

m/z	*Calc m/z*	Diff(ppm)	z	Abund	Formula	Ion
194.1172	194.1176	-1.71	1	6602273.5	C11H15NO2	(M+H)+
195.1213	195.1208	2.71	1	825399.44	C11H15NO2	(M+H)+
196.1238	196.1232	3.34	1	79819.62	C11H15NO2	(M+H)+

--- End Of Report ---

Nitrone 4

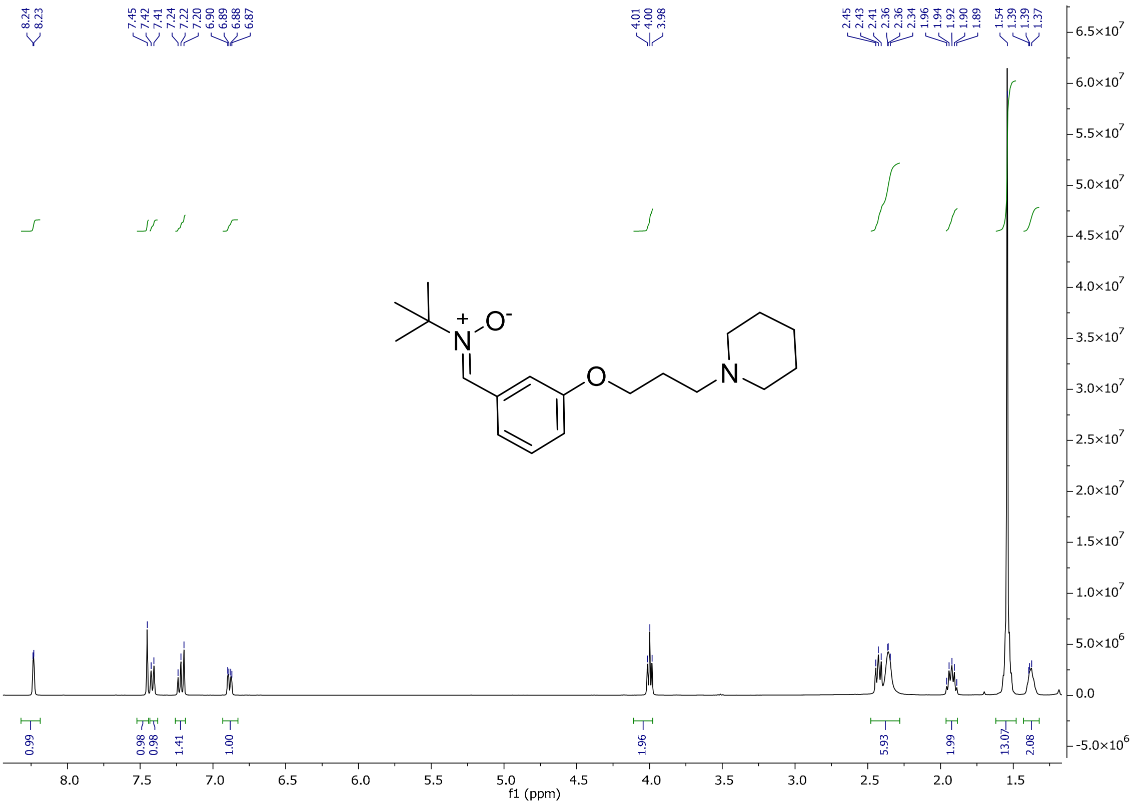
8.24
8.23
7.45
7.42
7.41
7.24
7.22
7.20
6.90
6.89
6.88
6.87
4.01
4.00
3.98
2.45
2.43
2.41
2.36
2.36
2.34
1.96
1.94
1.92
1.90
1.89
1.54
1.39
1.39
1.37
0.99
0.98
0.98
1.41
1.00
1.96
5.93
1.99
13.07
2.08
f1 (ppm)

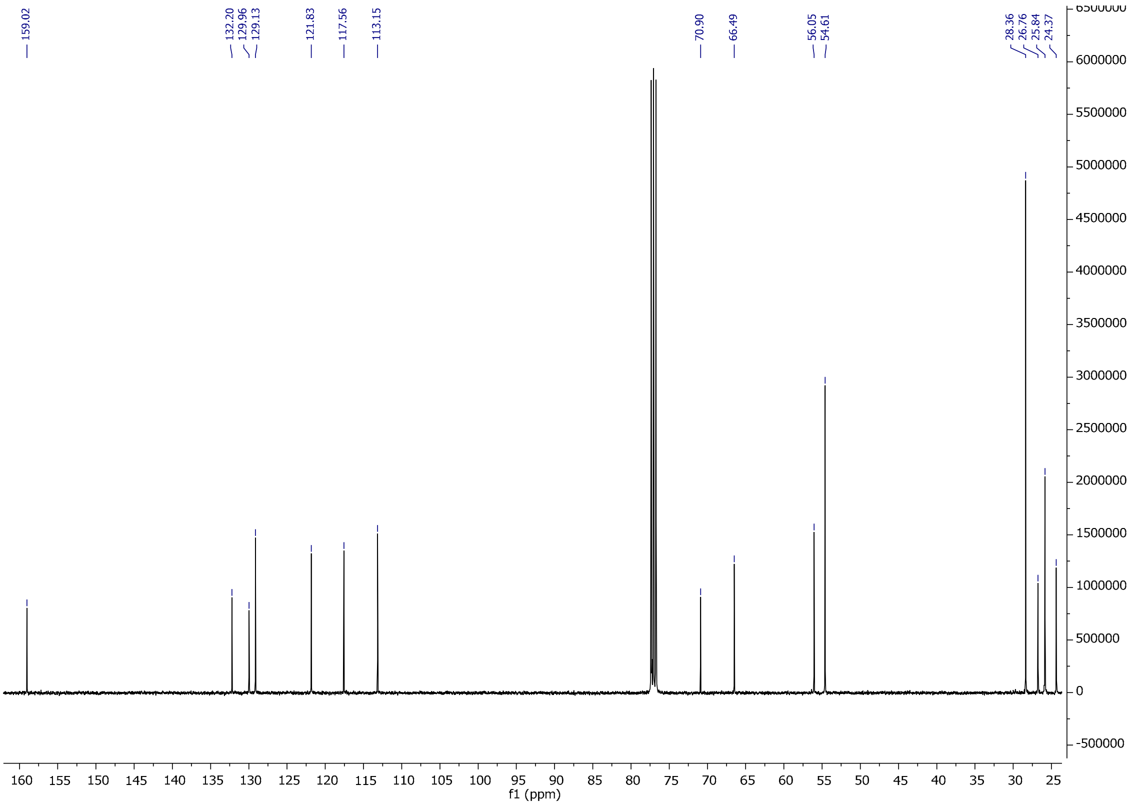
159.02
132.20
129.96
129.13
121.83
117.56
113.15
70.90
66.49
56.05
54.61
28.36
26.76
25.84
24.37
f1 (ppm)

Qualitative Compound Report

**Data File**	514_DDI143_01.d	**Sample Name**	DDI143
**Sample Type**	Sample	**Position**	Vial 7
**Instrument Name**	Instrument 1	**User Name**	
**Acq Method**	ESI_ACN_75_pos_new.m	**Acquired Time**	5/27/2021 12:29:38 PM (UTC+01:00)
**IRM Calibration Status**	Success	**DA Method**	Defecto_modificado.m
**Comment**			

**Sample Group**		**Info.**	
**User**	DANIEL DIEZ	**Stream Name**	LC 1
**Acquisition Time (Local)**	5/27/2021 12:29:38 PM (UTC+01:00)	**Acquisition SW Version**	6200 series TOF/6500 series Q-TOF B.08.00 (B8058.3 SP1)
**QTOF Driver Version**	8.00.00	**QTOF Firmware Version**	2.712
**Tune Mass Range Max.**	1700		

Compound Table

Compound Label	RT	Mass	Abund	Formula	Tgt Mass	Diff (ppm)	Hits (DB)
Cpd 1: C19 H30 N2 O2; 2.761	2.761	318.231	1310468	C19 H30 N2 O2	318.2307	0.72	1

Compound Label	*m/z*	RT	Algorithm	Mass
Cpd 1: C19 H30 N2 O2; 2.761	319.2383	2.761	Find by Formula	318.231

MS Zoomed Spectrum

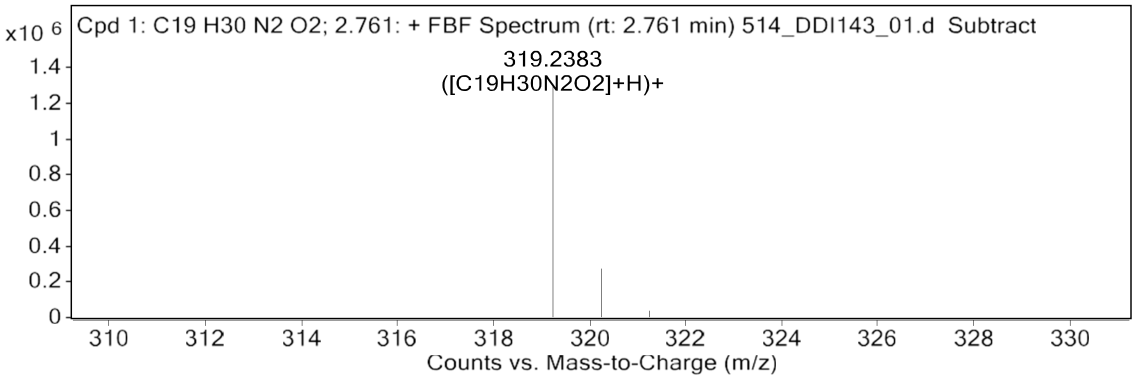

MS Spectrum Peak List

m/z	z	Abund	Formula	Ion
319.2383	1	1310468.38	C19H30N2O2	(M+H)+
320.2414	1	272774.84	C19H30N2O2	(M+H)+
321.2436	1	35761.39	C19H30N2O2	(M+H)+

MS Zoomed Spectrum

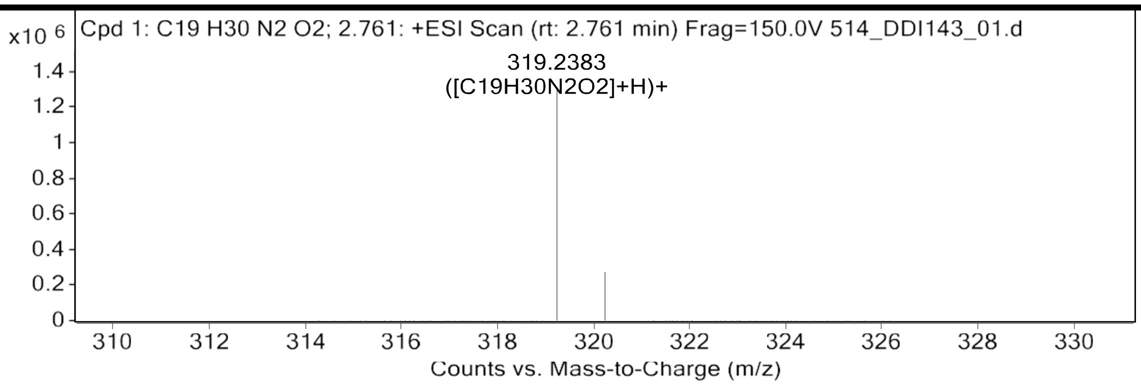

MS Spectrum Peak List

m/z	*Calc m/z*	Diff(ppm)	z	Abund	Formula	Ion
319.2383	319.238	0.79	1	1310468.38	C19H30N2O2	(M+H)+
320.2414	320.2412	0.7	1	272774.84	C19H30N2O2	(M+H)+
321.2436	321.2441	-1.55	1	35761.39	C19H30N2O2	(M+H)+

--- End Of Report ---

Compound 22

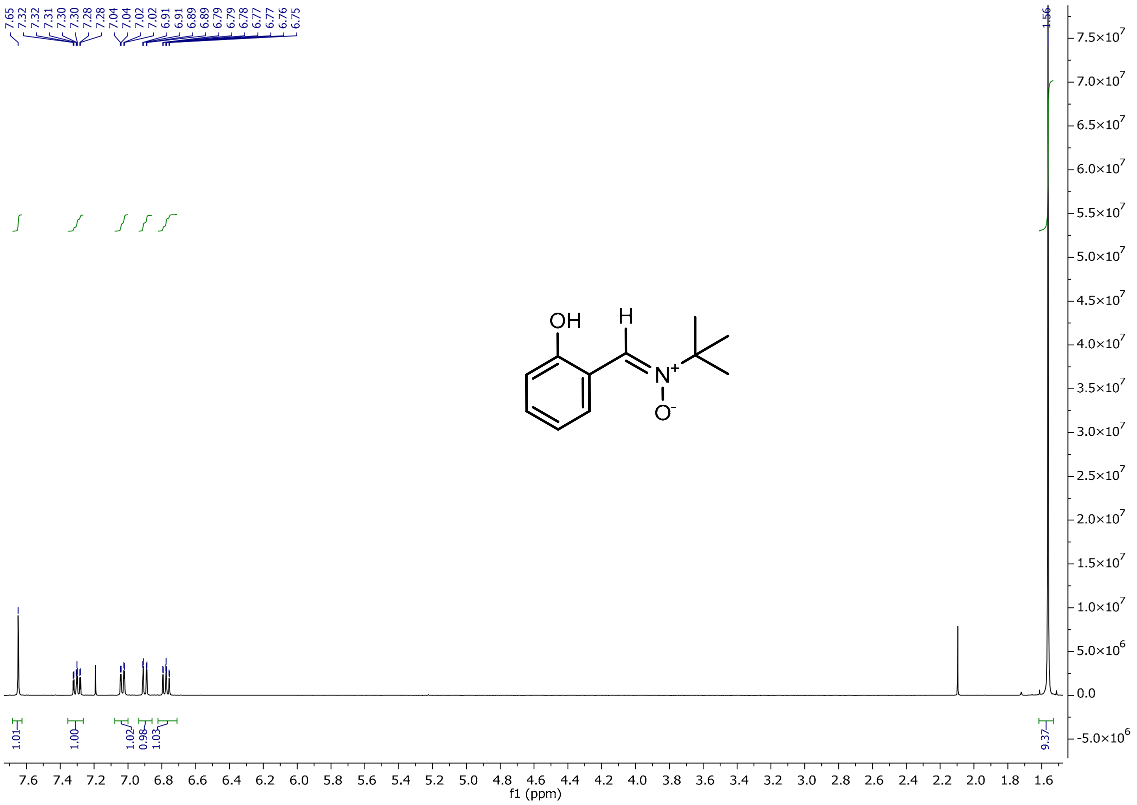
7.65
7.32
7.32
7.31
7.30
7.30
7.28
7.28
7.04
7.04
7.02
7.02
6.91
6.91
6.89
6.89
6.79
6.79
6.78
6.77
6.77
6.76
6.75
1.56
OH
H
N+
O-
1.01
1.00
1.02
0.98
1.03
9.37
f1 (ppm)

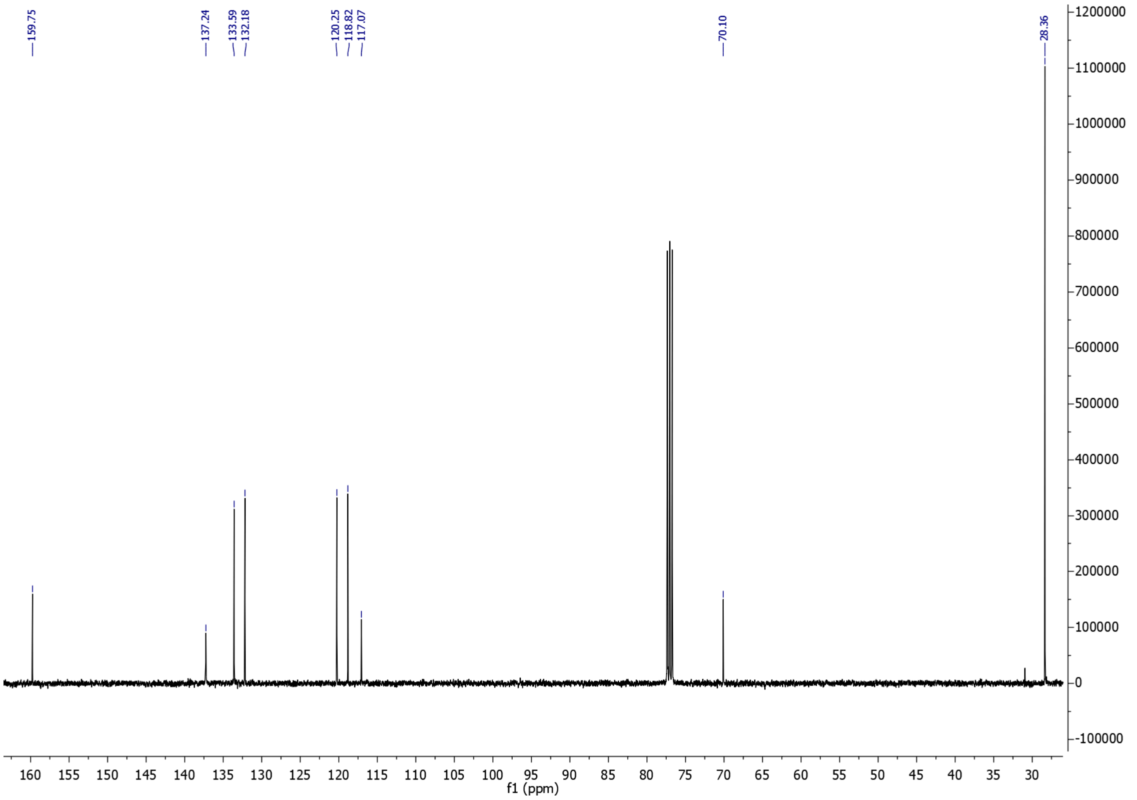
159.75
137.24
133.59
132.18
120.25
118.82
117.07
70.10
28.36
f1 (ppm)

Qualitative Compound Report

**Data File**	1532_DDI142_01.d	**Sample Name**	DDI142
**Sample Type**	Sample	**Position**	Vial 20
**Instrument Name**	Instrument 1	**User Name**	
**Acq Method**	ESI_ACN_75_pos_new.m	**Acquired Time**	6/9/2022 3:12:19 PM (UTC+02:00)
**IRM Calibration Status**	Success	**DA Method**	Defecto_modificado.m
**Comment**			

**Sample Group**		**Info.**	
**User**	MIREIA TOLEDANO	**Stream Name**	LC 1
**Acquisition Time (Local)**	6/9/2022 3:12:19 PM (UTC+02:00)	**Acquisition SW Version**	6200 series TOF/6500 series Q-TOF B.08.00 (B8058.3 SP1)
**QTOF Driver Version**	8.00.00	**QTOF Firmware Version**	2.712
**Tune Mass Range Max.**	3200		

Compound Table

Compound Label	RT	Mass	Abund	Formula	Tgt Mass	Diff (ppm)	Hits (DB)
Cpd 1: C11 H15 N O2; 1.751	1.751	193.1107	283003	C11 H15 N O2	193.1103	2.06	1

Compound Label	*m/z*	RT	Algorithm	Mass
Cpd 1: C11 H15 N O2; 1.751	194.118	1.751	Find by Formula	193.1107

MS Zoomed Spectrum

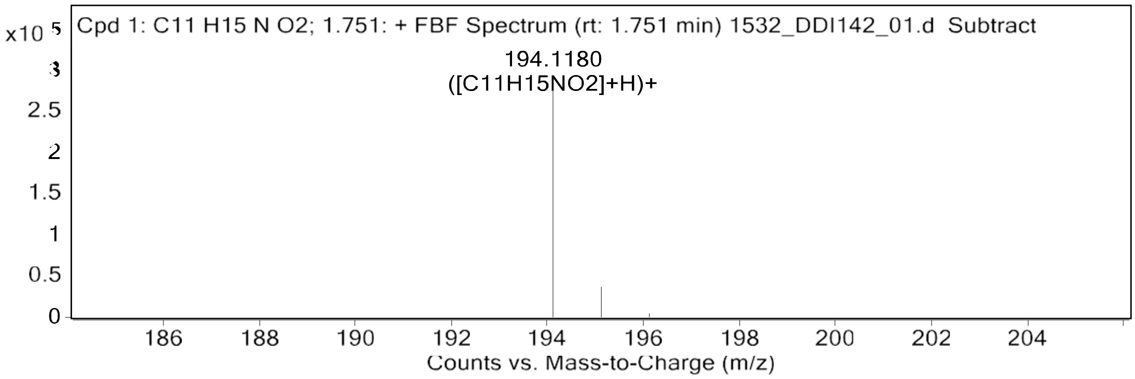

MS Spectrum Peak List

m/z	z	Abund	Formula	Ion
194.118	1	283002.78	C11H15NO2	(M+H)+
195.121	1	36890.37	C11H15NO2	(M+H)+
196.1234	1	4540.47	C11H15NO2	(M+H)+

MS Zoomed Spectrum

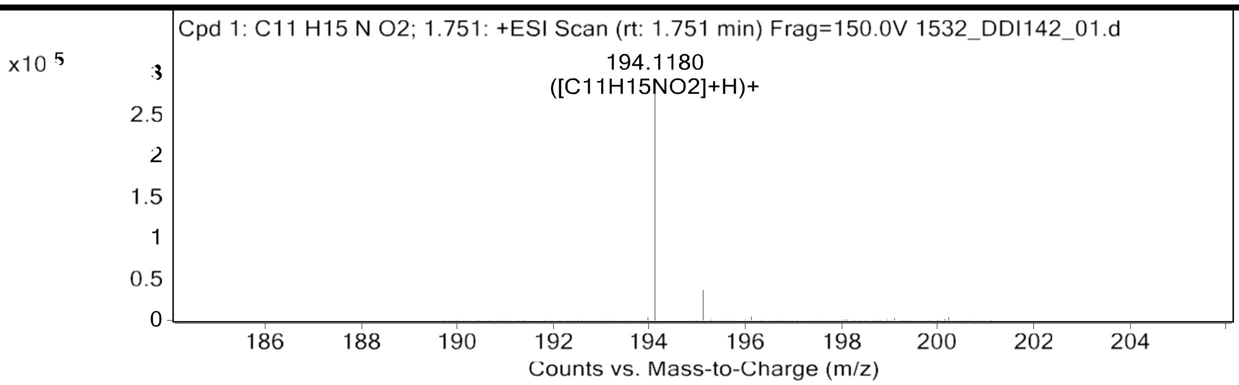

MS Spectrum Peak List

m/z	*Calc m/z*	Diff(ppm)	z	Abund	Formula	Ion
194.118	194.1176	2.18	1	283002.78	C11H15NO2	(M+H)+
195.121	195.1208	1.2	1	36890.37	C11H15NO2	(M+H)+
196.1234	196.1232	1.24	1	4540.47	C11H15NO2	(M+H)+

--- End Of Report ---

Nitrone 6

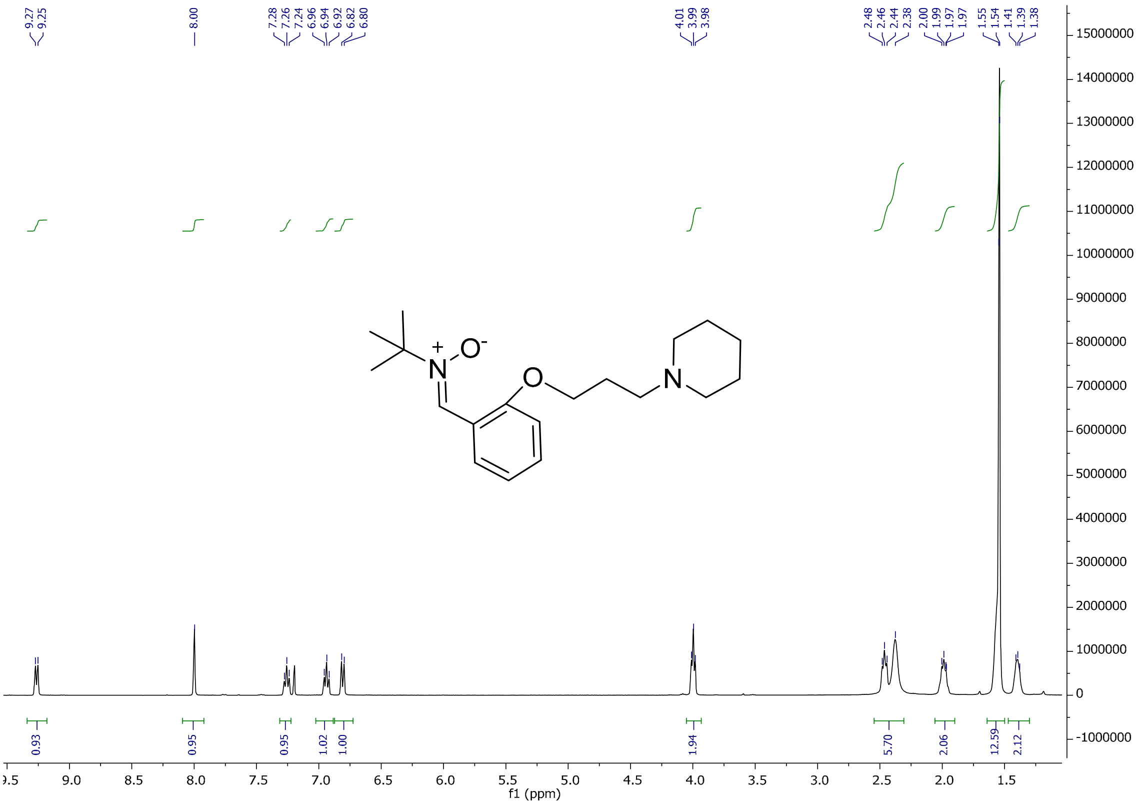

9.27
9.25
8.00
7.28
7.26
7.24
6.96
6.94
6.92
6.82
6.80
4.01
3.99
3.98
2.48
2.46
2.44
2.38
2.00
1.99
1.97
1.97
1.55
1.54
1.41
1.39
1.38
0.93
0.95
0.95
1.02
1.00
1.94
5.70
2.06
12.59
2.12
f1 (ppm)

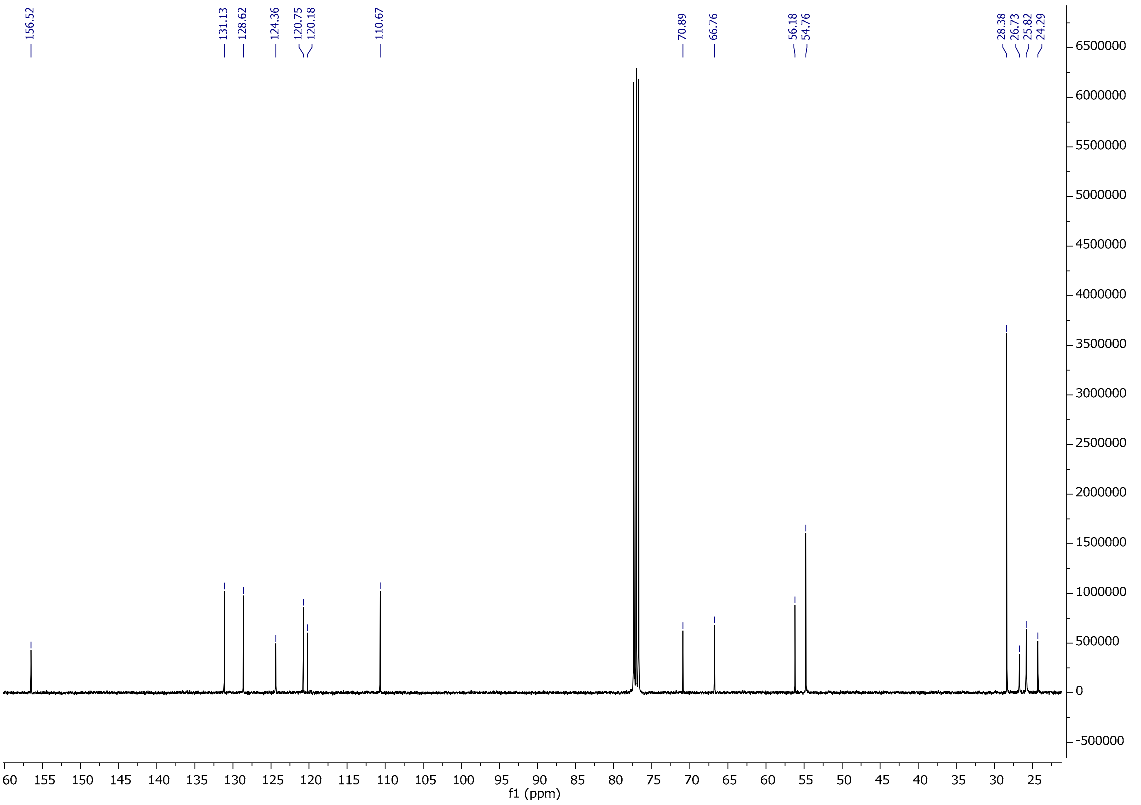

156.52
131.13
128.62
124.36
120.75
120.18
110.67
70.89
66.76
56.18
54.76
28.38
26.73
25.82
24.29
f1 (ppm)

Qualitative Compound Report

**Data File**	515_DDI144_01.d	**Sample Name**	DDI144
**Sample Type**	Sample	**Position**	Vial 8
**Instrument Name**	Instrument 1	**User Name**	
**Acq Method**	ESI_ACN_75_pos_new.m	**Acquired Time**	5/27/2021 12:36:53 PM (UTC+01:00)
**IRM Calibration Status**	Success	**DA Method**	Defecto_modificado.m
**Comment**			

**Sample Group**		**Info.**	
**User**	DANIEL DIEZ	**Stream Name**	LC 1
**Acquisition Time (Local)**	5/27/2021 12:36:53 PM (UTC+01:00)	**Acquisition SW Version**	6200 series TOF/6500 series Q-TOF B.08.00 (B8058.3 SP1)
**QTOF Driver Version**	8.00.00	**QTOF Firmware Version**	2.712
**Tune Mass Range Max.**	1700		

Compound Table

Compound Label	RT	Mass	Abund	Formula	Tgt Mass	Diff (ppm)	Hits (DB)
Cpd 1: C19 H30 N2 O2; 0.130	0.13	318.2305	249142	C19 H30 N2 O2	318.2307	-0.76	1

Compound Label	*m/z*	RT	Algorithm	Mass
Cpd 1: C19 H30 N2 O2; 0.130	319.2378	0.13	Find by Formula	318.2305

MS Zoomed Spectrum

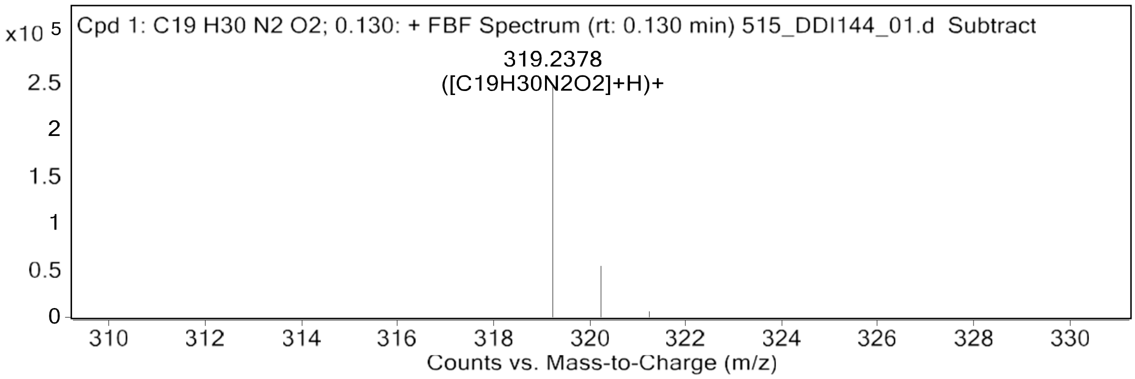

MS Spectrum Peak List

m/z	z	Abund	Formula	Ion
319.2378	1	249142.31	C19H30N2O2	(M+H)+
320.2405	1	54779.67	C19H30N2O2	(M+H)+
321.2457	1	6451.66	C19H30N2O2	(M+H)+

MS Zoomed Spectrum

Qualitative Compound Report

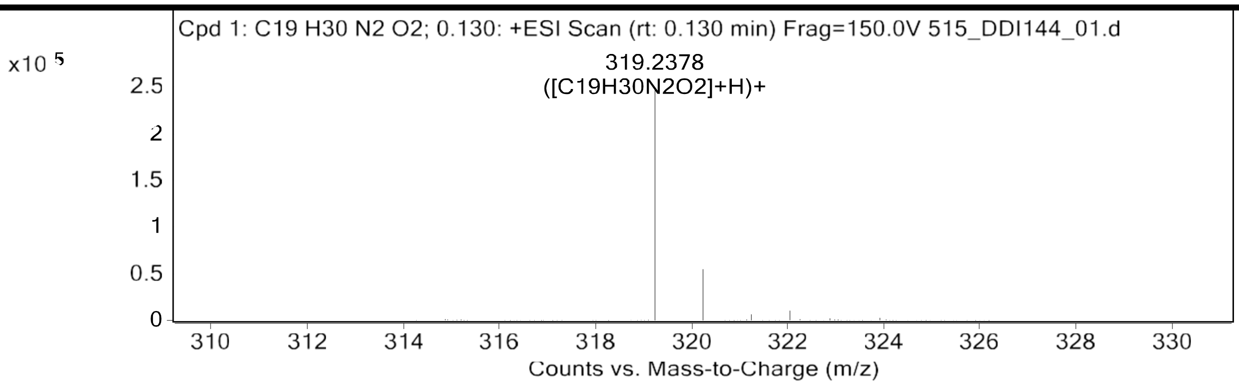

MS Spectrum Peak List

m/z	*Calc m/z*	Diff(ppm)	z	Abund	Formula	Ion
319.2378	319.238	-0.58	1	249142.31	C19H30N2O2	(M+H)+
320.2405	320.2412	-2.23	1	54779.67	C19H30N2O2	(M+H)+
321.2457	321.2441	5.09	1	6451.66	C19H30N2O2	(M+H)+

--- End Of Report ---

Compound 24

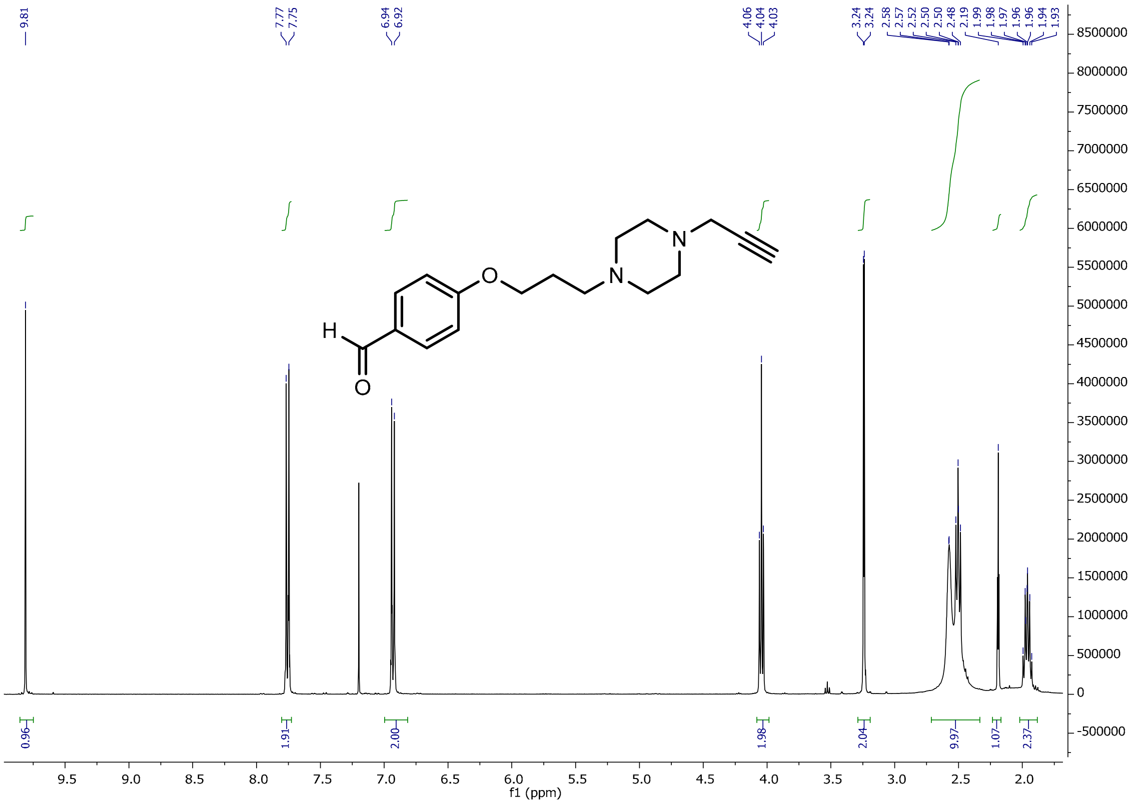

9.81
7.77
7.75
6.94
6.92
4.06
4.04
4.03
3.24
3.24
2.58
2.57
2.52
2.50
2.50
2.48
2.19
1.99
1.98
1.97
1.96
1.96
1.94
1.93
0.96
1.91
2.00
1.98
2.04
9.97
1.07
2.37
H
O
O
N
N
f1 (ppm)

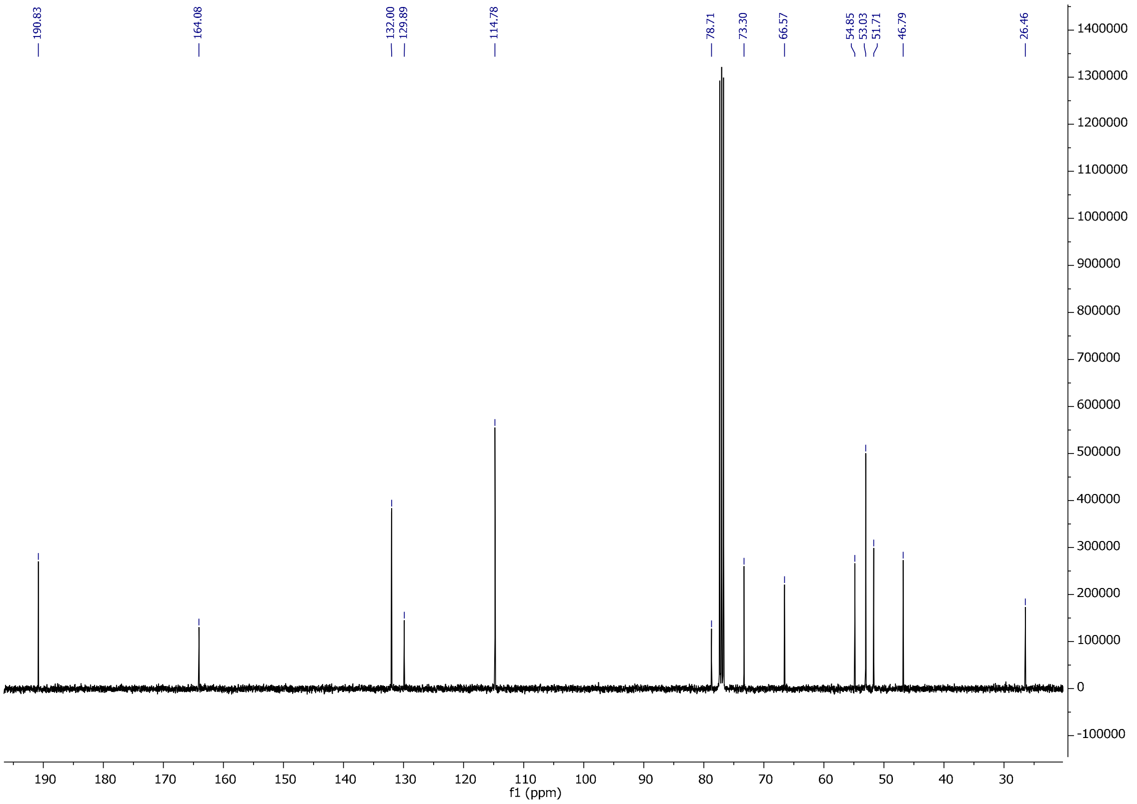

190.83
164.08
132.00
129.89
114.78
78.71
73.30
66.57
54.85
53.03
51.71
46.79
26.46
f1 (ppm)

Qualitative Compound Report

**Data File**	1525_DDI121_01.d	**Sample Name**	DDI121
**Sample Type**	Sample	**Position**	Vial 13
**Instrument Name**	Instrument 1	**User Name**	
**Acq Method**	ESI_ACN_75_pos_new.m	**Acquired Time**	6/9/2022 2:18:01 PM (UTC+02:00)
**IRM Calibration Status**	Some Ions Missed	**DA Method**	Defecto_modificado.m
**Comment**			

**Sample Group**		**Info.**	
**User**	MIREIA TOLEDANO	**Stream Name**	LC 1
**Acquisition Time (Local)**	6/9/2022 2:18:01 PM (UTC+02:00)	**Acquisition SW Version**	6200 series TOF/6500 series Q-TOF B.08.00 (B8058.3 SP1)
**QTOF Driver Version**	8.00.00	**QTOF Firmware Version**	2.712
**Tune Mass Range Max.**	3200		

Compound Table

Compound Label	RT	Mass	Abund	Formula	Tgt Mass	Diff (ppm)	Hits (DB)
Cpd 1: C17 H22 N2 O2; 2.553	2.553	286.1678	7835727	C17 H22 N2 O2	286.1681	-1.14	1

Compound Label	*m/z*	RT	Algorithm	Mass
Cpd 1: C17 H22 N2 O2; 2.553	287.1751	2.553	Find by Formula	286.1678

MS Zoomed Spectrum

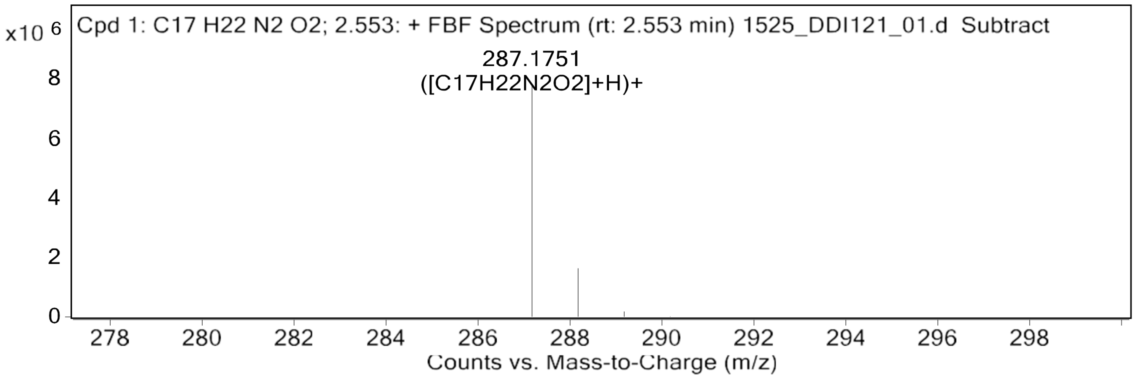

MS Spectrum Peak List

m/z	z	Abund	Formula	Ion
287.1751	1	7835726.5	C17H22N2O2	(M+H)+
288.178	1	1635233.88	C17H22N2O2	(M+H)+
289.1823	1	181302.19	C17H22N2O2	(M+H)+
290.1855	1	14643.4	C17H22N2O2	(M+H)+

MS Zoomed Spectrum

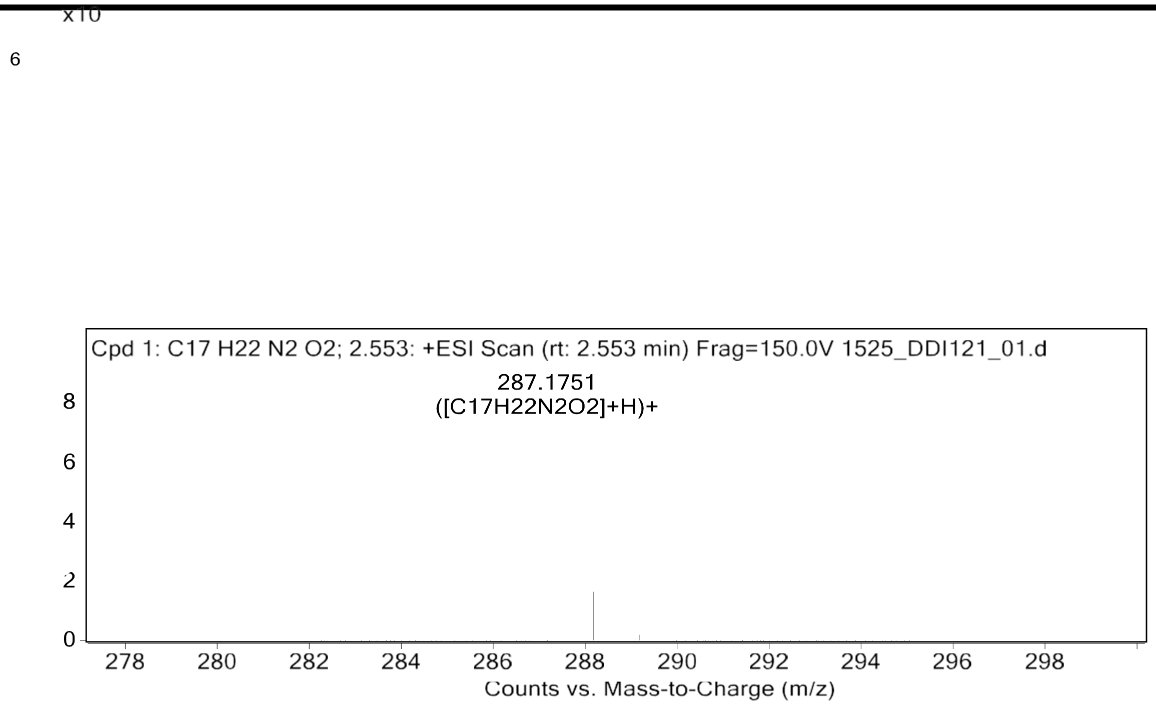

MS Spectrum Peak List

m/z	*Calc m/z*	Diff(ppm)	z	Abund	Formula	Ion
287.1751	287.1754	-1.08	1	7835726.5	C17H22N2O2	(M+H)+
288.178	288.1786	-1.91	1	1635233.88	C17H22N2O2	(M+H)+
289.1823	289.1813	3.23	1	181302.19	C17H22N2O2	(M+H)+
290.1855	290.184	5.27	1	14643.4	C17H22N2O2	(M+H)+

--- End Of Report ---

Nitrone 7

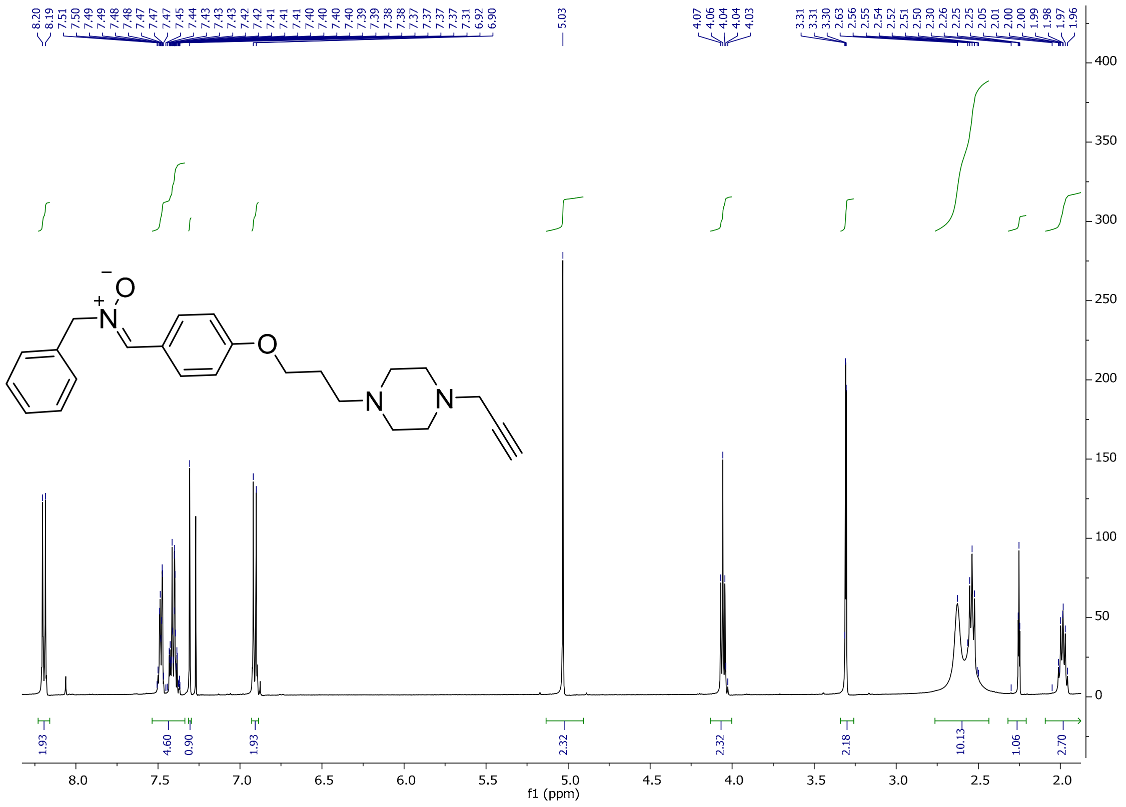

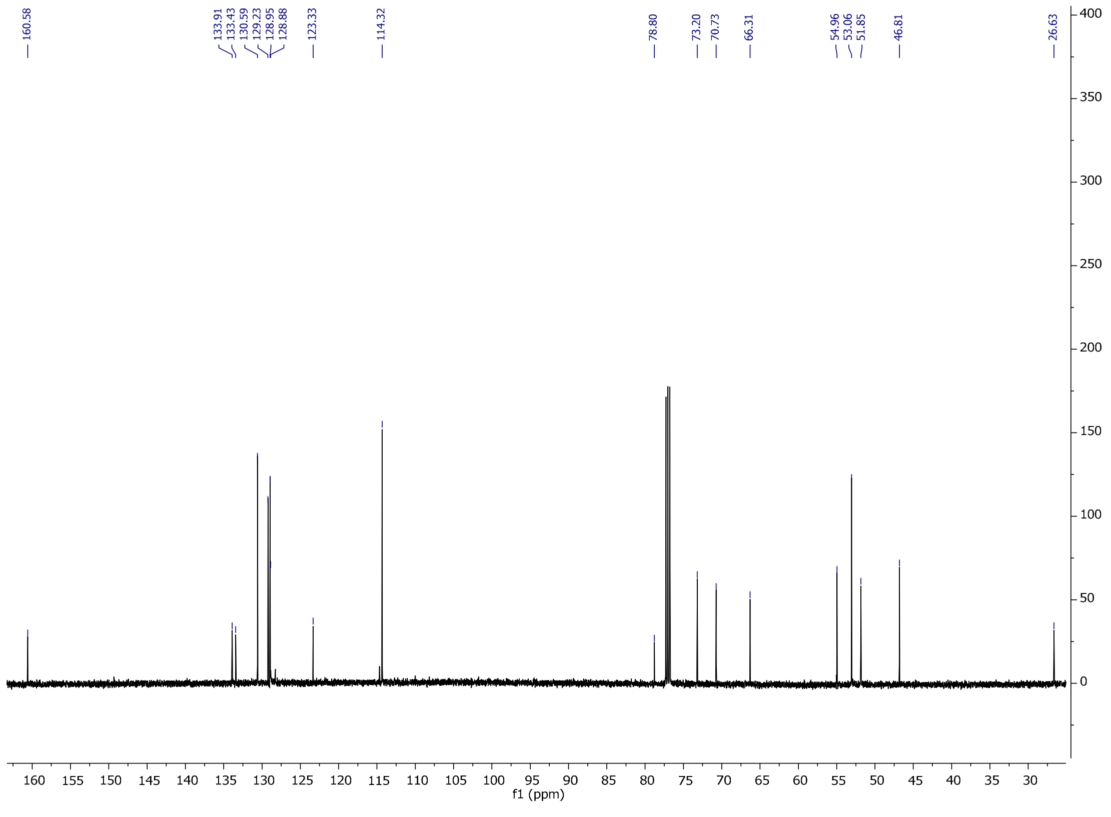

Qualitative Compound Report

**Data File Sample Type**	252_DDI123_02.d Sample	**Sample Name Position**	DDI123 Vial 6
**Instrument NameAcq Method**	Instrument 1 ESI_ACN_75_pos_new.m	**User Name Acquired Time**	2/19/2021 1:03:41 PM (UTC+01:00)
**IRM Calibration Status**	Success	**DA Method**	Defecto_modificado.m
**Comment**			

**Sample Group**		**Info.**	
**User**	DANIEL DIEZ	**Stream Name**	LC 1
**Acquisition Time (Local)**	2/19/2021 1:03:41 PM (UTC+01:00)	**Acquisition SW Version**	6200 series TOF/6500 series Q-TOF B.08.00 (B8058.3 SP1)
**QTOF Driver Version**	8.00.00	**QTOF Firmware Version**	2.712
**Tune Mass Range Max.**	1700		

Compound Table

Compound Label	RT	Mass	Abund	Formula	Tgt Mass	Diff (ppm)	Hits (DB)
Cpd 1: C24 H29 N3 O2; 0.279	0.279	391.2265	8876360	C24 H29 N3 O2	391.226	1.25	1

Compound Label	*m/z*	RT	Algorithm	Mass
Cpd 1: C24 H29 N3 O2; 0.279	392.2339	0.279	Find by Formula	391.2265

MS Zoomed Spectrum

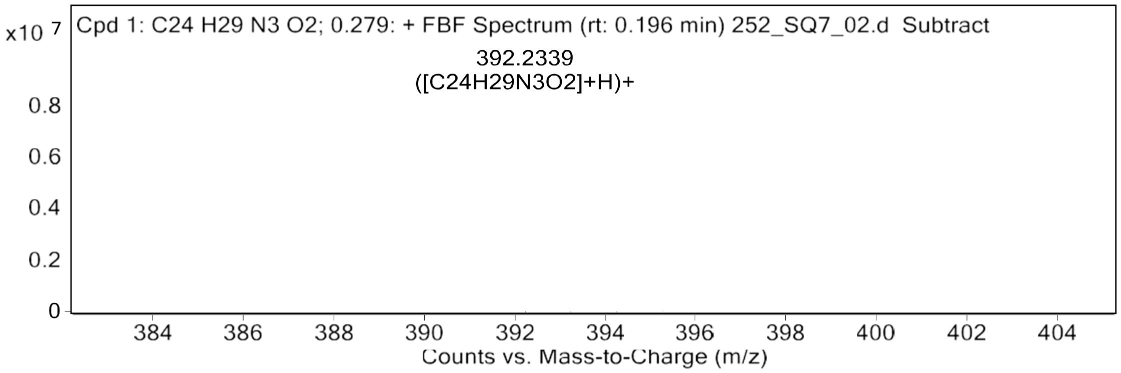

MS Spectrum Peak List

m/z	z	Abund	Formula	Ion
392.2339	1	8876360	C24H29N3O2	(M+H)+
393.2354	1	2633242.5	C24H29N3O2	(M+H)+
394.25	1	229347.34	C24H29N3O2	(M+H)+
395.2524	1	24608.16	C24H29N3O2	(M+H)+

MS Zoomed Spectrum

Qualitative Compound Report

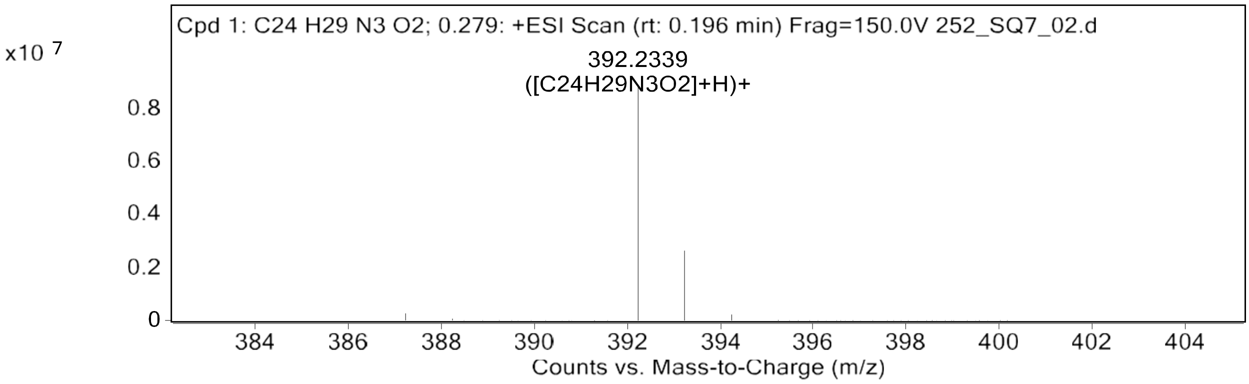

MS Spectrum Peak List

m/z	*Calc m/z*	Diff(ppm)	z	Abund	Formula	Ion
392.2339	392.2333	1.64	1	8876360	C24H29N3O2	(M+H)+
393.2354	393.2364	-2.57	1	2633242.5	C24H29N3O2	(M+H)+
394.25	394.2393	27.13	1	229347.34	C24H29N3O2	(M+H)+
395.2524	395.2421	26.13	1	24608.16	C24H29N3O2	(M+H)+

--- End Of Report ---

Compound 25

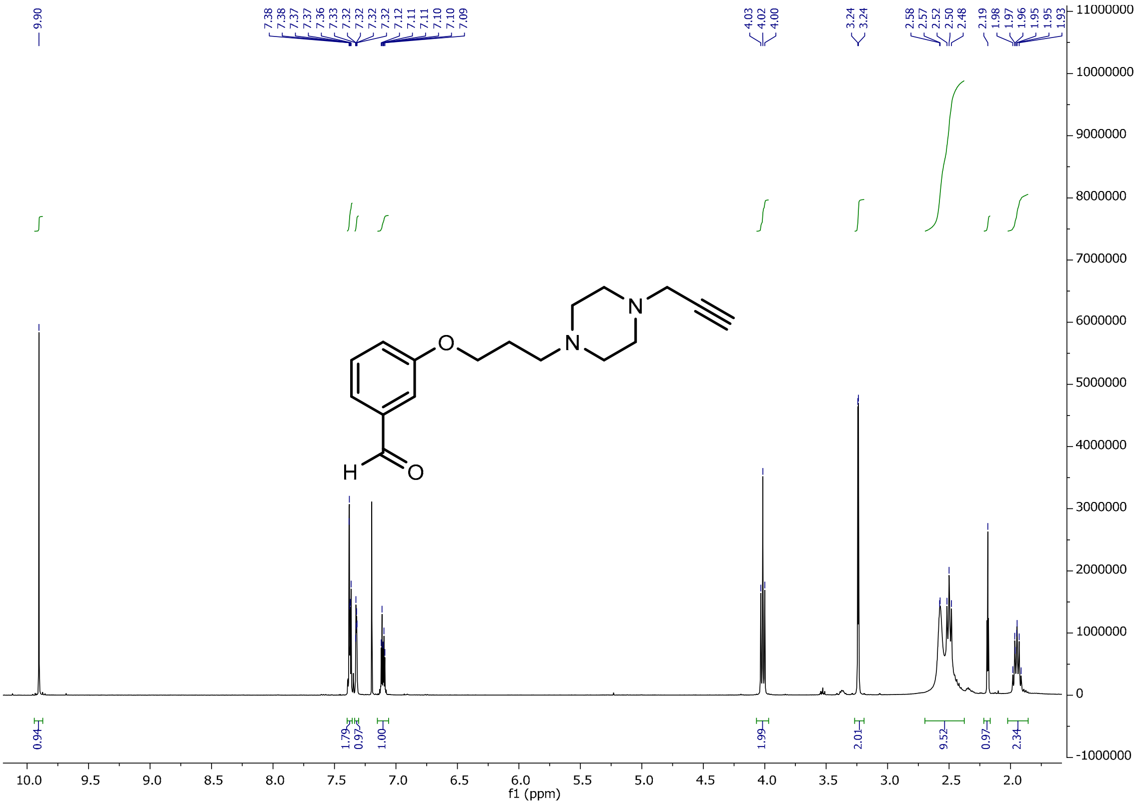

9.90
7.38 7.38 7.37 7.37 7.36 7.33 7.32 7.32 7.32 7.32 7.12 7.11 7.11 7.10 7.10 7.09
4.03 4.02 4.00
3.24 3.24
2.58 2.57 2.52 2.50 2.48
2.19 1.98 1.97 1.96 1.95 1.95 1.93
0.94 1.79 0.97 1.00 1.99 2.01 9.52 0.97 2.34
O N N H O
f1 (ppm)

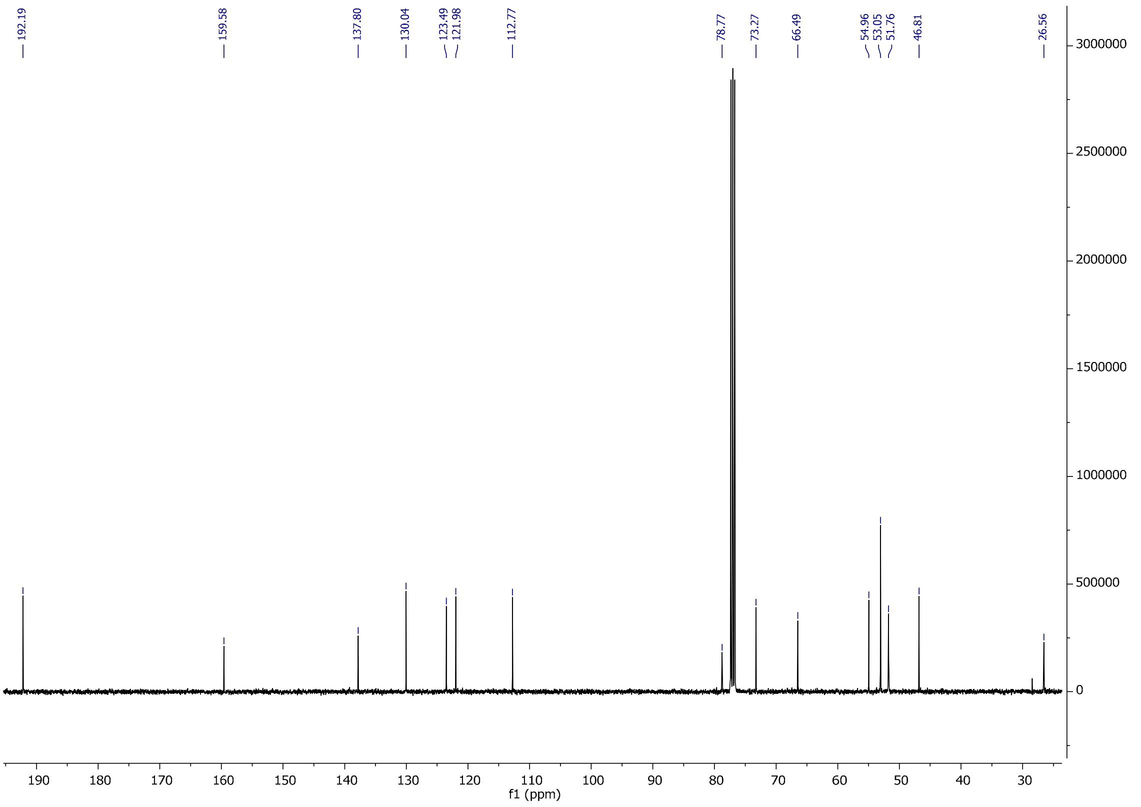

192.19 159.58 137.80 130.04 123.49 121.98 112.77 78.77 73.27 66.49 54.96 53.05 51.76 46.81 26.56
f1 (ppm)

Qualitative Compound Report

**Data File**	1528_DDI129_01.d	**Sample Name**	DDI129
**Sample Type**	Sample	**Position**	Vial 16
**Instrument Name**	Instrument 1	**User Name**	
**Acq Method**	ESI_ACN_75_pos_new.m	**Acquired Time**	6/9/2022 2:43:10 PM (UTC+02:00)
**IRM Calibration Status**	Success	**DA Method**	Defecto_modificado.m
**Comment**			

**Sample Group**		**Info.**	
**User**	MIREIA TOLEDANO	**Stream Name**	LC 1
**Acquisition Time (Local)**	6/9/2022 2:43:10 PM (UTC+02:00)	**Acquisition SW Version**	6200 series TOF/6500 series Q-TOF B.08.00 (B8058.3 SP1)
**QTOF Driver Version**	8.00.00	**QTOF Firmware Version**	2.712
**Tune Mass Range Max.**	3200		

Compound Table

Compound Label	RT	Mass	Abund	Formula	Tgt Mass	Diff (ppm)	Hits (DB)
Cpd 1: C17 H22 N2 O2; 2.644	2.644	286.1684	947507	C17 H22 N2 O2	286.1681	0.79	1

Compound Label	*m/z*	RT	Algorithm	Mass
Cpd 1: C17 H22 N2 O2; 2.644	287.1757	2.644	Find by Formula	286.1684

MS Zoomed Spectrum

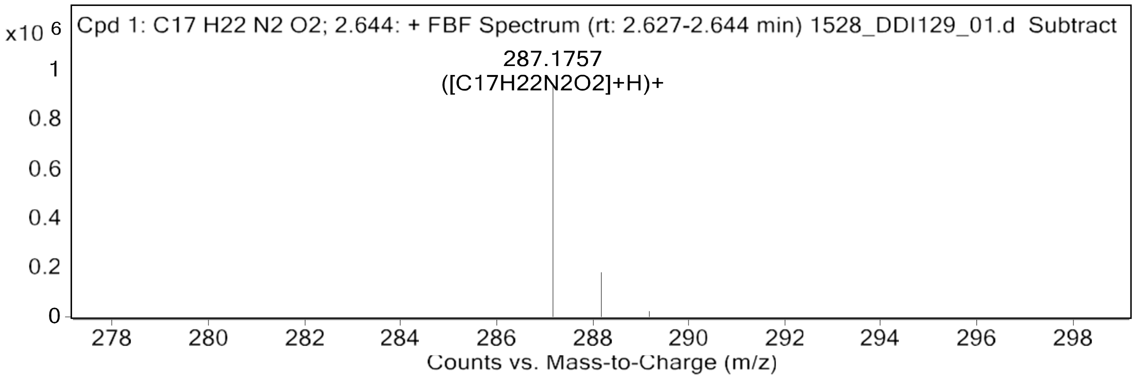

MS Spectrum Peak List

m/z	z	Abund	Formula	Ion
287.1757	1	947507.13	C17H22N2O2	(M+H)+
288.1786	1	181392.98	C17H22N2O2	(M+H)+
289.1808	1	23212.05	C17H22N2O2	(M+H)+

MS Zoomed Spectrum

Qualitative Compound Report

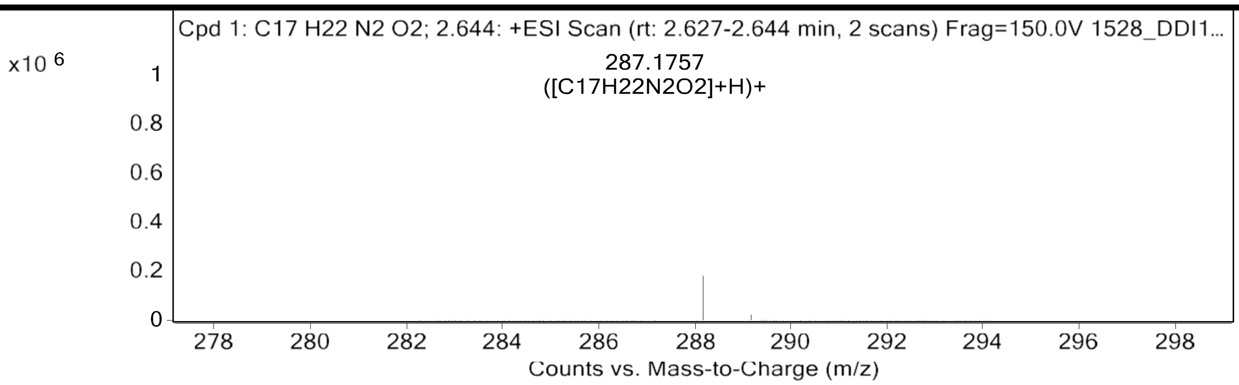

MS Spectrum Peak List

m/z	*Calc m/z*	Diff(ppm)	z	Abund	Formula	Ion
287.1757	287.1754	1.02	1	947507.13	C17H22N2O2	(M+H)+
288.1786	288.1786	-0.05	1	181392.98	C17H22N2O2	(M+H)+
289.1808	289.1813	-1.88	1	23212.05	C17H22N2O2	(M+H)+

--- End Of Report ---

Nitrone 9

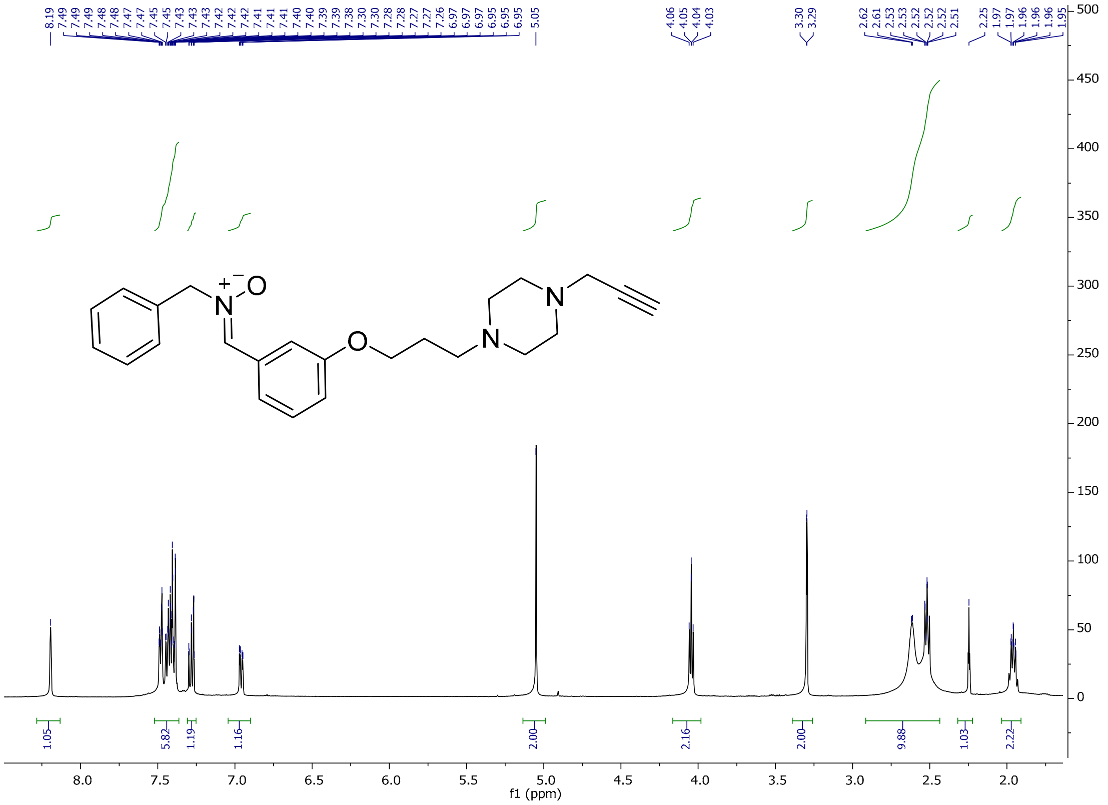

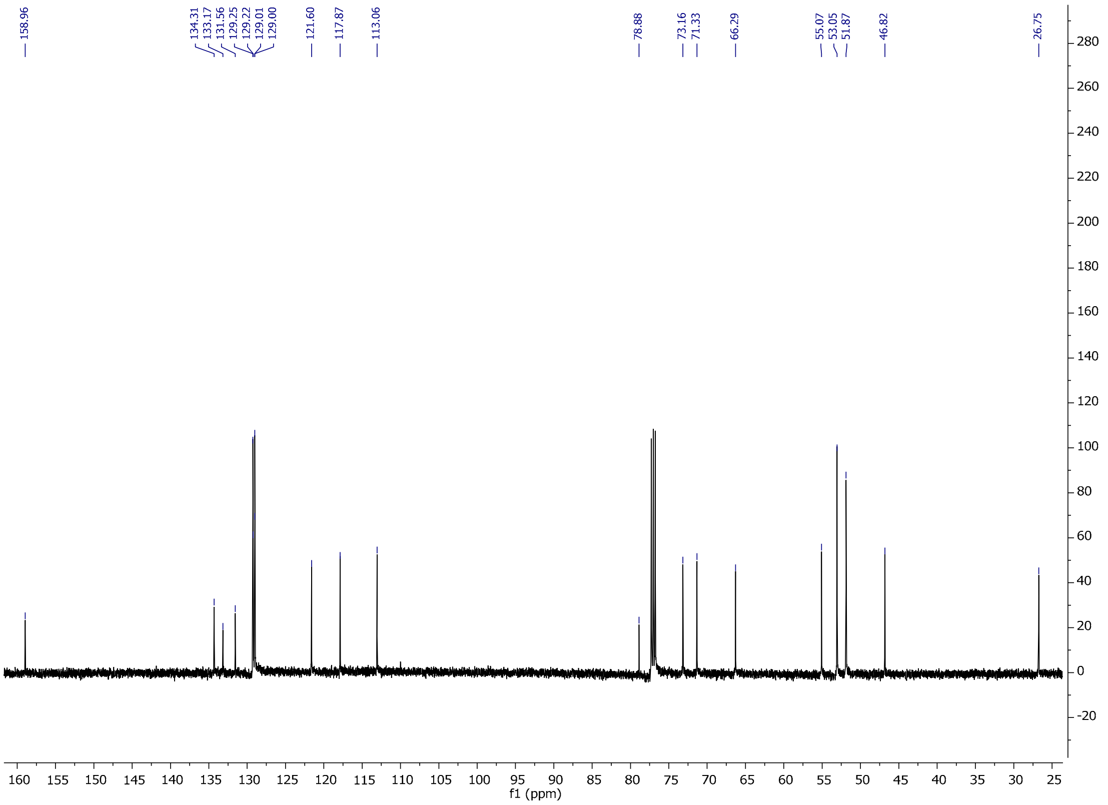

Qualitative Compound Report

**Data File**	511_DDI136_01.d	**Sample Name**	DDI136
**Sample Type**	Sample	**Position**	Vial 4
**Instrument Name**	Instrument 1	**User Name**	
**Acq Method**	ESI_ACN_75_pos_new.m	**Acquired Time**	5/27/2021 11:57:45 AM (UTC+01:00)
**IRM Calibration Status**	Success	**DA Method**	Defecto_modificado.m
**Comment**			

**Sample Group**		**Info.**	
**User**	DANIEL DIEZ	**Stream Name**	LC 1
**Acquisition Time (Local)**	5/27/2021 11:57:45 AM (UTC+01:00)	**Acquisition SW Version**	6200 series TOF/6500 series Q-TOF B.08.00 (B8058.3 SP1)
**QTOF Driver Version**	8.00.00	**QTOF Firmware Version**	2.712
**Tune Mass Range Max.**	1700		

Compound Table

Compound Label	RT	Mass	Abund	Formula	Tgt Mass	Diff (ppm)	Hits (DB)
Cpd 1: C24 H29 N3 O2; 1.635	1.635	391.2266	283950	C24 H29 N3 O2	391.226	1.57	1

Compound Label	*m/z*	RT	Algorithm	Mass
Cpd 1: C24 H29 N3 O2; 1.635	392.2339	1.635	Find by Formula	391.2266

MS Zoomed Spectrum

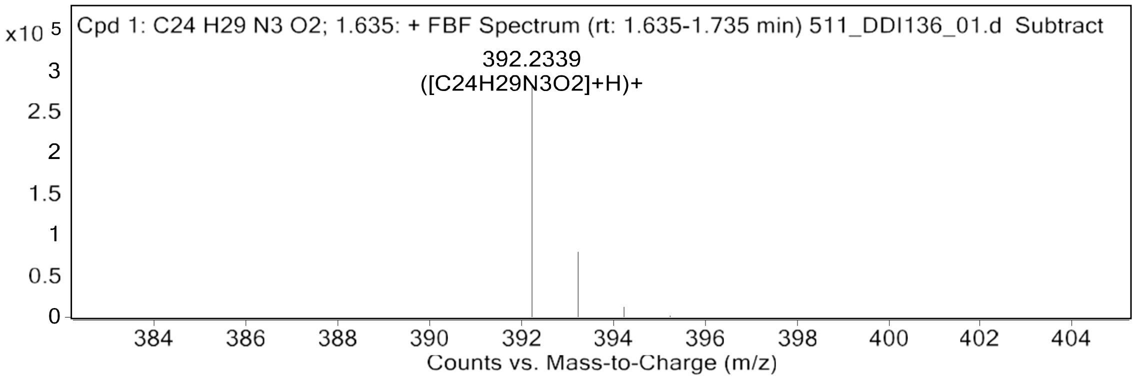

MS Spectrum Peak List

m/z	z	Abund	Formula	Ion
392.2339	1	283949.53	C24H29N3O2	(M+H)+
393.2371	1	79681.7	C24H29N3O2	(M+H)+
394.2398	1	12521.57	C24H29N3O2	(M+H)+
395.242	1	1705.5	C24H29N3O2	(M+H)+

MS Zoomed Spectrum

Qualitative Compound Report

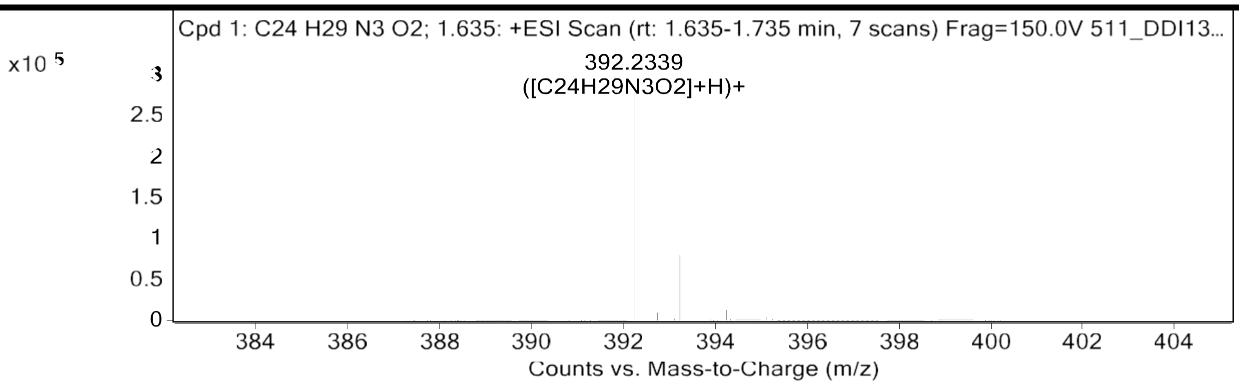

MS Spectrum Peak List

m/z	*Calc m/z*	Diff(ppm)	z	Abund	Formula	Ion
392.2339	392.2333	1.54	1	283949.53	C24H29N3O2	(M+H)+
393.2371	393.2364	1.72	1	79681.7	C24H29N3O2	(M+H)+
394.2398	394.2393	1.18	1	12521.57	C24H29N3O2	(M+H)+
395.242	395.2421	-0.35	1	1705.5	C24H29N3O2	(M+H)+

--- End Of Report ---

Compound 26

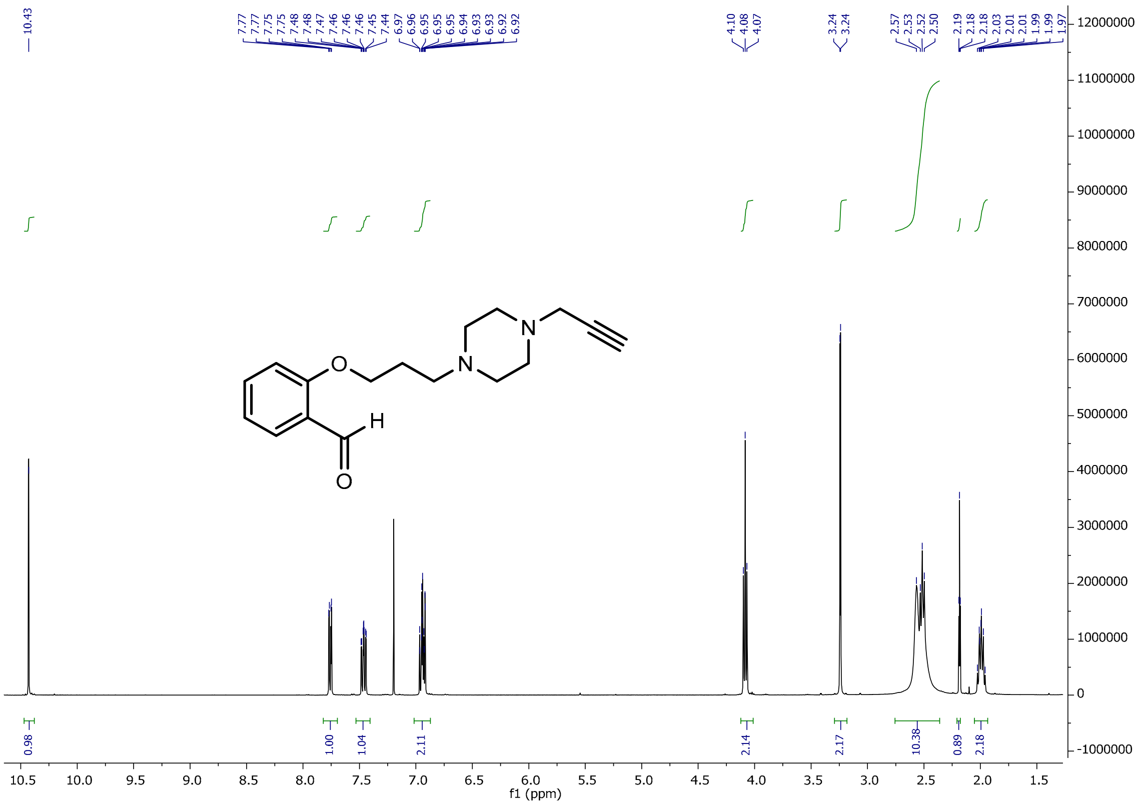

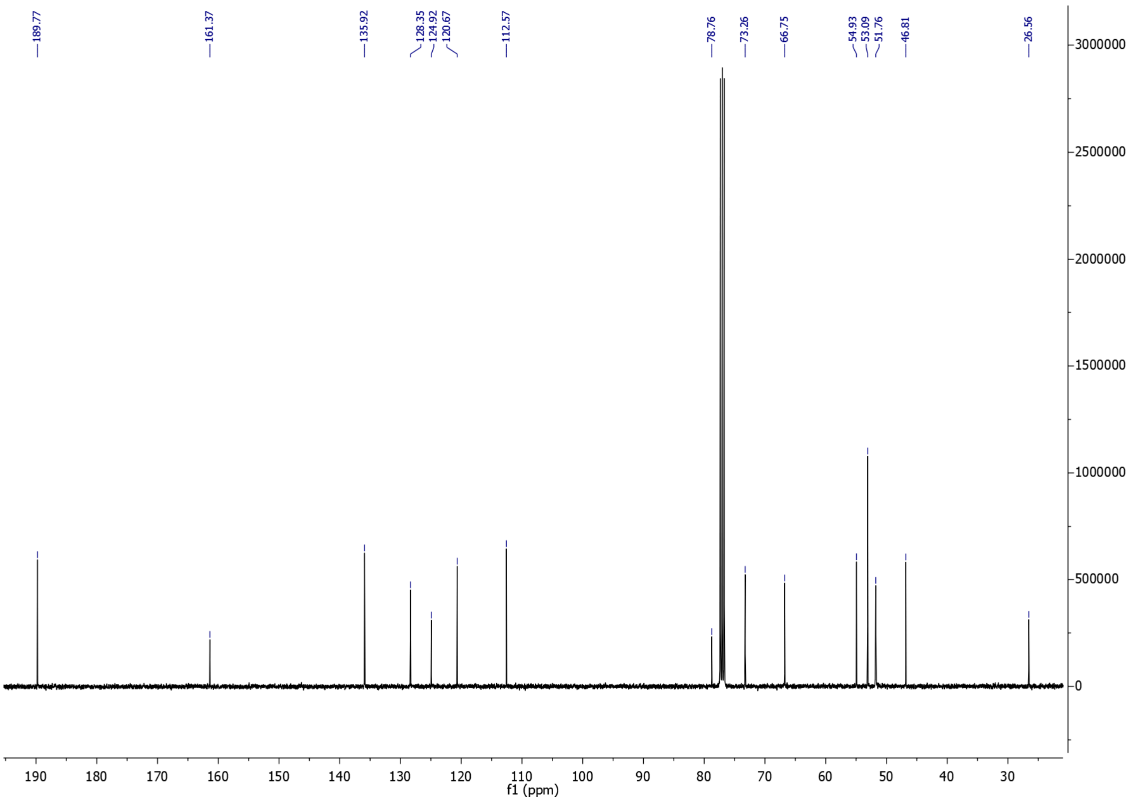

Qualitative Compound Report

**Data File**	253_DDI134_01_.d	**Sample Name**	DDI134
**Sample Type**	Sample	**Position**	Vial 41
**Instrument Name**	Instrument 1	**User Name**	
**Acq Method**	ESI_ACN_75_blanco_pos_new.m	**Acquired Time**	2/19/2021 11:47:50 AM (UTC+01:00)
**IRM Calibration Status**	Success	**DA Method**	Defecto_modificado.m
**Comment**			

**Sample Group**		**Info.**	
**User**	EHF	**Stream Name**	LC 1
**Acquisition Time (Local)**	2/19/2021 11:47:50 AM (UTC+01:00)	**Acquisition SW Version**	6200 series TOF/6500 series Q-TOF B.08.00 (B8058.3 SP1)
**QTOF Driver Version**	8.00.00	**QTOF Firmware Version**	2.712
**Tune Mass Range Max.**	1700		

Compound Table

Compound Label	RT	Mass	Abund	Formula	Tgt Mass	Diff (ppm)	Hits (DB)
Cpd 1: C17 H22 N2 O2; 1.338	1.338	286.1685	307780	C17 H22 N2 O2	286.1681	1.45	1

Compound Label	*m/z*	RT	Algorithm	Mass
Cpd 1: C17 H22 N2 O2; 1.338	287.1757	1.338	Find by Formula	286.1685

MS Zoomed Spectrum

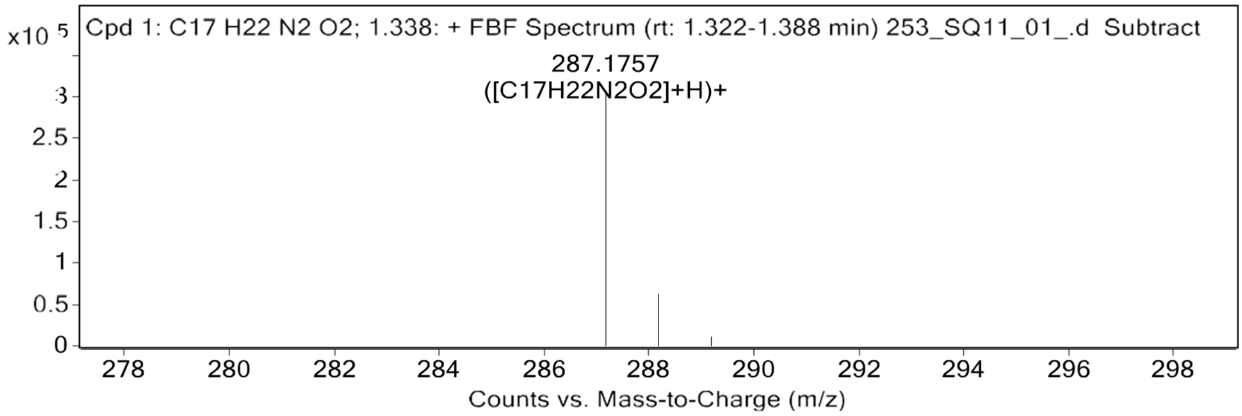

MS Spectrum Peak List

m/z	z	Abund	Formula	Ion
287.1757	1	307780.47	C17H22N2O2	(M+H)+
288.1787	1	63169.23	C17H22N2O2	(M+H)+
289.187	1	11117.15	C17H22N2O2	(M+H)+

MS Zoomed Spectrum

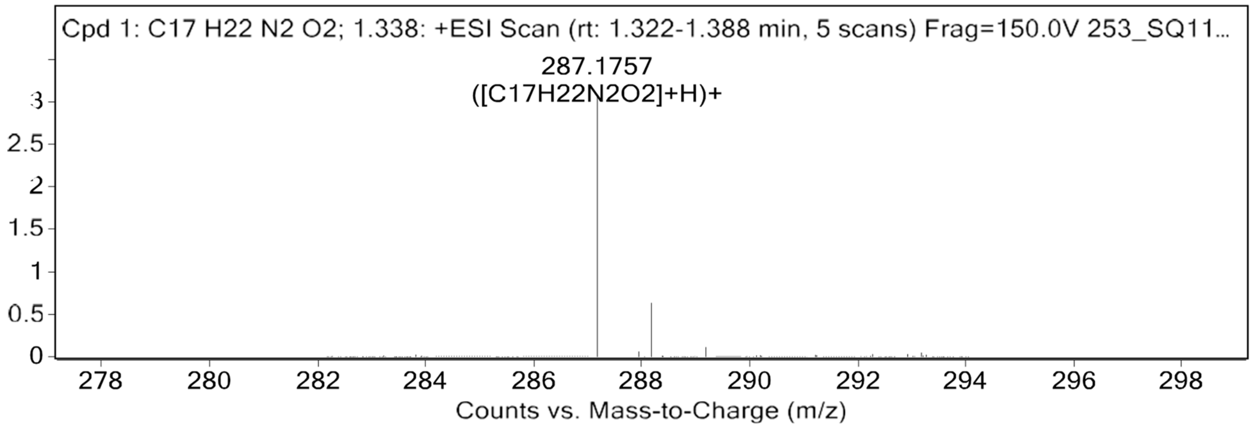

MS Spectrum Peak List

m/z	*Calc m/z*	Diff(ppm)	z	Abund	Formula	Ion
287.1757	287.1754	0.96	1	307780.47	C17H22N2O2	(M+H)+
288.1787	288.1786	0.55	1	63169.23	C17H22N2O2	(M+H)+
289.187	289.1813	19.69	1	11117.15	C17H22N2O2	(M+H)+

--- End Of Report ---

Nitrone 11

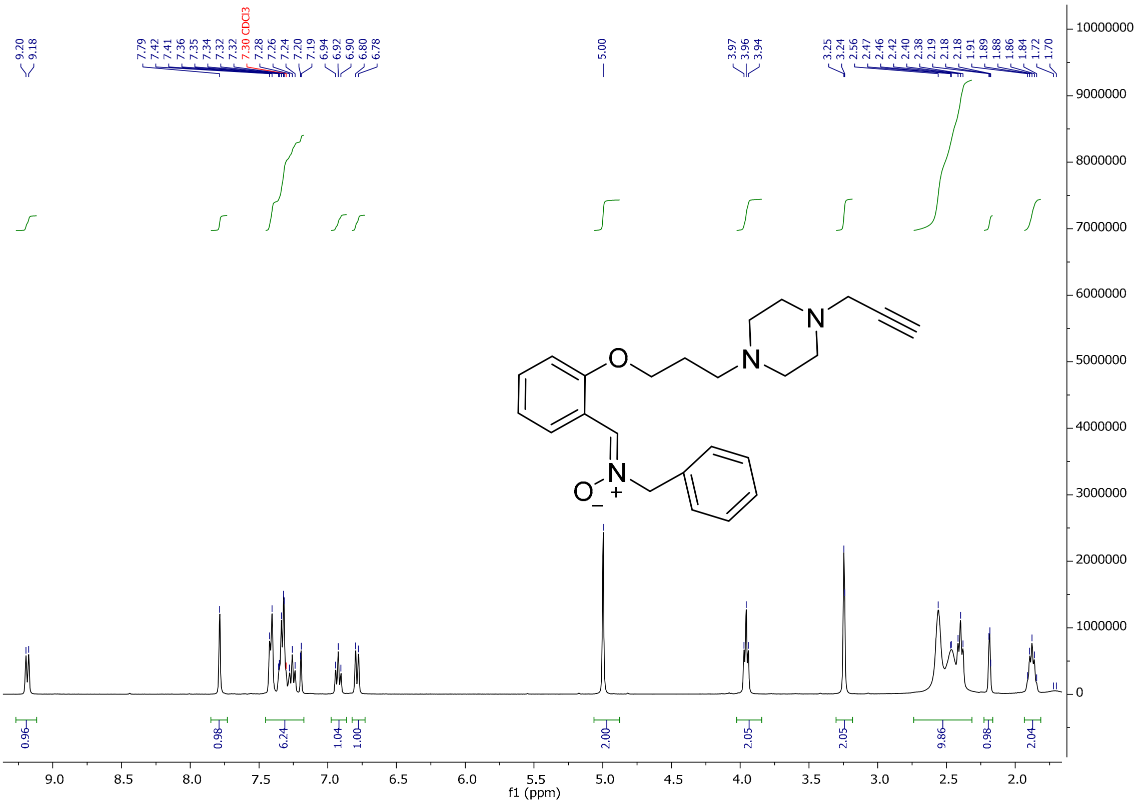

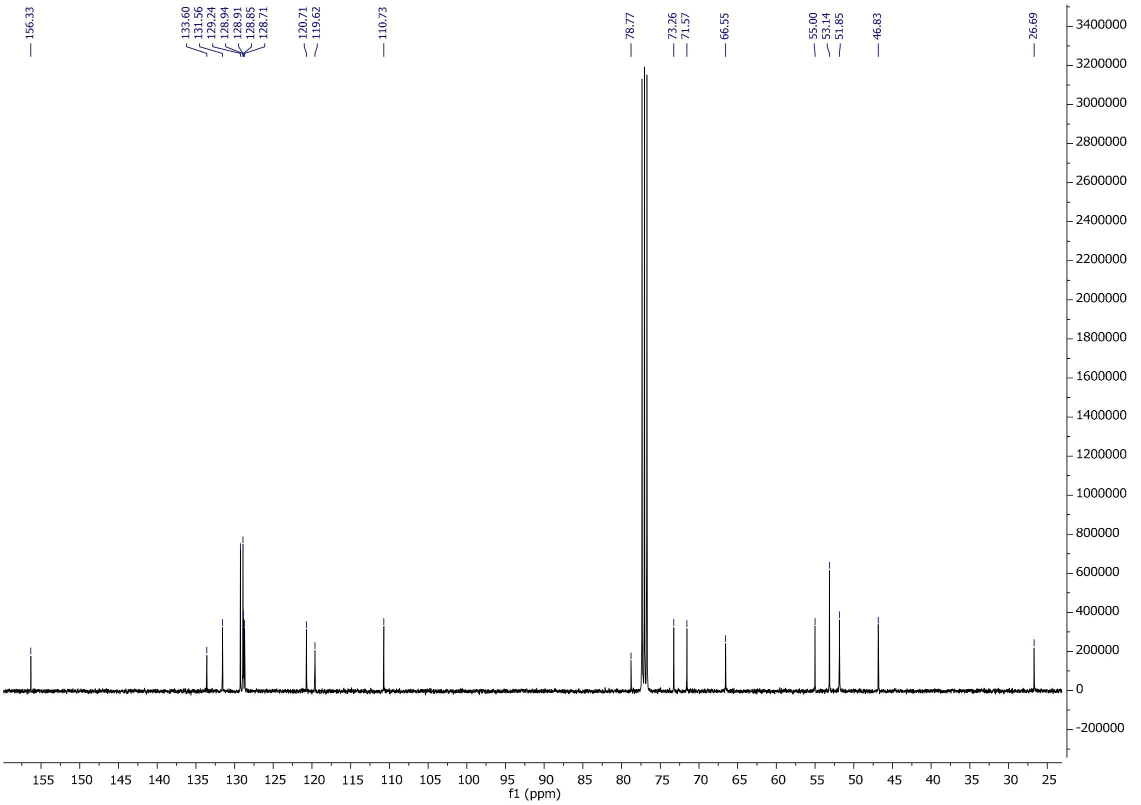

Qualitative Compound Report

**Data File**	510_DDI135_01.d	**Sample Name**	DDI135
**Sample Type**	Sample	**Position**	Vial 3
**Instrument Name**	Instrument 1	**User Name**	
**Acq Method**	ESI_ACN_75_pos_new.m	**Acquired Time**	5/27/2021 11:50:33 AM (UTC+01:00)
**IRM Calibration Status**	Success	**DA Method**	Defecto_modificado.m
**Comment**			

**Sample Group**		**Info.**	
**User**	DANIEL DIEZ	**Stream Name**	LC 1
**Acquisition Time (Local)**	5/27/2021 11:50:33 AM (UTC+01:00)	**Acquisition SW Version**	6200 series TOF/6500 series Q-TOF B.08.00 (B8058.3 SP1)
**QTOF Driver Version**	8.00.00	**QTOF Firmware Version**	2.712
**Tune Mass Range Max.**	1700		

Compound Table

Compound Label	RT	Mass	Abund	Formula	Tgt Mass	Diff (ppm)	Hits (DB)
Cpd 1: C24 H29 N3 O2; 1.668	1.668	391.2266	279258	C24 H29 N3 O2	391.226	1.5	1

Compound Label	*m/z*	RT	Algorithm	Mass
Cpd 1: C24 H29 N3 O2; 1.668	392.2338	1.668	Find by Formula	391.2266

MS Zoomed Spectrum

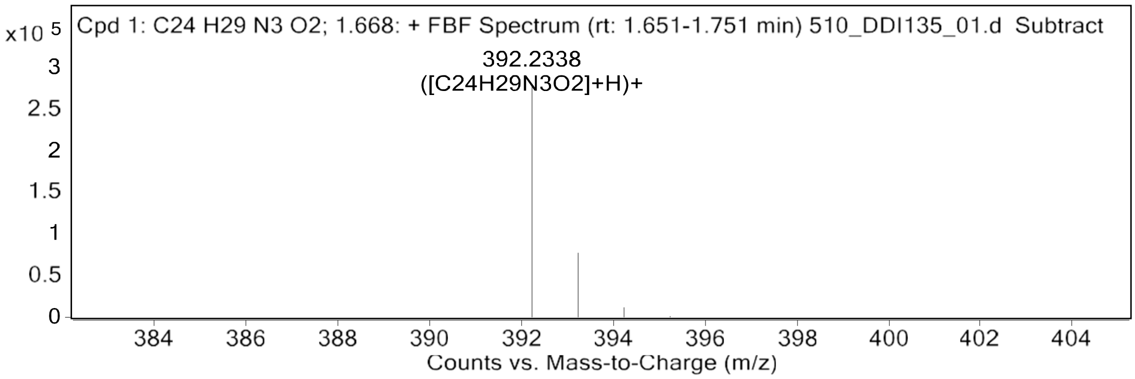

MS Spectrum Peak List

m/z	z	Abund	Formula	Ion
392.2338	1	279258	C24H29N3O2	(M+H)+
393.237	1	77398.37	C24H29N3O2	(M+H)+
394.2403	1	11758.92	C24H29N3O2	(M+H)+
395.2422	1	1461.8	C24H29N3O2	(M+H)+

MS Zoomed Spectrum

Qualitative Compound Report

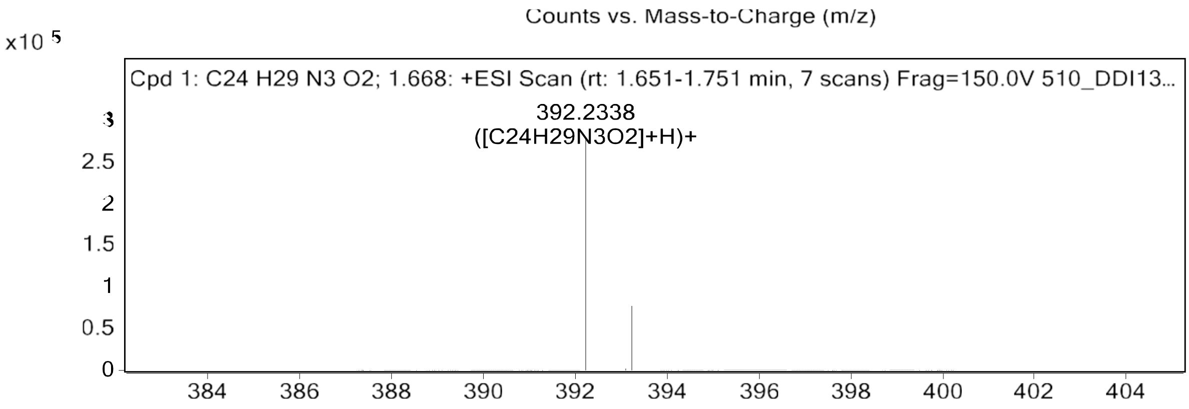

MS Spectrum Peak List

m/z	*Calc m/z*	Diff(ppm)	z	Abund	Formula	Ion
392.2338	392.2333	1.46	1	279258	C24H29N3O2	(M+H)+
393.237	393.2364	1.49	1	77398.37	C24H29N3O2	(M+H)+
394.2403	394.2393	2.43	1	11758.92	C24H29N3O2	(M+H)+
395.2422	395.2421	0.29	1	1461.8	C24H29N3O2	(M+H)+

--- End Of Report ---

Nitrone 8

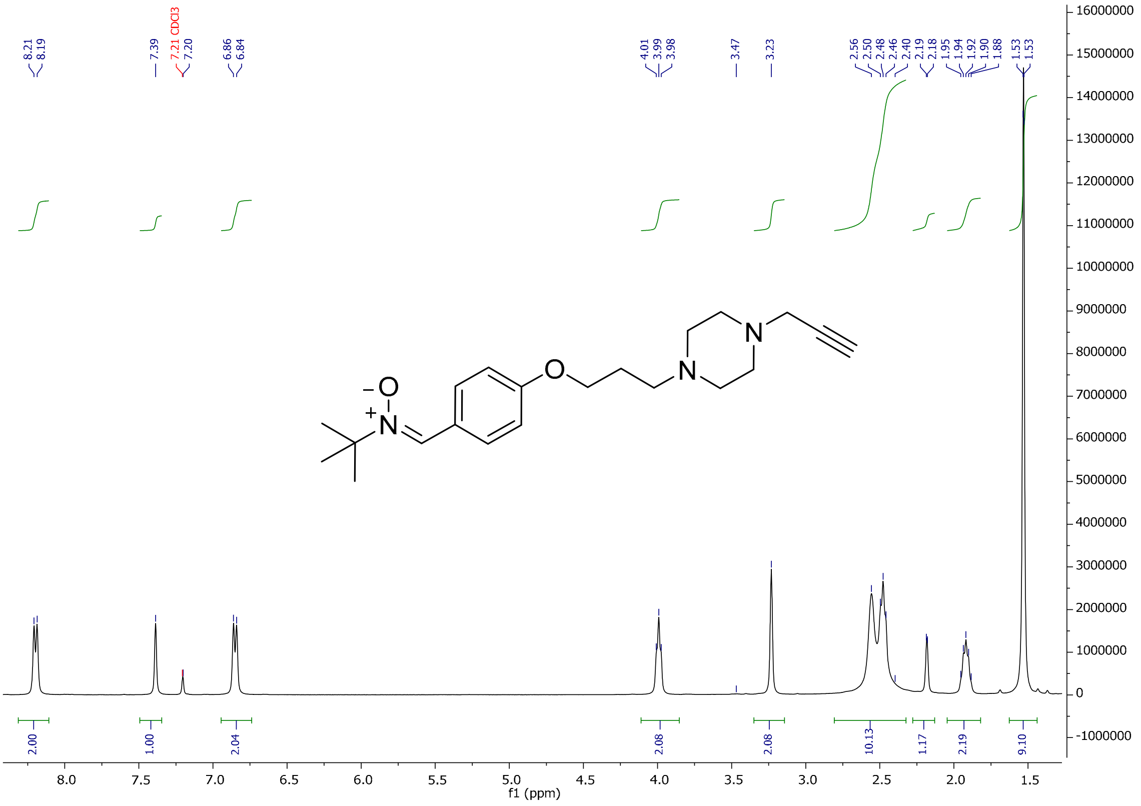

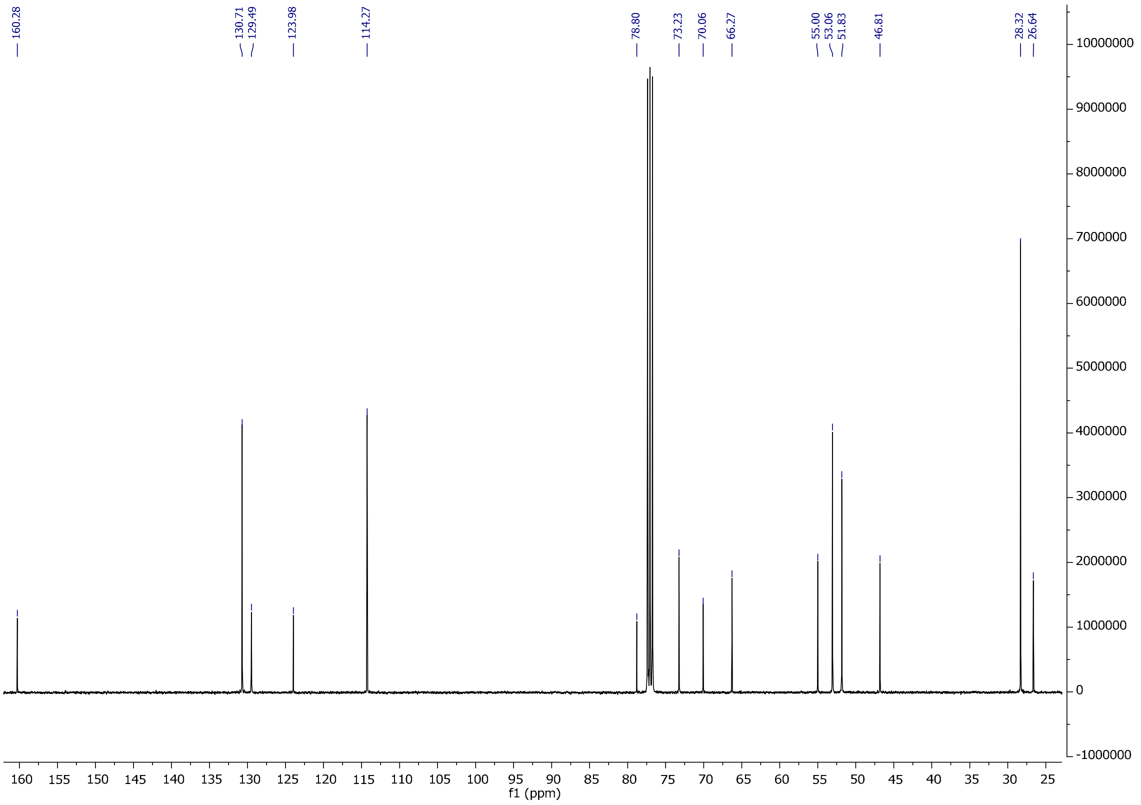

Qualitative Compound Report

**Data File**	508_DDI124_01.d	**Sample Name**	DDI124
**Sample Type**	Sample	**Position**	Vial 1
**Instrument Name**	Instrument 1	**User Name**	
**Acq Method**	ESI_ACN_75_pos_new.m	**Acquired Time**	5/27/2021 11:19:21 AM (UTC+01:00)
**IRM Calibration Status**	Success	**DA Method**	Defecto_modificado.m
**Comment**			

**Sample Group**		**Info.**	
**User**	DANIEL DIEZ	**Stream Name**	LC 1
**Acquisition Time (Local)**	5/27/2021 11:19:21 AM (UTC+01:00)	**Acquisition SW Version**	6200 series TOF/6500 series Q-TOF B.08.00 (B8058.3 SP1)
**QTOF Driver Version**	8.00.00	**QTOF Firmware Version**	2.712
**Tune Mass Range Max.**	1700		

Compound Table

Compound Label	RT	Mass	Abund	Formula	Tgt Mass	Diff (ppm)	Hits (DB)
Cpd 1: C21 H31 N3 O2; 2.341	2.341	357.2416	207448	C21 H31 N3 O2	357.2416	-0.04	1

Compound Label	*m/z*	RT	Algorithm	Mass
Cpd 1: C21 H31 N3 O2; 2.341	358.2489	2.341	Find by Formula	357.2416

MS Zoomed Spectrum

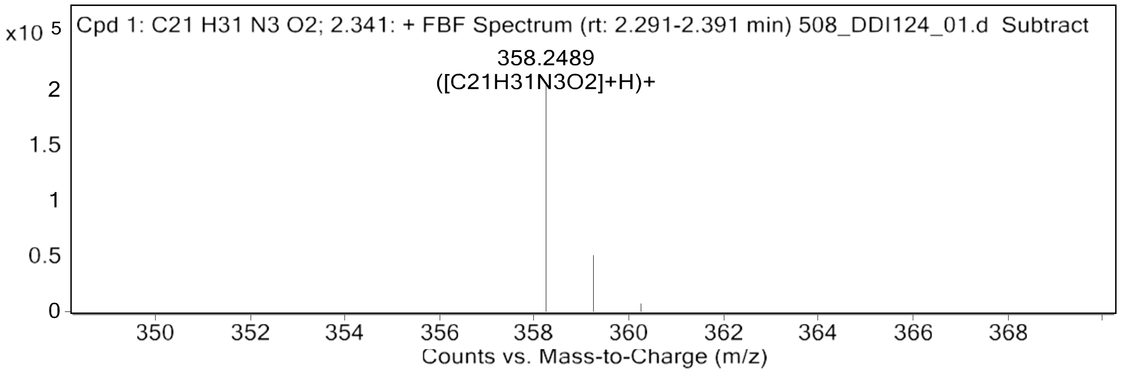

MS Spectrum Peak List

m/z	z	Abund	Formula	Ion
358.2489	1	207447.58	C21H31N3O2	(M+H)+
359.252	1	51090.4	C21H31N3O2	(M+H)+
360.2536	1	7453.08	C21H31N3O2	(M+H)+

MS Zoomed Spectrum

Qualitative Compound Report

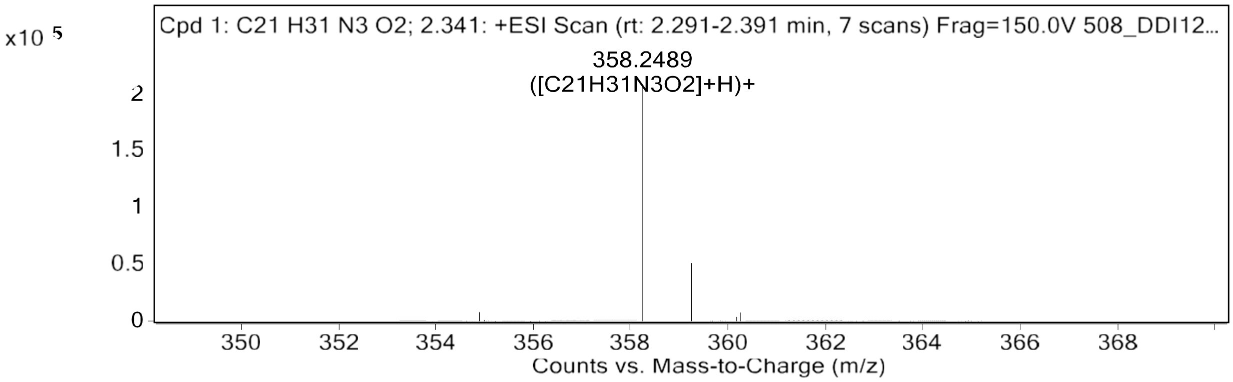

MS Spectrum Peak List

m/z	*Calc m/z*	Diff(ppm)	z	Abund	Formula	Ion
358.2489	358.2489	0.11	1	207447.58	C21H31N3O2	(M+H)+
359.252	359.252	-0.13	1	51090.4	C21H31N3O2	(M+H)+
360.2536	360.2549	-3.65	1	7453.08	C21H31N3O2	(M+H)+

--- End Of Report ---

Nitrone 10

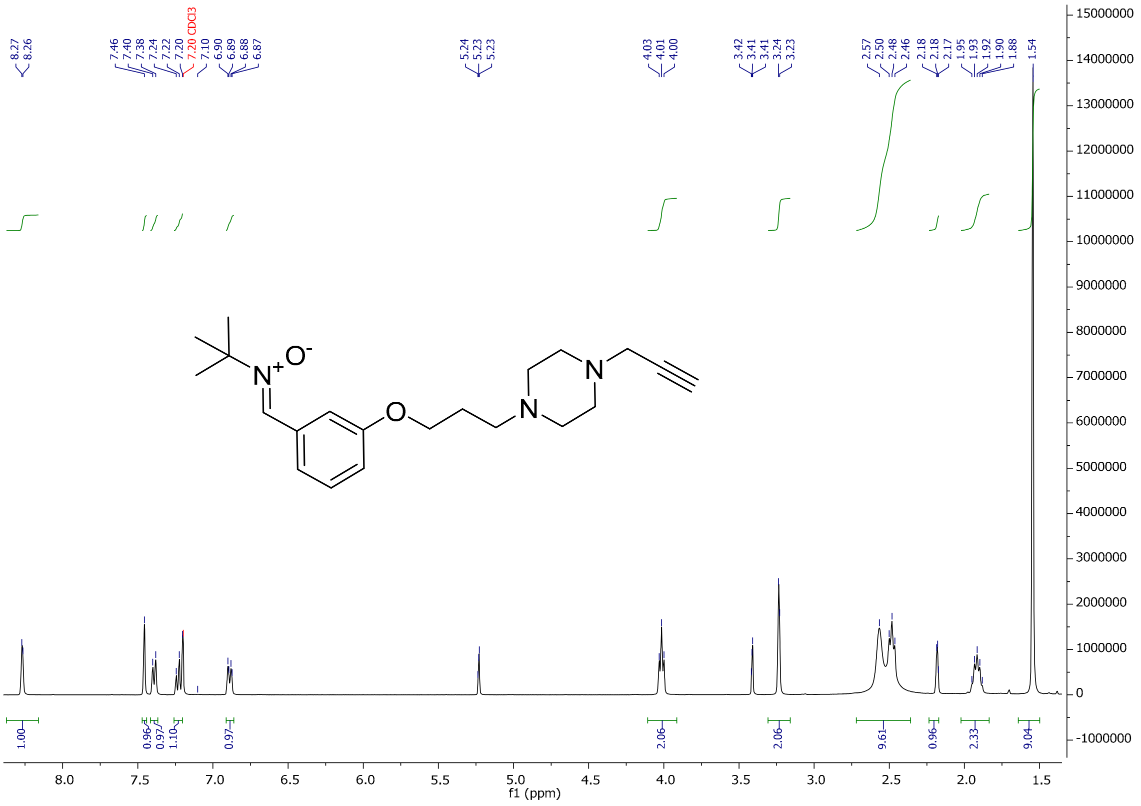

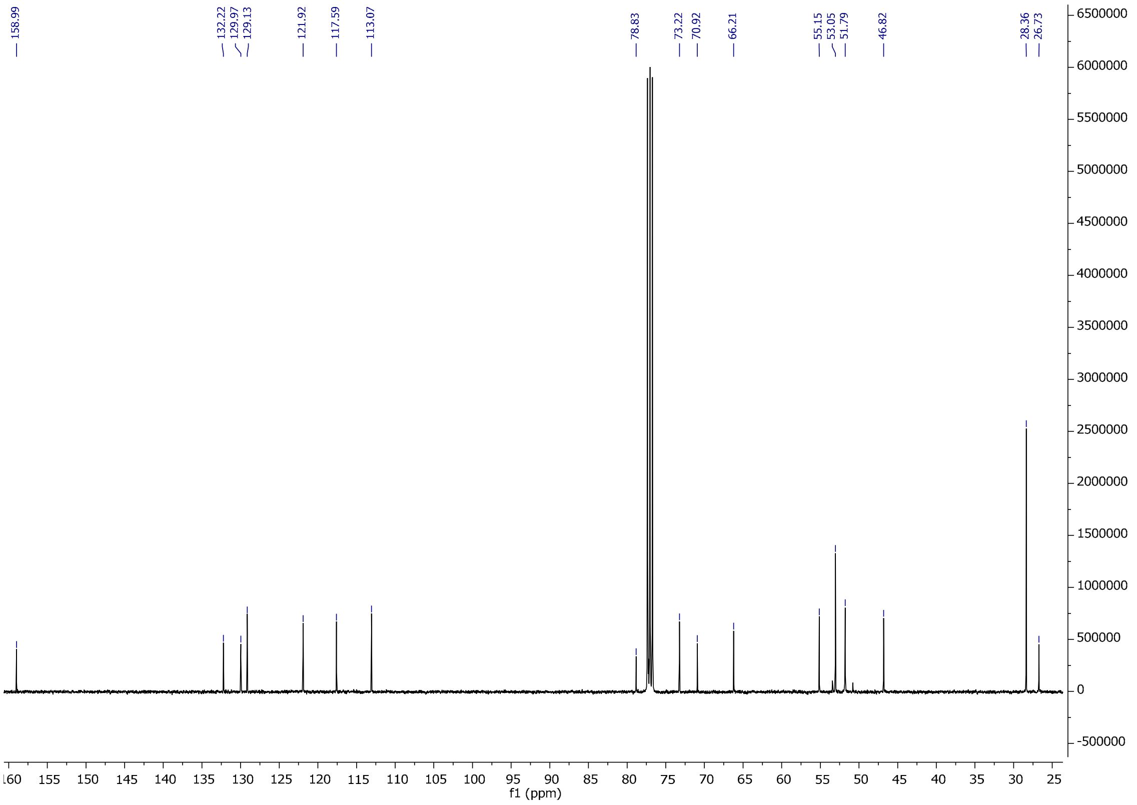

Qualitative Compound Report

**Data File**	516_DDI146_01.d	**Sample Name**	DDI146
**Sample Type**	Sample	**Position**	Vial 9
**Instrument Name**	Instrument 1	**User Name**	
**Acq Method**	ESI_ACN_75_pos_new.m	**Acquired Time**	5/27/2021 1:06:17 PM (UTC+01:00)
**IRM Calibration Status**	Success	**DA Method**	Defecto_modificado.m
**Comment**			

**Sample Group**		**Info.**	
**User**	DANIEL DIEZ	**Stream Name**	LC 1
**Acquisition Time (Local)**	5/27/2021 1:06:17 PM (UTC+01:00)	**Acquisition SW Version**	6200 series TOF/6500 series Q-TOF B.08.00 (B8058.3 SP1)
**QTOF Driver Version**	8.00.00	**QTOF Firmware Version**	2.712
**Tune Mass Range Max.**	1700		

Compound Table

Compound Label	RT	Mass	Abund	Formula	Tgt Mass	Diff (ppm)	Hits (DB)
Cpd 1: C21 H31 N3 O2; 1.043	1.043	357.2416	9813700	C21 H31 N3 O2	357.2416	-0.11	1

Compound Label	*m/z*	RT	Algorithm	Mass
Cpd 1: C21 H31 N3 O2; 1.043	358.2488	1.043	Find by Formula	357.2416

MS Zoomed Spectrum

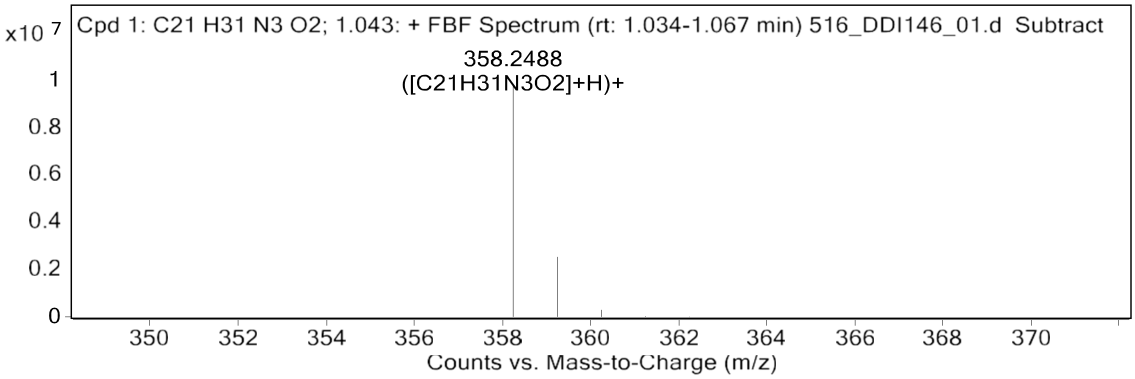

MS Spectrum Peak List

m/z	z	Abund	Formula	Ion
358.2488	1	9813700	C21H31N3O2	(M+H)+
359.2521	1	2534226.25	C21H31N3O2	(M+H)+
360.2569	1	295964.38	C21H31N3O2	(M+H)+
361.2593	1	28935.77	C21H31N3O2	(M+H)+
362.2443	1	2363.16	C21H31N3O2	(M+H)+

MS Zoomed Spectrum

Qualitative Compound Report

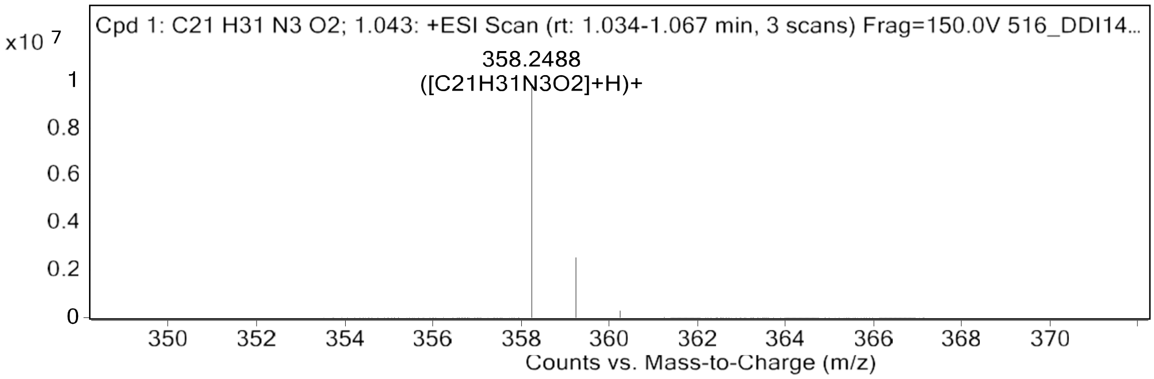

MS Spectrum Peak List

m/z	*Calc m/z*	Diff(ppm)	z	Abund	Formula	Ion
358.2488	358.2489	-0.34	1	9813700	C21H31N3O2	(M+H)+
359.2521	359.252	0.09	1	2534226.25	C21H31N3O2	(M+H)+
360.2569	360.2549	5.56	1	295964.38	C21H31N3O2	(M+H)+
361.2593	361.2576	4.71	1	28935.77	C21H31N3O2	(M+H)+
362.2443	362.2603	-43.96	1	2363.16	C21H31N3O2	(M+H)+

--- End Of Report ---

Nitrone 12

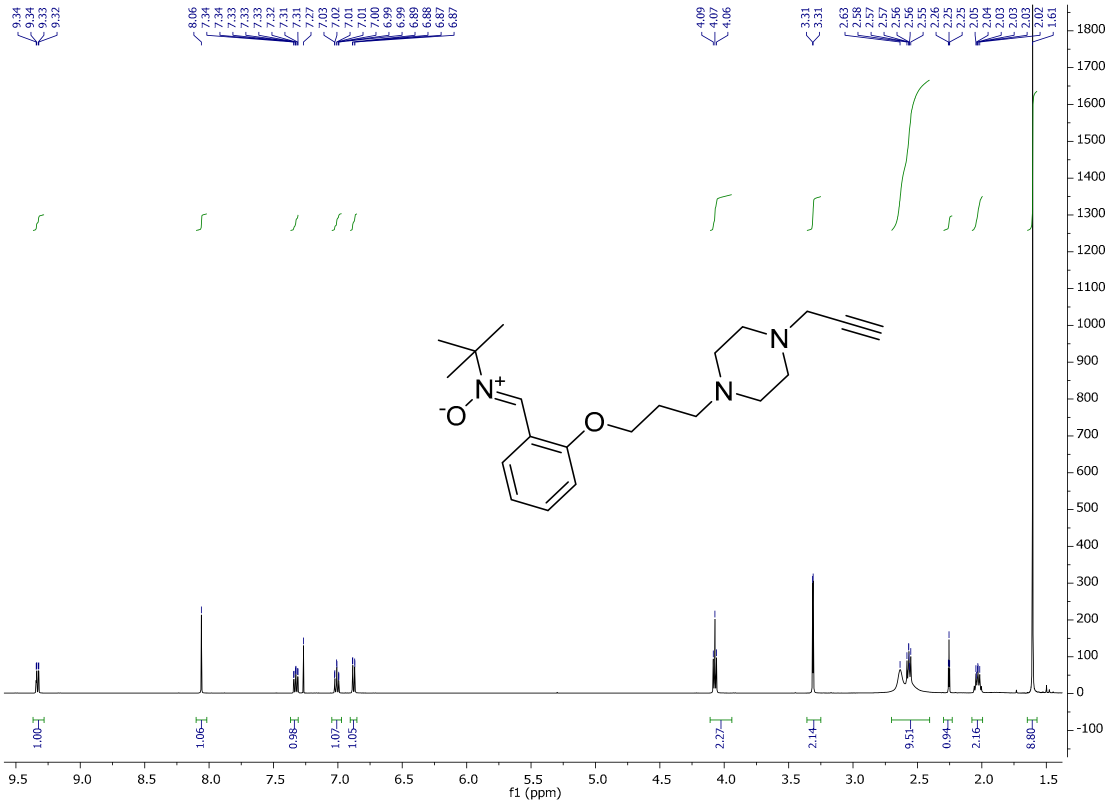
9.34 9.34 9.33 9.32
8.06 7.34 7.34 7.33 7.33 7.33 7.32 7.31 7.31 7.27 7.03 7.02 7.01 7.01 7.00 6.99 6.99 6.89 6.88 6.87 6.87
4.09 4.07 4.06
3.31 3.31
2.63 2.58 2.57 2.57 2.56 2.56 2.55 2.26 2.25 2.25 2.05 2.04 2.03 2.03 2.03 2.02 1.61
1.00 1.06 0.98 1.07 1.05 2.27 2.14 9.51 0.94 2.16 8.80
N
N
O
N+
-O
f1 (ppm)

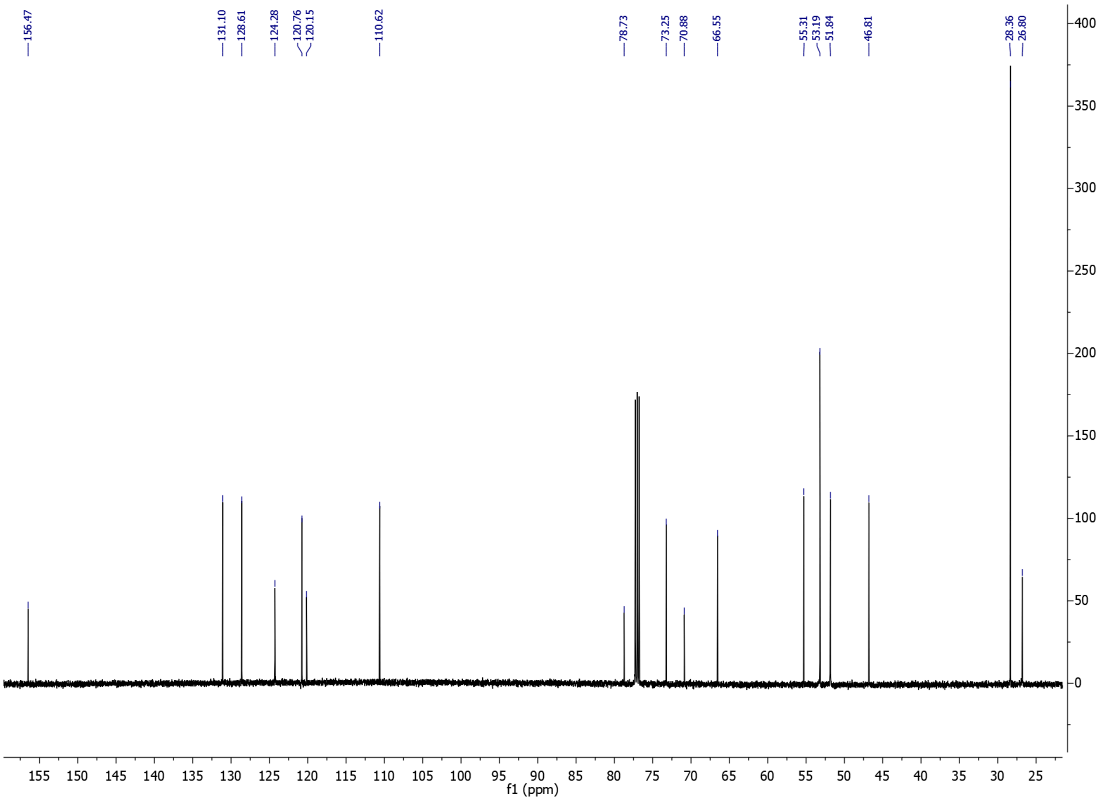
156.47
131.10 128.61 124.28 120.76 120.15
110.62
78.73 73.25 70.88 66.55
55.31 53.19 51.84
46.81
28.36 26.80
f1 (ppm)

Qualitative Compound Report

**Data File**	512_DDI137_01.d	**Sample Name**	DDI137
**Sample Type**	Sample	**Position**	Vial 5
**Instrument Name**	Instrument 1	**User Name**	
**Acq Method**	ESI_ACN_75_pos_new.m	**Acquired Time**	5/27/2021 12:04:56 PM (UTC+01:00)
**IRM Calibration Status**	Success	**DA Method**	Defecto_modificado.m
**Comment**			

**Sample Group**		**Info.**	
**User**	DANIEL DIEZ	**Stream Name**	LC 1
**Acquisition Time (Local)**	5/27/2021 12:04:56 PM (UTC+01:00)	**Acquisition SW Version**	6200 series TOF/6500 series Q-TOF B.08.00 (B8058.3 SP1)
**QTOF Driver Version**	8.00.00	**QTOF Firmware Version**	2.712
**Tune Mass Range Max.**	1700		

Compound Table

Compound Label	RT	Mass	Abund	Formula	Tgt Mass	Diff (ppm)	Hits (DB)
Cpd 1: C21 H31 N3 O2; 2.478	2.478	357.2418	153829	C21 H31 N3 O2	357.2416	0.35	1

Compound Label	*m/z*	RT	Algorithm	Mass
Cpd 1: C21 H31 N3 O2; 2.478	358.249	2.478	Find by Formula	357.2418

MS Zoomed Spectrum

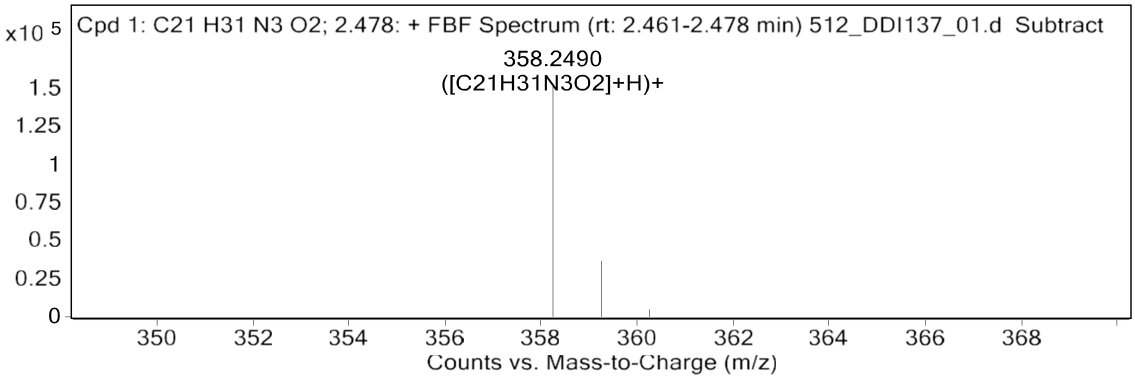

MS Spectrum Peak List

m/z	z	Abund	Formula	Ion
358.249	1	153829.17	C21H31N3O2	(M+H)+
359.2522	1	36791.59	C21H31N3O2	(M+H)+
360.2542	1	4901.46	C21H31N3O2	(M+H)+

MS Zoomed Spectrum

Qualitative Compound Report

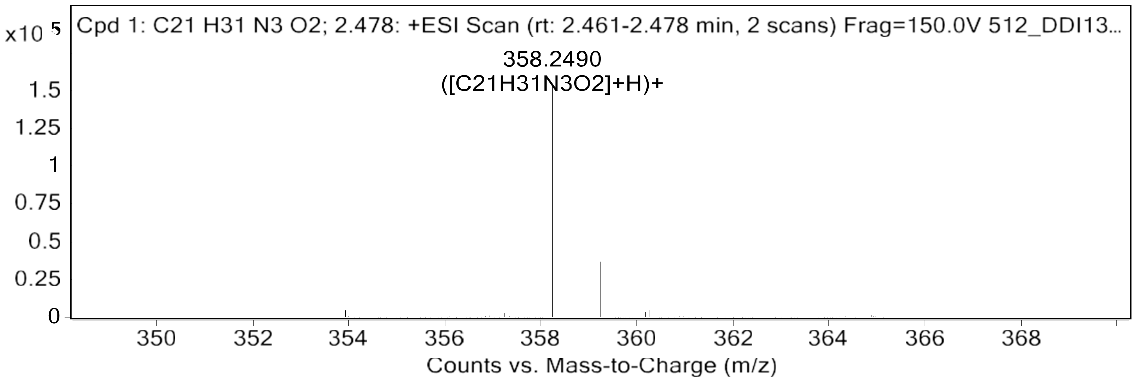

MS Spectrum Peak List

m/z	*Calc m/z*	Diff(ppm)	z	Abund	Formula	Ion
358.249	358.2489	0.39	1	153829.17	C21H31N3O2	(M+H)+
359.2522	359.252	0.48	1	36791.59	C21H31N3O2	(M+H)+
360.2542	360.2549	-1.77	1	4901.46	C21H31N3O2	(M+H)+

--- End Of Report ---
